# Transcriptome-wide mapping of signaling pathways and early immune responses in lumpfish leukocytes upon *in vitro* bacterial exposure

**DOI:** 10.1038/s41598-018-23667-x

**Published:** 2018-03-27

**Authors:** Håvard Ø. Eggestøl, Harald S. Lunde, Anita Rønneseth, David Fredman, Kjell Petersen, Charitra K. Mishra, Tomasz Furmanek, Duncan J. Colquhoun, Heidrun I. Wergeland, Gyri T. Haugland

**Affiliations:** 1 Department of Biology, University of Bergen, Bergen, Norway; 2 Computational biology unit, Department of Informatics, University of Bergen, Bergen, Norway; 3 Norwegian Veterinary Institute, Oslo, Norway

## Abstract

We performed RNA sequencing, identified components of the immune system and mapped early immune responses of lumpfish (*Cyclopterus lumpus*) leukocytes following *in vitro* exposure to the pathogenic bacterium *Vibrio anguillarum* O1. This is the first characterization of immune molecules in lumpfish at the gene level. *In silico* analyses revealed that genes encoding proteins involved in pathogen recognition, cell signaling and cytokines in mammals and teleosts are conserved in lumpfish. Unique molecules were also identified. Pathogen recognition components include 13 TLRs, several NLRs and complement factors. Transcriptome-wide analyses of immune responses 6 and 24 hours post bacterial exposure revealed differential expression of 9033 and 15225 genes, respectively. These included TLR5S, IL-1β, IL-8, IL-6, TNFα, IL-17A/F3, IL-17C and several components of the complement system. The data generated will be valuable for comparative studies and make an important basis for further functional analyses of immune and pathogenicity mechanisms. Such knowledge is also important for design of immunoprophylactic measures in lumpfish, a species of fish now farmed intensively for use as cleaner-fish in Atlantic salmon (*Salmo salar*) aquaculture.

## Introduction

Teleost fish, the earliest evolutionary group with an immune system exerting both innate and adaptive immunity, is highly diverse, consisting of more than 32 000 species. The innate immune system in fish, like mammals, consists of a variety of molecules and immune cells that provide the first line of defense against microbial attack through recognition of potential pathogens. Recognition and degradation of microbes followed by induction of inflammation are essential processes for clearance of microbes and onset of adaptive immune responses. The innate immune system is triggered by complement factors, antibodies and/ or pattern recognition receptor (PRR) recognition of pathogen-associated molecular patterns (PAMPs) such as nucleic acid structures unique to bacteria and virus (CpG DNA, dsRNA), diverse proteins (flagellin), lipopolysaccharide, lipoteichoic acid and peptidoglycan.

While recognition of potential pathogens by complement factors and antibodies lead to increased phagocytic activity of host cells and degradation of invading microbes, recognition of PAMPs by PRRs ensures, through production of cytokines, that the elicited immune response is tailored to the invading pathogen. The major families of PRRs are the Toll-like receptors (TLRs), Nucleotide binding and oligomerization domain (NOD)- like receptors (NLRs), retinoic acid inducible gene I (RIG-I)- like receptors (RLRs), C-type lectin receptors (CLRs) and absent in melanoma 2 (AIM2)- like receptors (ALRs)^1,2^. In teleost fish, the TLRs is the most studied family of the PRRs and an enormous diversity has been identified in teleosts (reviewed in^1,3,4^). This diversity is suggested to be driven by adaptation to specific environments and host-intrinsic factors^3^. Teleosts possess orthologues to mammalian TLRs, with the exception of TLR6 and TLR10 which have not yet been identified in fish and the existence of a functional TLR4 in fish is subject to discussion. In addition, several TLRs are unique for the teleosteii i.e. TLR18-23, 25–28^4,5^. Fish and amphibians also have a soluble version of TLR5, termed TLR5S^6^ in addition to a membrane bound TLR5 (TLR5M). TLR5 has been identified in all investigated teleost species, with the exception of the *Paracanthopterygii*^7^. From functional studies and functional inference based on sequence homology indicate that fish TLR1, TLR2, TLR5, TLR5S, TLR9, TLR21, TLR28^5,8,9^ recognize bacterial ligands. In general, ligand binding initiates downstream cell signaling mediated via adapter proteins MyD88, MAL, TRIF, TRAM and SARM^10^, resulting in activation of transcription factors NFκB, IRF3/7, CREB and AP1, finally resulting in production of proinflammatory cytokines like TNFα, IL-12 IL-1β and IL-18 and/or interferons. There is currently little information regarding the downstream cell signaling pathways following activation of the fish-specific TLRs.

As for the TLR family, some NLRs also play a role in antimicrobial immune responses. NOD-like receptors are described in several species of fish including, but not exclusively, zebrafish, channel catfish, Japanese pufferfish and rainbow trout^11–16^. The NLRs described in fish are NOD1, NOD2, NLRC3, NLRC5, NLRCX and NLRC. Importantly, NLRC in fish is different from mammalian NLRC and as many as several hundred genes have been reported from one species^13^. There are, however, few functional studies of NLRs in fish and there is currently little knowledge of the downstream signaling after activation and how the receptors and signaling are regulated.

The transcriptome of lumpfish, as a representative for *Cyclopteridae* is highly valuable as this group is poorly characterized genetically and no reference genome or immune gene sequences are available in public databases. Also, it is not clear whether they belong to the suborder *Cottoidei* within the order *Perciformes*^17^ or within the order *Scorpaniformes*^18^. In addition to being interesting for comparative studies, mapping of the lumpfish immune system is important for basic immunological studies and for the rational design of immunoprophylactic measures for this species. In recent years, there has been a tremendous increase in the production of farmed lumpfish in Europe and Canada^19^, due to its ability to eat lice from farmed Atlantic salmon (*Salmo salar* L.)^20^. In Norway alone, the number of lumpfish farmed increased from 0.4 million in 2012 to approximately 15 million in 2016^21^.

Large scale farmed lumpfish mortalities due to bacterial disease are reported^22^ and development of vaccines protecting against the most common pathogens is ongoing^23^. The level of total immunoglobulin M (IgM) in lumpfish sera is lower compared to species like salmon^24,25^, but it has been shown that lumpfish has the ability to produce specific antibodies upon immunization^25^ and that vaccination has an effect^26^. Previous studies have also shown that innate immune functions like phagocytosis and respiratory burst are efficient in lumpfish^27^ and that IgM^+^ B-cells display phagocytic ability^25^. More knowledge about the underlying mechanisms of the immune system of lumpfish at the individual gene level and their immune responses upon bacterial infection is required as this will form the basis for development of immunoprophylactic measures and immune stimulation. Therefore, to characterize the immediate and early induced innate response in this species, lumpfish leukocytes were exposed to the bacterium *Vibrio anguillarum* serotype O1, a known fish pathogen, for 6 and 24 hours, and RNA sequencing was performed followed by *de novo* transcriptome assembly and differential gene expression analysis.

## Results

### Illumina sequencing and de novo transcriptome assembly

Sequencing of RNA isolated from non-treated head kidney leukocytes (HKL) and HKL exposed to *Vibrio* resulted in 516 million reads. Reads of low quality, low complexity, containing adapter sequence, matching ribosomal or mitochondrial sequences were discarded. The resulting transcriptome consisted of 433 million assembled bases in 346,430 transcripts from 221,659 trinity genes. The median transcript length was 585 bases, mean length 1.25 kb and N50 of 2.5 kb. The RNA sequencing reads after trimming, the differential gene expression data and the assembled transcriptome are submitted to Array Express under accession number E-MTAB-6388.

### Annotation of predicted proteins and functional annotation of the Trinity genes

**Figure 1:**
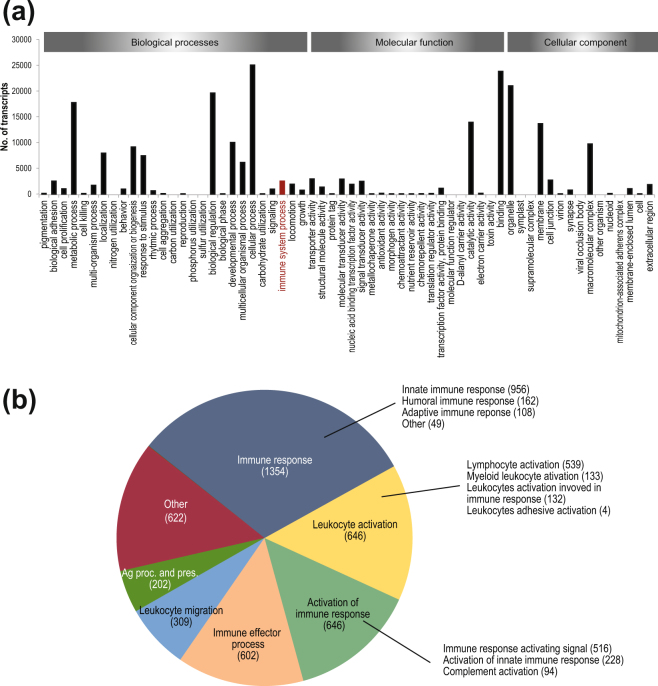
Gene Ontology (GO) analyses of annotated genes in the lumpfish transcriptome. (**a**) The annotated genes were divided into the main GO-terms Biological processes, Molecular function and cellular components and further divided into subcategories. (**b**) Pie chart of the GO term distribution among the annotated genes in the lumpfish transcriptome in the GO term immune system process.

Genes within the assembled transcriptome were annotated using Trinotate. Putative gene functions were identified by Gene ontology (GO) analysis. Of the 221,659 Trinity genes 37,895 were assigned minimum one Gene ontology (GO)-term. GO mapping resulted in 62 GO categories presented in Fig. 1. The GO-terms containing the highest number of genes were binding (23786), organelle (20995), cellular process (25011) and biological regulation (19578). The GO-term ‘immune system processes’ contained 2490 Trinity genes and includes genes involved in the development or function of the immune system e.g. immune response, leukocyte activation, activation of immune response and immune effector process (Fig. 1b). The most abundant immune system process was “innate immune response” which included 956 genes.

### Global differential gene expression (DEG) analysis upon bacterial exposure

**Figure 2:**
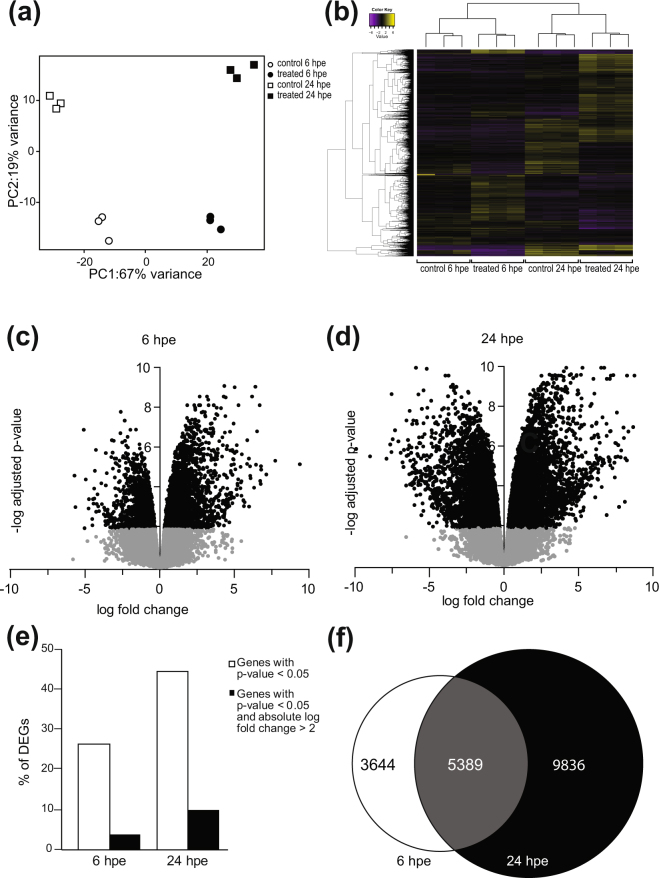
Differential gene expression (DEG) analysis 6 hrs and 24 hrs post bacterial exposure. (**a**) Principal component analysis. PC1 is time and PC2 is treatment. White circles are non-treated controls 6 hpe, black circles are treated samples 6 hpe, white squares are non-treated controls 24 hpe and black circles are treated samples 24 hpe. (**b**) Heatmap of transcriptome profiling data of non-treated controls versus bacterial exposed samples 6 and 24 hpe. (**c**) Volcano plot of DEGs 6 hpe. Significantly regulated genes are shown as black dots. Non-significantly regulated genes are shown as grey dots. (**d**) Volcano plot of DEGs 24 hpe. Significantly regulated genes are shown as black dots. Non-significantly regulated genes are shown as grey dots. (**e**) Percentage of DEGs that were significantly regulated (p-value < 0.05) at 6 hpe and 24 hpe are shown in black bars. Percentages of statistically significantly regulated (p-value < 0.05) DEG with an absolute log fold change >2. (**f**) Venn diagram showing the number of DEGs at the different time point. Only those that were statistically significant are shown. White = 6 hpe, black = 24 hpe and dark grey = genes that were significantly regulated at both time points.

To gain more information of the early induced innate immune responses in lumpfish, leukocytes were subjected to differential gene expression (DEG) analysis 6 and 24 hrs post bacterial exposure. Principal component analysis (Fig. 2a) revealed a major difference between exposed and non-exposed samples at both time points. This can be seen in the heat map following hierarchical clustering of the DEGs (Fig. 2b). The immune response was stronger and more extensive at 24 hours post exposure (hpe) (Fig. 2c) compared to 6 hpe (Fig. 2d). The number of statistically (p-value < 0.05) and biologically (p-value < 0.05 and fold change >4) significantly regulated genes was higher at 24 hpe compared to 6 hpe (Fig. 2e). The number of genes that were statistically differentially expressed at 24 hpe was 15225 genes (44%) compared to 9033 genes (26%) 6 hpe (Fig. 2e and f). As shown in the Venn diagram, 5389 (16%) genes were significantly differentially expressed at both time points (Fig. 2f).

**Figure 3:**
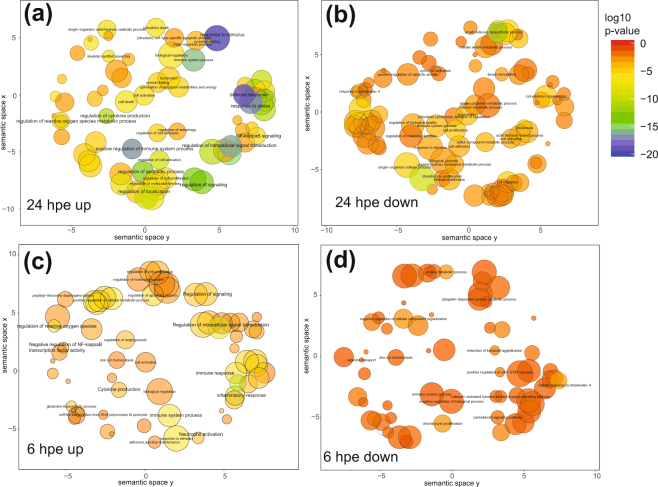
Enrich GO-analysis 6 and 24 hours post bacterial exposure. Semantic plots of up and down regulated (log fold change >2 and p-value < 0.001) enriched GO terms at 6 and 24 hours post exposure, generated through REVIGO. Enrichment p-value is plotted in red, through yellow and green to blue; where blue is the smallest p-value and red the biggest p-value. Size of the circles correlates to the semantic size of the GO terms.

GO enrichment analysis showed that among the upregulated transcripts at 24 hpe, GO-terms with lowest p-value were; response to stimulus (log10 p-value −19.7), defense response (log10 p-value −18.9), response to stress (log10 p-value −17.1), positive regulation of immune system processes (log10 p-value −16.6) and regulation of intracellular signal transduction (log10 p-value −16.1) (Fig. 3a). Among downregulated transcripts at 24 hpe, the GO-terms with lowest p-value were; “small molecule biosynthetic process” (log10 p-value −11.3), “single-organism process” (log10 p-value −9.8), “response to interleukin 4” (log10 p-value −7.9), “cytokinesis” (log10 p-value −7.3) and “defense response“(log10 p-value −7.0) (Fig. 3b). For upregulated transcripts at 6 hpe, the GO-terms with lowest p-values were; “response to lipopolysaccharide” (log10 p-value −10.4), “inflammatory response” (log10 p-value −9.7), “response to biotic stimulus” (log10 p-value −9.4), “regulation of intracellular signal transduction” (log10 p-value −8.1) and “response to external stimulus” (log10 p-value −8.1) (Fig. 3c). For downregulated transcripts at 6 hpe, the p-values were not as low as at 24 hpe (Fig. 3d).

Analyses of KEGG pathways belonging to the immune system were performed (Table 1). Several genes were identified for each KEGG ID, and thus, the number of lumpfish genes in DEG was higher than the number of KEGG IDs in DEG (Table 1). Further, the 20 most significantly regulated genes at 24 and 6 hpe (based on p-values) were identified (Supplemental Table 1). At 24 hours, the most significantly regulated gene was TLR5S, followed by interleukin 8 (IL-8) which is also known as neutrophil chemotactic factor and an uncharacterized protein. From blast search the uncharacterized protein likely belongs to the interleukin 6 (IL-6) family, most closely related to Leukemia Inhibitory factor (LIF) (Supplemental Table 1).

**Table 1:** Overview of identified lumpfish genes in immune system pathways*.

KEGG pathway	KEGG ID	No. of KEGG IDs in reference pathway	No. of KEGG IDs in DEG	No. of lumpfish genes in DEG	6 hpe		24 hpe	
					Upreg. genes	Downreg. genes	Upreg. genes	Downreg. genes
Hematopoietic cell lineage	K04640	80	35	70	10	19	36	18
Complement and coagulation cascades	K04610	78	33	57	10	15	23	18
Platelet activation	K04611	89	73	223	43	34	69	74
Toll-like receptor signaling pathway	K04620	76	54	124	28	41	29	56
Toll and Imd signaling pathway	K04624	47	21	74	12	20	13	29
NOD-like receptor signaling pathway	K04621	136	92	239	48	66	46	105
RIG-I-like receptor signaling pathway	K04622	53	39	96	22	31	22	45
Cytosolic DNA-sensing pathway	K04623	51	29	44	11	15	13	17
Natural killer cell mediated cytotoxicity	K04650	81	41	146	33	31	52	45
Antigen processing and presentation	K04612	41	27	71	5	21	18	19
T cell receptor signaling pathway	K04660	85	59	206	36	48	55	86
Th1 and Th2 cell differentiation	K04658	67	44	116	19	42	36	43
B cell receptor signaling pathway	K04662	57	42	122	30	31	32	54
Fc epsilon RI signaling pathway	K04664	47	31	98	25	20	27	38
Fc gamma R-mediated phagocytosis	K04666	58	48	211	41	46	73	71
Leukocyte transendothelial migration	K04670	75	58	171	27	47	59	57
Intestinal immune network for IgA prod.	K04672	37	14	32	6	9	12	7
Chemokine signaling pathway	K04062	153	83	259	53	64	73	102

^*^KEGG pathways in category 5.1.

The 50 most up- and down-regulated genes at each time point were identified (Supplemental Tables 2–5). Many of the upregulated immune genes at 24 hpe were cytokines such as IL-1β, IL-6, IL-8 and IL-17, or belonged to either the complement cascade (CFH, CFB, C8a, C8b and C5) or the TLR pathway (TLR5s). Other studies have shown that members of the NLR family of pattern recognition receptors also recognize bacterial antigens and regulation of genes encoding these receptors was investigated. The response of NOD1, NOD2 and other NLRs were very weakly regulated or non-responsive (data not shown). Since the most regulated genes belonged to the complement cascade and TLR signaling, these pathways were investigated at the individual gene level.

### Complement cascade

**Figure 4:**
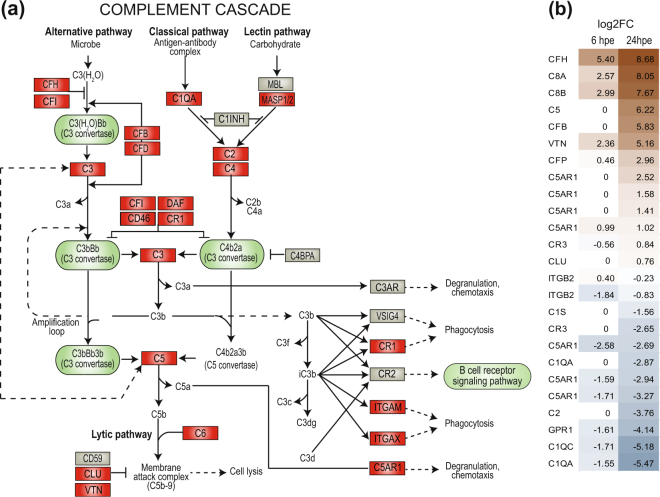
An overview of the complement cascade in lumpfish (**a**) The molecules in the complement cascade identified in lumpfish are shown with red boxes, those that are not yet identified are shown in grey. The figure is modified from KEGG map04610^63^. (**b**) Differential gene expression analyses of members of the complement cascade 6 hrs and 24 hrs post exposure (hpe). Only those that are statistically significant regulated (p-value < 0.05) are shown. The color gradient represents highly upregulated (dark brown) to highly downregulated (dark blue) genes. The exact values are given for each gene. The genes are sorted by fold regulation at 24 hpe.

The complement system can be activated by three biochemical pathways; the classical complement pathway, the alternative complement pathway and the lectin pathway. Many genes encoding complement proteins were identified in lumpfish (shown in Fig. 4a and listed in Table 1), including components such as C3, C6 and C7. The differential gene expression analyses showed that upon exposure to *V. anguillarum* complement factor responses were higher at 24 hpe compared to 6hpe (Fig. 4b). The most upregulated genes were the regulatory factors complement factor H (CFH) and complement factor B (CFB), complement components C5, vitronectin (VTN) and complement factors 8a and 8b. The latter are subunits of the membrane attack complex responsible for lysis of microbes. Also, complement factor P, which is a positive regulator for C3 and C5 convertases was also upregulated at 24 hpe. The most highly downregulated genes were complement C1q subcomponent subunit A (C1QA) and subunit C (C1QC) which are part of the classical pathway, in addition to complement components C2 (Fig. 4b). Lumpfish genes verified (by blast) as belonging to the complement cascade are given in Supplemental Table 6.

### TLRs and TLR signaling

**Figure 5:**
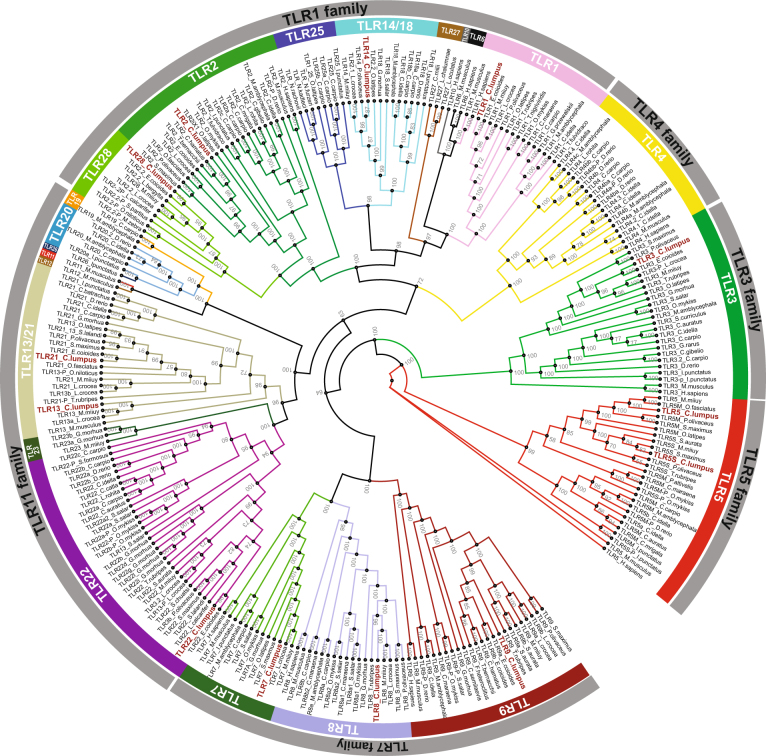
Phylogenetic tree of TLRs. Full-length TLR sequences in public databases were included in the phylogenetic analyses. The TLRs are divided into families and subtypes. The TLRs identified in the lumpfish transcriptome is shown by red letters, including TLR1, −2, −3, −5 (membrane-bound and soluble), −7, −8, −9, −13, −14, −21, −22 and −28. The full-length name of the species and accession numbers of the sequences in the Figure is given in Supplemental Tables 7 and 8.

**Figure 6:**
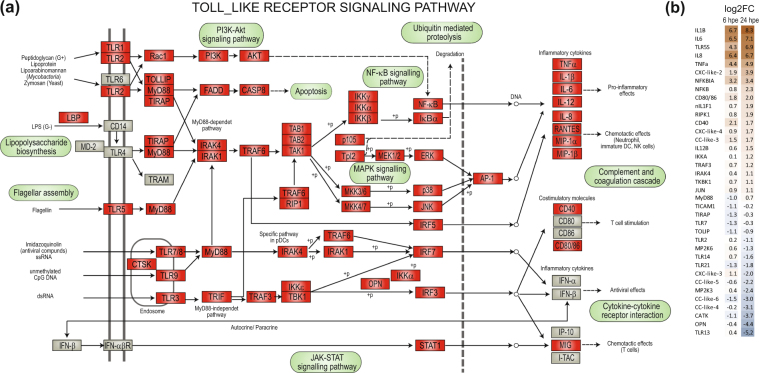
An overview of the Toll-like receptor signaling pathway in lumpfish (**a**) The molecules in the TLR signaling pathway identified in lumpfish are shown with red boxes, those that are not yet identified are shown in grey. The figure is modified from KEGG map04620^63^. (**b**) Differential gene expression analyses of members of the TLR pathway 6 hrs and 24 hrs post exposure (hpe). Only those that are statistically significant regulated (p-value < 0.05) are shown. The color gradient represents highly upregulated (dark brown) to highly downregulated (dark blue) genes. The exact values are given for each gene. The genes are sorted by fold regulation at 24 hpe.

The TLR family of signaling PRRs plays an essential role in the early innate immune response against both bacteria and viruses. In the lumpfish transcriptome, 13 TLRs were identified; TLR1, 2, 3, 5 M, 5 S, 7, 8, 9, 13, 14, 21, 22 and 28 (Fig. 5, Table 2). Activation of TLRs initiates intracellular signaling resulting in production of inflammatory cytokines and co-stimulatory molecules important in early pro-inflammatory responses, chemotaxis and activation of T cells. Many of the molecules involved in the TLR signaling pathway were identified in lumpfish (Fig. 6), including the adaptor proteins MyD88, TRIF (also known as TICAM1) and TIRAP (also known as MAL). TICAM 2 (TRAM) was not identified. All transcripts listed in Table 2 were annotated following a BLAST search against NCBI’s non-redundant database, for which the hit with highest total score is included in the Table. MyD88, TRIF and TIRAP were full-length, but for SARM only two short non-overlapping fragments were identified.

**Table 2:** Verified TLRs in lumpfish and genes in TLR signaling pathway.

Gene-ID	Name	KEGG ID	Top BLAST hit			
			Description	E-value	Species	Accession number
**Pathogen recognition receptors**						
TR65368|c1_g16	TLR1	K05398	Toll-like receptor 1	0	*Notothenia coriiceps*	XP_010775742.1
TR39054|c0_g2	TLR2	K10159	Toll-like receptor 2	0	*Oplegnathus fasciatus*	AFZ81806
TR25266|c0_g1	TLR3	K05401	Toll-like receptor 3	0	*Epinephelus coioides*	AEX01718
TR27403|c4_g1	TLR5M	K10168	toll-like receptor 5 membrane bound	0	*Oplegnathus fasciatus*	AQT26515
TR41627|c0_g1	TLR5S	K10168	PRED: toll-like receptor 5	0	*Notothenia coriiceps*	XP_010788825
TR35019|c2_g2	TLR7	K05404	PRED: toll-like receptor 7	0	*Notothenia coriiceps*	XP_010771824
TR35019|c2_g2	TLR8	K10170	PRED: toll-like receptor 7	0	*Notothenia coriiceps*	XP_010771824
TR74757|c0_g1	TLR9	K10161	Toll-like receptor 9B	0	*Epinephelus lanceolatus*	AJW66344
TR14442|c0_g1	TLR13	—	Toll-like receptor 13	0	*Lates calcarifer*	XP_018537347
TR7225|c0_g1	TLR14	—	Toll-like receptor 14	0	*Larimichtys crocea*	XP_010735448
TR59969|c0_g1	TLR21	—	Toll-like receptor 21	0	*Epinephelus lanceolatus*	AJW66342
TR32827|c0_g1	TLR22	—	Toll-like receptor 22	0	*Epinephelus coioides*	AGA84053
TR50658|c1_g2	TLR28	—	Toll-like receptor 2–2	0	*Epinephelus coioides*	AIS23533
TR22563|c0_g3	LBP/BPI	K05399	Bactericidal permeability-increasing protein	0	*Oplegnathus fasciatus*	BAM21037
**Intracellular signaling molecules**						
TR12120|c0_g1	AKT1	K04456	Unnamed protein product, partial	3.22E-34	*Tetraodon nigroviridis*	CAG10696
TR31506|c0_g2	CASP8	K04398	Caspase-8-like	1.82E-76	*Labrus bergylta*	XP_020505530
TR59882|c1_g1	FADD	K02373	FAS-associated death domain protein-like	1.75E-36	*Lates calcarifer*	XP_018527571
TR33817|c1_g1	IKKa	K04467	Inhibitor of NFk-B kinase subunit alpha-like	1.04E-39	*Labrus bergylta*	XP_020482453
TR52372|c4_g2 TR71389|c0_g1	IKKb IKKb	K07209 K04734	IKKbeta IKKbeta alpha	4.37E-161 7.36E-128	*Siniperca chuatsi* *Epinephelus coioides*	ADK47101 AKN59236
TR27462|c0_g1	IKKE	K07211	PRED: inhibitor of NFkappa-B kinase E	0	*Lates calcarifer*	XP_018542264
TR109249|c0_g1	IRAK1	K04730	Interleukin-1 receptor activated kinase 1	3.98E-34	*Siniperca chuatsi*	ACN64942
TR49087|c0_g1	IRAK4	K04733	Interleukin-1 receptor-associated kinase 4	9.13E-35	*Trachidermus fasciatus*	AFH88675
TR16021|c2_g2	IRF3	K05411	Interferon regulatory factor 3	1.07E-57	*Dicentrarchus labrax*	CBN81356
TR53466|c0_g1	IRF5	K09446	Interferon regulatory factor 5	4.38E-108	*Oplegnathus fasciatus*	AFZ93894
TR129437|c0_g1	IRF7	K09447	Interferon regulatory factor 7	0	*Epinephelus coioides*	ADA57613
TR80028|c2_g8	M3K7	K04427	PRED: MAP3K7_isoform X1	1.66E-05	*Stegastes partitus*	XP_008299748
TR129360|c0_g1	MAP3K8	K04415	PRED: MAP3K8	6.49E-47	*Notothenia coriiceps*	XP_010779244
TR10769|c1_g13	MK01	K04371	PRED: MAPkinase 1	4.28E-09	*Pundamilia nyererei*	XP_005730582
TR83303|c0_g1	MK08	K04440	MAPkinase 8B	1.15E-88	*Larimichthys crocea*	KKF10666
TR8373|c0_g1	MP2K1	K04368	Dual specificity MAPkinase kinase 1-like	2.94E-18	*Oncorhynchus kisutch*	XP_020331169
TR24160|c1_g1	MP2K2	K04369	PRED: dual specificity MAP kinase kinase 2	1.30E-70	*Stegastes partitus*	XP_008275716
TR24160|c1_g1	MP2K3	K04430	Dual specificity MAP kinase kinase 4	0	*Larimichthys crocea*	KKF28316
TR10914|c0_g1	MP2K4	K04430	Dual specificity MAP kinase kinase 4-like	0	*Monopterus albus*	XP_020467371
TR11220|c0_g1	MP2K6	K04433	PRED: dual specificity MAP kinase kinase 6-like	0	*Larimichthys crocea*	XP_019116692
TR69482|c2_g12	MP2K7	K04431	PRED: dual specificity MAP kinase kinase 7	3.15E-21	*Notothenia coriiceps*	XP_010776556
TR70736|c1_g1	MyD88	K04729	Myeloid differentiation factor 88	1.14E-163	*Oplegnathus fasciatus*	AQT26514
TR52312|c2_g4	NEMO	K07210	NFkappa-B kinase essential modifier 2	0	*Epinephelus coioides*	AKN59239
TR19609|c0_g2	NFKB1	K02580	PRED: nuclear factor NF-kappa-B p100 subunit	5.71E-13	*Astyanax mexicanus*	XP_007258829
TR105668|c0_g1	P3KCA	K00922	PRED: PIK3 catalytic subunit gamma isoform-like	8.43E-70	*Notothenia coriiceps*	XP_010777483
TR102536|c0_g1	P85A	K02649	PIK3 regulatory subunit alpha-like, partial	7.35E-62	*Labrus bergylta*	XP_020514940
TR34005|c0_g1	PMK1	K04441	PRED: MAPkinase 11-like isoform X2	2.63E-45	*Salmo salar*	XP_014008787
TR106991|c0_g1	RAC1	K04392	Unnamed protein product, partial	3.34E-11	*Mus musculus*	BAC38272
TR24024|c0_g3	RIPK1	K02861	PRED: serine/threonine-protein kinase Nek8-like	2.01E-118	*Lates calcarifer*	XP_018555349
TR18988|c3_g2	STAT1	K11220	PRED: STAT1-alpha/beta isoform X4	1.81E-109	*Larimichthys crocea*	XP_010745394
TR101399|c0_g1	TAB1	K04403	PRED: TAB1	7.18E-18	*Paralichthys olivaceus*	XP_019958222
TR18998|c1_g2	TAB2	K04404	TAK1-binding protein 2	0	*Epinephelus coioides*	AKN59234
TR86999|c0_g2	TBK1	K05410	PRED: serine/threonine-protein kinase TBK1	1.05E-09	*Larimichthys crocea*	XP_019126730
TR33723|c0_g2	TF65	K04735	p65 transcription factor	5.99E-93	*Siniperca chuatsi*	ABW84004
TR1276|c0_g1	TICAM1	K05842	PRED: TIR domain-containing adapter molecule 1	0	*Larimichthys crocea*	XP_010736595
TR53144|c0_g1	TIRAP	K05403	PRED: TIRAP	2.27E-77	*Lates calcarifer*	XP_018554351
TR15941|c0_g6	TOLLIP	K05402	PRED: toll-interacting protein-like, partial	4.72E-57	*Notothenia coriiceps*	XP_010779552
TR27389|c3_g2	TRAF3	K03174	PRED: TNF receptor-associated factor 3	1.0E-148	*Lutjanus sanguineus*	APJ7747
TR49717|c0_g1	TRAF6	K03175	TNF receptor-associated factor 6, partial	4.00E-174	*Gasterosteus aculeatus*	ABJ15863
***Extracellular signaling molecules***						
TR102531|c0_g1	CC-like	K14625	PRED: C-C motif chemokine 17-like	9.90E-60	*Notothenia coriiceps*	XP_010784217
TR155750|c0_g1	CC-like	K05512	PRED: C-C motif chemokine 26-like	3.34E-26	*Cynoglossus semilaevis*	XP_008332070
TR71759|c1_g1	CC-like	K12964	PRED: monocyte chemotactic protein 1B-like	1.28E-34	*Oreochromis niloticus*	XP_019216385
TR1773|c1_g1	CC-like	K12964	C-C motif chemokine 14 precursor	1.22E-28	*Anoplopoma fimbria*	ACQ58688
TR4483|c0_g1	CC-like	K16595	PRED: C-C motif chemokine 4 homolog	1.53E-27	*Lates calcarifer*	XP_018542538
TR26820|c0_g1	CC-like	K12964	C-C motif chemokine 3 precursor	3.86E-48	*Anoplopoma fimbria*	ACQ58878
TR135792|c0_g1	CXC-like	K05416	C-X-C motif chemokine 10 precursor	3.00E-56	*Anoplopoma fimbria*	ACQ59055
TR88050|c0_g1	CXC-like	NA	PRED: C-X-C motif chemokine 11-like	1.12E-41	*Stegastes partitus*	XP_008294834
TR19700|c0_g1	CXC-like	K05506	Interleukin-8 like protein	5.53E-39	*Oplegnathus fasciatus*	BAM99883
TR25958|c0_g1	IL12A	K05406	PRED: uncharacterized protein LOC109630380	7.13E-71	*Paralichthys olivaceus*	XP_019944119
TR24065|c1_g2	IL12B	K05425	Interleukin 12p40	1.80E-54	*Oplegnathus fasciatus*	AIB04025
TR14360|c3_g2	IL1B	K04519	Interleukin-1 beta	8.98E-138	*Trachidermus fasciatus*	AFH88676
TR87818|c0_g1	IL6	K05405	Interleukin-6	3.04E-94	*Epinephelus coioides*	AFE62919
TR13890|c0_g3	IL8	K10030	Interleukin-8 precursor	2.95E-33	*Anoplopoma fimbria*	ACQ57874
TR50382|c0_g2	JUN	K04448	PRED: transcription factor AP-1-like	4.29E-62	*Notothenia coriiceps*	XP_010795740
TR29865|c0_g1	nIL1F1	NA	New interleukin-1 family member, partial	5.58E-57	*Gasterosteus aculeatus*	CCV66728
TR69814|c0_g2	TNFa	K03156	Tumor necrosis factor alpha	4.71E-120	*Oplegnathus fasciatus*	ACM69339
TR42972|c0_g1	FOS	K04379	PRED: proto-oncogene c-Fos-like isoform X1	9.07E-89	*Larimichthys crocea*	XP_010733543
TR37206|c0_g2	CD40	K03160	TNF receptor superfam member 5-like isoform X2	1.51E-26	*Labrus bergylta*	XP_020504780
TR1121|c5_g7	CD80/86	K05413	PRED: CD276 antigen-like	1.53E-78	*Lates calcarifer*	XP_018537117

^*^Pathway: ko04620.

Members of the tumor necrosis factor receptor (TNFR)-associated factor (TRAF) family are important mediators of various signaling pathways, including the TLR signaling pathway. The TRAFs identified in lumpfish were TRAF2-6. Further, IRAK1, 3 and 4 were identified. Also, main components of the two downstream signaling routes, NF-κB signaling; NEMO, IKKA, IKKB, IKB, p50 and p65 and MAPK-signaling pathways; MKKs, ERK, JNK, p38, c-fos Jun were identified (Fig. 6 and Table 2). Components that were not mapped through batch mapping in the KEGG pathway database were searched for manually in the lumpfish transcriptome using synonyms or sequences from related species. Activation of NF-κB induces production of the pro-inflammatory cytokines, while activation of MAPK has impact on several immune functions including proliferation, differentiation, survival, apoptosis, chemoattraction and production of inflammatory mediators. TNFα, IL-1β, IL-6 and IL-12 were among the cytokines identified in the lumpfish transcriptome. Also, the chemokines IL-8 and MIP1 β (macrophage inflammatory protein, also known as CCL4) were identified. The genes most upregulated at both 6 hpe and 24 hpe included proinflammatory cytokines (IL-1β, IL-6, TNFα), a homologue of IL-17 (IL-17C1), IL-8 and the soluble form of TLR5 (TLR5S) (Supplemental Tables 2 and 4). Members of the NFκβ pathway, but not the MAPK pathway were upregulated (Fig. 6b). Interestingly, another IL-17 homologue (IL17A/F3) was one of the most down-regulated immune genes at 24 hpe (Supplemental Table 3). TLR13 (logFC −5.21) and TLR2 (logFC −2.74) were down-regulated at 24 hpe and 6 hpe, respectively.

## Discussion

The innate immune system is of major importance for fish as aquatic vertebrates are generally more heavily exposed to pathogens than terrestrial vertebrates and the adaptive defenses are less efficient in aquatic vertebrates. The major humoral components essential for innate defense in vertebrates are antibodies and the complement system which tag and kill invading microbes and promote inflammatory responses^28^. Furthermore, conserved structures on potential pathogenic organisms such as flagellin are recognized by the host’s PRRs and trigger intracellular signaling pathways which results in production of inflammatory cytokines and initiation of adaptive immune responses tailored to the infecting agent.

To obtain information of the gene repertoire in lumpfish head kidney leukocytes and early anti-bacterial immune responses, leukocytes were exposed to the pathogenic bacterium *V.anguillarum* O1 and RNA was isolated 6 and 24 hpe. *De novo* transcriptome assembly and global differential gene expression revealed that the complement system and TLR signaling pathway were the most highly upregulated innate immune processes. Another family of PRRs involved in bacterial recognition is NLRs. *In vivo* challenge experiments in other teleost species have shown that expression of NOD1 and NOD2 is upregulated in several tissues after bacterial infection^29,30^. Lumpfish NLRs were either non-regulated or weakly downregulated. The functions and roles of NLRs in lumpfish upon bacterial infection should, therefore, be further explored. The ligand specificity of the expanded fish-specific NLRC family reported from several fish species is currently unknown and it will be exciting to elucidate their role and importance in fish immunity.

Several components of the complement cascade were identified within the lumpfish transcriptome as shown in Fig. 4. Most genes belonging to the classical and alternative pathway were identified, but not the mannose-binding lectin (MBL) involved in the lectin pathway. Of the complement receptors, CR1, CR3, CR4 and C5AR1 were identified, but not complement receptor CR2. This is similar to other fish species (summarized in^28^). In humans it is known that the complement system cross-talks with other pathways and modulates adaptive immune responses^31,32^. Information regarding cross-talk between pathways and involvement of B and T cells in fish are scarce, and discrimination of some components, in example C1r/C1s, requires functional analyses at the protein level.

In the lumpfish transcriptome, TLR1, −2, −3, −5 (membrane-bound and soluble), −7, −8, −9, −13, −14, −21, −22 and −28 were identified. All lumpfish TLR transcripts, with the exception of TLR22, encoded full-length sequences. Phylogenetic analyses (Fig. 5) show that lumpfish TLRs group together with the order *Perciformes*, most closely with orange-spotted grouper (*Epinephelus coioides*). While some teleost TLRs are orthologues of mammalian counterparts, equivalents to human TLR6 and TLR10 have not yet been found in fish. Many of the TLRs in fish are not present in mammals. These include TLR5S, −14, −18, −19, −20, −21, −22, −23, −24, −25, −26, −27 and −28, and of these, some are fish-specific (TLR18-23, 25–28). The soluble variant of TLR5 is widely present in teleosts and has been identified in several species such as rainbow trout^33^, catfish^34,35^, gilthead seabream^36^, flounder^37^ and orange spotted grouper^38^. Since *V. anguillarum* is a flagellated bacterium, it was not unexpected that TLR5S was highly upregulated during early immune responses. Actually, it was the most significantly regulated gene at 24 hpe and among the most significantly upregulated genes at 6 hpe (Supplemental Table 1). In lumpfish leukocytes, TLR5M was not significantly regulated either at 6 hpe or 24 hpe. This is similar to the situation in rainbow trout where expression of TLR5S, but not TLR5M, was induced by *V.anguillarum* and purified recombinant *V.anguillarum* flagellin^33^. Upregulation of TLR5S transcripts during bacterial exposure is reported in other fish species^35,36^. Humans do not have TLR5S, but the innate immune response to flagellin mediated by human TLR5M is similar to that of teleost fishes^39^. Interestingly, a study of Tsujita and colleagues showed that TLR5S from rainbow trout amplifies the human TLR5 response via physical binding to flagellin^40^. How TLR5S initiates downstream signaling is not yet known, but a hypothetical mechanism has been suggested in which TLR5S binds circulating flagellin and transports it to TLR5M. In this way danger signals are amplified in a similar manner to LPS recognition by human TLR4 and the soluble factors LBP and CD14^41^. It is known that activation of TLR5 in mammals results in activation of NF-kappa-B and production of proinflammatory cytokines. The DEG analyses of lumpfish leukocytes indicated that the NF-κB signaling pathway, not the MAPK signaling pathway, was activated, as inhibitors of both nuclear factor kappa-B kinase alpha (NFKBIA, also known as IκBα and IKKA) and NFκB were highly upregulated. DEG analysis showed that IKKA was upregulated at both 6 hpe and 24 hpe, while NFkB was most highly upregulated at 24 hpe. Gene expressions of transcripts involved in the MAPK signaling pathway, such as MP2K3 and MP2K6, showed little change (after 6 hpe) or were downregulated (after 24 hpe). It will be interesting to investigate whether regulation of the TLR5 signaling pathway is conserved, or whether teleosts have developed another regulatory mechanism than mammals.

The cytokines that were most differentially regulated were IL-1β, IL-8, IL-6, TNF-α, one of the IL-17A/F3 and IL17C1. All were highly upregulated, except IL17A/F3 which was barely differentially regulated at 6 hpe and highly downregulated at 24 hpe. IL-1β has diverse functions including being a major regulator of inflammatory processes. It is a chemoattractant for fish leukocytes, it stimulates chemokine production in cells following infection and is known to induce expression of TNF-α. Further, IL-1β also modulates differentiation of T helper 17 cells (Th17) and expression of IL-17 family members^42,43^. Th17 cells are a subset of activated CD4 + T cells and are known to play a role in mucosal immunity and tissue inflammation. In mice, in addition to IL-1β, IL-6 and the transcription factor RORγt, transforming growth factor β1 (TGF-β1) is required for differentiation of Th17 cells. In humans, RORγt and Th17 polarization was induced by IL-1β and enhanced by IL-6, but suppressed by TGF-β1 and IL-12^44^. Although the exact regulation of Th17 cells in fish is not yet understood, it is widely accepted that fish have Th17 cells as all the major components of mammalian Th17 cell development are present in fish, including Th17 driver cytokines (IL-6, TGF- β1, IL-21 and IL-23), transcription factor (RORγ) and effector cytokines (IL17A/F, IL-22)^42,45^. Since some of the Th17 components in fish have multiple isoforms, it has been suggested that an even more complex Th17 type responses and regulation are present in fish compared to mammals^45^.

The most highly regulated cytokine in lumpfish leukocytes following bacterial exposure belonged to the IL-17 family. IL-17A/F3 was highly downregulated 24 hpe, while one of the IL-17C proteins, IL-17C1, was the most upregulated transcript, at 6 hpe and 24 hpe. IL-17 cytokines are central mediators of inflammatory responses and have been functionally characterized in jawed and jawless vertebrates and in invertebrates such as molluscs, nematodes and arthropods^46–51^. Teleost fish have several IL-17 molecules including IL17A and IL-17F, termed IL17A/F1-3, IL-17B, IL17C and IL17D^42,52^. One IL-17 originally termed IL-17N^53^ is likely to represent a fourth IL-17A/F member. An IL17E equivalent has thus far not been identified in fish, but two IL17C genes have been reported in rainbow trout^48^ and Japanese pufferfish^54^. Two IL-17C-like genes were also identified in lumpfish, but no IL-17E. It has been suggested that an ancient IL17C may have diverged into IL-17C and IL-17E in early mammals, whereas two IL-17C genes can be present in teleosts. Although relatively few studies have reported bioactivity of the IL-17 molecules in fish, studies from different species suggest that while IL-17 proteins play a role in innate immunity, they may have evolved specialized roles. Recombinant IL-17A/F from grass carp and trout can increase expression of proinflammatory cytokines in isolated head kidney leukocytes^55^ and splenocytes^56^, respectively, while IL-17D in grass carp increase expression of IL-1β, IL-8, TNF-α but not IL-6 (reviewed in^42^).

In summary, our transcriptomic data suggests that the complement system recognized the pathogenic bacterium and activated subunits of the membrane attack complex (MAC) which is a prerequisite for formation of a MAC complex at the surface of the microbe and thereafter cell lysis. Also, complement receptors involved in phagocytosis, degranulation and chemotaxis were upregulated which is related to the need to recruit host phagocytic cells for clearance of the bacterium. One of the most highly upregulated genes was IL-8 which is a chemokine involved in chemotaxis and attraction of neutrophilic cells. Another immediate innate immune response essential to prevent infection is promotion of inflammation and production of cytokines that ensures the immune response is tailored to the infecting microbe. Our study suggests that TLR5S recognized flagellin and triggered downstream signaling through the NFk-B signaling pathway resulting in production of pro-inflammatory cytokines (IL-1β, TNFα, IL-6, IL-12 and IL-17). IL-12 is needed for activation of naïve T-cells and IL-17 induces production of chemokines. Our transcriptomic data adds valuable information about the immune responses in lumpfish during the early stages of a bacterial infection. Functional analysis of the proteins involved in the signaling pathways is however necessary to gain further insight into the role of specific proteins and the interaction between them.

The lumpfish transcriptome presented provides a valuable base for comparative and phylogenetic analyses as lumpfish is a representative of the infraorder *Cottoidea*, a phylogenetic group which is poorly characterized immunologically and genetically. Furthermore, the lumpfish is a novel and a very important species for aquaculture since it is used for sea-lice control in salmon farming^19^. Although production of lumpfish has generally been successful, there have been challenges with large-scale mortality due to bacterial infections^22^. Vaccines against selected lumpfish pathogens are in use^23,26^, but more knowledge of the lumpfish immune system and responses to bacterial exposure at the individual gene level is important. Thus, the identification of immune genes, transcriptome–wide mapping of signaling pathways and early immune responses presented here are highly valuable as they provide a basis for development of more efficient immune prophylactic measures and provide important tools for evaluation of the efficacy of different prophylactic measures.

## Materials and Methods

The work in the presented manuscript was performed on cells isolated from dead fish. The fish were sacrificed with a sharp blow to the head which is an appropriate procedure under Norwegian law. All experiments were performed in accordance with relevant guidelines and regulations. Rearing of fish under normal, optimal conditions does not require ethical approval under Norwegian law (FOR 1996- 01- 15 no. 23)

### Fish

Farmed lumpfish (*C. lumpus* L.) were provided from Fjord Forsk Sogn AS, a commercial breeder in Sogn & Fjordane County, Norway. The fish were kept in a 500 L tank at the Aquatic and Industrial Laboratory (ILAB) within the High-Technology Centre in Bergen under normal rearing conditions with a light regime 12 h light: 12 h dark. The water temperature was 8 °C, salinity 34 PSU and a minimum of 77% oxygen saturation in the outlet water. The fish were fed with the commercial dry feed Amber Neptune (1.5 mm).

### Bacterial culture

*Vibrio anguillarum* serotype O1 (8752) isolated from moribund lumpfish after a disease-outbreak in 2012 in Møre & Romsdal county in Norway was cultured in tryptic soy broth containing 2% NaCl at 20 °C, 200 rpm until late log phase. The bacterium was washed once in PBS and re-suspended in L-15 + medium without antibiotics.

### Isolation of leukocytes and *in vitro* bacterial exposure

Head kidney leukocytes were isolated as described previously using discontinuous Percoll gradients^27^. Both left and right kidney lobes from 15 fish were included. Cell number, viability and aggregation factor was determined using a CASY Cell Counter™ (Innovatis AG). For *in vitro* bacterial exposure, 5 × 10^6^ cells in L-15 + medium without antibiotics were added to each well in a 24-well plate (Nunc) and mixed with the bacterium *V. anguillarum* O1 (MOI 1:10) in a total volume of 0.5 mL. In wells with non-exposed cells, medium was added instead of bacterial cells. The plates were incubated at 15 °C. After 1.5 hour, pencillin/streptomycin was added to each well and the plates were further incubated until 6 hrs and 24 hours post bacterial exposure. In order to obtain an as comprehensive transcriptome as possible, a sample with leukocytes exposed with infectious pancreatic necrosis virus for 24 hrs was also included. This sample was used for the *de novo* transcriptome assembly, but was not part of the DEG analysis. Following incubation, the plates were centrifuged for 10 min at 200 × g. The supernatants were removed and lysis buffer was added directly to the wells. The lysates were stored at −80 °C prior to RNA isolation.

### Isolation of total RNA

Total RNA was isolated using GeneElute Mammalian Total RNA miniprep kit (Sigma) according to the manufacturer’s instructions. Samples were treated with DNase I (Sigma) to removed traces of genomic DNA and the concentration of total RNA determined in a Nanodrop^®^ND-1000 UV-Vis spectrophotometer (Nanodrop Technologies). Total RNA extracts from three-five fish were pooled, in total 5 µg per pooled sample. For each time point three parallels were prepared for RNA sequencing. The pooled RNA (5 µg) was cleaned using RNA clean & concentrator-5 (zymo research) according to the manufacturer’s instructions and the quality of the RNA were determined in an Agilent 2100 bioanalyzer. RNA isolated from virus infected leukocytes was kept separately. The RQI values were in the range 6.3–9.3.

### Transcriptome sequencing, assembly and annotation

The Norwegian High Throughput Sequencing Centre prepared sequencing libraries using TruSeq™RNA sample Preparation kit (Illumina®) according to the manufacturer’s protocol and performed paired-end strand-specific sequencing on the Illumina HiSeq platform with a 125 bp read length, resulting in a total of 516 million reads. Read quality was first assessed using FastQC, and Trinity’s option for read trimming by quality was included during assembly (trimmomatic). Reads of low quality, low complexity, containing adapter sequence, matching ribosomal or mitochondrial sequences were discarded. Transcripts were assembled using Trinity v2.0.6^57^ with read normalization enabled and library type specified, otherwise keeping default settings. Known contaminants (*Vibrio* and IPNV) were removed from the assembly using blast. During the analyses, other non-eukaryotic sequences were discovered and additionally removed from the expression value matrices, with a more generic contaminant removal procedure^58^. More information on all steps of the sequencing data processing is given in Supplemental methods. The resulting transcriptome consisted of 433 million assembled bases in 346,430 transcripts from 221,659 “genes”. The median transcript length was 585 bases, mean length 1.25 kb and N50 of 2.5 kb. Following assembly transcripts were annotated with BLAST matches, protein domains and GO terms using the Trinotate toolkit (https://trinotate.github.io).

### Bioinformatical analyses

Gene ontology mapping was performed in J-express Gene expression analysis software. Detailed information about the gene included in each category was obtained using Quick GO, which is a fast browser for Gene Ontology terms and annotation (http://www.ebi.ac.uk/QuickGO/GTerm?id=GO:0006954#term=annotation). Verification of the annotation of the transcripts was performed with BLAST search (https://blast.ncbi.nlm.nih.gov/Blast.cgi), multiple sequence alignment (MSA) using PAGAN v.0.61^51^. The phylogenetic tree was constructed from MSA by maximum likelihood with IQ-TREE 1.5.4^59^ using automatic model selection^60^ followed by 100,000 ultrafast bootstraps^61^. An overview of the species and accession numbers included in the phylogenetic analyses are given in Supplemental Tables 7 and 8, respectively. Pathway analyses were performed using KEGG^61–63^. KEGG pathways analysis^64^ was performed by annotating the transcripts using BLAST against KO genes in KEGG, downloaded 08.02.2017. Transcripts with a BLAST score of 300 and above against KO genes in KEGG were mapped to the KEGG pathways as described in the KEGG Mapper tool. Transcript abundances for three biological replicates for treatment and control at 6 and 24 hpe were estimated using RSEM as part of the Trinity pipeline (Supplementary results of Trinity RSEM). The read count estimates were used as a basis for differential expression analysis using the Limma R-package^65^. Only genes with at least 10 reads in at least three samples were considered for differential expression analysis (34280 of 221659 assembled genes). Fold changes between groups and adjusted p-values (BH correction for multiple testing) were exported for downstream analyses. The DEG analyses were visualized in Graph-Pad prism 5. GO enrichment was calculated using GO-seq.^66^ and visualized in REVIGO.

The datasets generated during the current study are available in Array Express repository.

## Electronic supplementary material

Supplemental information

## References

[1] Aoki T, Hikima J, Hwang SD, Jung TS (2013). Innate immunity of finfish: primordial conservation and function of viral RNA sensors in teleosts. Fish Shellfish Immunol.

[2] Brubaker SW, Bonham KS, Zanoni I, Kagan JC (2015). Innate immune pattern recognition: a cell biological perspective. Annu Rev Immunol.

[3] Solbakken MH, Voje KL, Jakobsen KS, Jentoft S (2017). Linking species habitat and past palaeoclimatic events to evolution of the teleost innate immune system. Proc Biol Sci.

[4] Zhang J (2014). Toll-like receptor recognition of bacteria in fish: ligand specificity and signal pathways. Fish Shellfish Immunol.

[5] Wang Y, Li J, Han J, Shu C, Xu T (2016). Identification and characteristic analysis of TLR28: A novel member of the TLR1 family in teleost. Dev Comp Immunol.

[6] Roach JC (2005). The evolution of vertebrate Toll-like receptors. Proc Natl Acad Sci USA.

[7] Solbakken MH, Rise ML, Jakobsen KS, Jentoft S (2016). Successive Losses of Central Immune Genes Characterize the Gadiformes’ Alternate Immunity. Genome Biol Evol.

[8] Pietretti D, Wiegertjes GF (2014). Ligand specificities of Toll-like receptors in fish: indications from infection studies. Dev Comp Immunol.

[9] Quiniou SM, Boudinot P, Bengten E (2013). Comprehensive survey and genomic characterization of Toll-like receptors (TLRs) in channel catfish, *Ictalurus punctatus*: identification of novel fish TLRs. Immunogenetics.

[10] O’Neill LA, Bowie AG (2007). The family of five: TIR-domain-containing adaptors in Toll-like receptor signalling. Nat Rev Immunol.

[11] Alvarez CA (2017). Insights into the diversity of NOD-like receptors: Identification and expression analysis of NLRC3, NLRC5 and NLRX1 in rainbow trout. Mol Immunol.

[12] Biswas G, Bilen S, Kono T, Sakai M, Hikima J (2016). Inflammatory immune response by lipopolysaccharide-responsive nucleotide binding oligomerization domain (NOD)-like receptors in the Japanese pufferfish (*Takifugu rubripes*). Dev Comp Immunol.

[13] Howe K (2016). Structure and evolutionary history of a large family of NLR proteins in the zebrafish. Open Biol.

[14] Laing KJ, Purcell MK, Winton JR, Hansen JD (2008). A genomic view of the NOD-like receptor family in teleost fish: identification of a novel NLR subfamily in zebrafish. BMC Evol Biol.

[15] Rajendran KV (2012). Pathogen recognition receptors in channel catfish: I. Identification, phylogeny and expression of NOD-like receptors. Dev Comp Immunol.

[16] Stein C, Caccamo M, Laird G, Leptin M (2007). Conservation and divergence of gene families encoding components of innate immune response systems in zebrafish. Genome Biol.

[17] Betancur-R. R. *et al*. The tree of life and a new classification of bony fishes. *PLoS Curr* **5** ecurrents.tol.53ba26640df0ccaee75bb165c8c26288 (2013). 10.1371/currents.tol.53ba26640df0ccaee75bb165c8c26288 PMC3644299 23653398

[18] Nelson, J. S., Grande, T. C. & Wilson, M. V. H. *Fishes of the world, 5th Edition*. Order *Scorpaniformes*, 467–495 (Wiley, 2016).

[19] Powell, A. *et al*. Use of lumpfish for sea-lice control in salmon farming: challenges and opportunities. *Reviews in aquaculture*10.1111/raq.12194 (2017).

[20] Imsland AK (2014). The use of lumpfish (*Cyclopterus lumpus* L.) to control sea lice (*Lepeophtheirus salmonis* Krøyer) infestations in intensively farmed Atlantic salmon (*Salmo salar* L.). Aquaculture.

[21] Norwegian Directorate of Fisheries. Sales of farmed cleanerfish 2012-2016. http://www.fiskeridir.no/English/Aquaculture/Statistics/Cleanerfish-Lumpfish-and-Wrasse (2017).

[22] Hjeltnes, B., Bornø, G., Jansen, M. D., Haukaas, A. & Walde, C. S. The Health Situation in Norwegian Aquaculture 2016 (2017).

[23] Haugland, G. T., Rønneseth, A. & Wergeland, H. I. in *Cleaner Fish biology and aquaculture application* (ed Jim Treasurer) Immunology and vaccinology of lumpfish and wrasse (5m Publishing, *in print*).

[24] Bilal S, Lie KK, Karlsen OA, Hordvik I (2016). Characterization of IgM in Norwegian cleaner fish (lumpfish and wrasses). Fish Shellfish Immunol.

[25] Rønneseth A, Ghebretnsae DB, Wergeland HI, Haugland GT (2015). Functional characterization of IgM^+^ B cells and adaptive immunity in lumpfish (*Cyclopterus lumpus* L.). Dev Comp Immunol.

[26] Rønneseth A, Haugland GT, Colquhoun DJ, Brudal E, Wergeland HI (2017). Protection and antibody reactivity following vaccination of lumpfish (*Cyclopterus lumpus* L.) against atypical Aeromonas salmonicida. Fish Shellfish Immunol.

[27] Haugland GT (2012). Phagocytosis and respiratory burst activity in lumpsucker (*Cyclopterus lumpus* L.) leucocytes analysed by flow cytometry. PLoS One.

[28] Nakao M, Tsujikura M, Ichiki S, Vo TK, Somamoto T (2011). The complement system in teleost fish: progress of post-homolog-hunting researches. Dev Comp Immunol.

[29] Li J, Gao Y, Xu T (2015). Comparative genomic and evolution of vertebrate NOD1 and NOD2 genes and their immune response in miiuy croaker. Fish Shellfish Immunol.

[30] Li M (2012). Expression profiles of NODs in channel catfish (*Ictalurus punctatus*) after infection with *Edwardsiella tarda*, *Aeromonas hydrophila*, *Streptococcus iniae* and channel catfish hemorrhage reovirus. Fish Shellfish Immunol.

[31] Kemper C, Atkinson JP (2007). T-cell regulation: with complements from innate immunity. Nat Rev Immunol.

[32] Ricklin D, Hajishengallis G, Yang K, Lambris JD (2010). Complement: a key system for immune surveillance and homeostasis. Nat Immunol.

[33] Tsujita T (2004). Sensing bacterial flagellin by membrane and soluble orthologs of Toll-like receptor 5 in rainbow trout (*Onchorhynchus mykiss*). J Biol Chem.

[34] Baoprasertkul, P., Xu, P., Peatman, E., Kucuktas, H. & Liu, Z. Divergent Toll-like receptors in catfish (*Ictalurus punctatus*): TLR5S, TLR20, TLR21. *Fish Shellfish Immunol* **23** (2007). 10.1016/j.fsi.2007.06.002 17981052

[35] Jayaramu PK (2017). Studies on expression pattern of toll-like receptor 5 (TLR5) in *Edwardsiella tarda* infected *Pangasianodon hypophthalmus*. Fish Shellfish Immunol.

[36] Munoz I, Sepulcre MP, Meseguer J, Mulero V (2013). Molecular cloning, phylogenetic analysis and functional characterization of soluble Toll-like receptor 5 in gilthead seabream, *Sparus aurata*. Fish Shellfish Immunol.

[37] Moon, J. Y. *et al*. Maximal transcriptional activation of piscine soluble Toll-like receptor 5 by the NF-kappaB subunit p65 and flagellin. *Fish Shellfish Immunol* **31** (2011). 10.1016/j.fsi.2011.08.002 21867757

[38] Bai JS (2017). Molecular identification and expression analysis of TLR5M and TLR5S from orange-spotted grouper (*Epinepheluscoioides*). Fish Shellfish Immunol.

[39] Hayashi F (2001). The innate immune response to bacterial flagellin is mediated by Toll-like receptor 5. Nature.

[40] Tsujita T (2006). Fish soluble Toll-like receptor (TLR)5 amplifies human TLR5 response via physical binding to flagellin. Vaccine.

[41] Rebl A, Goldammer T, Seyfert HM (2010). Toll-like receptor signaling in bony fish. Vet Immunol Immunopathol.

[42] Secombes, C. J., Wang, T. & Bird, S. in *The evolution of the immune system* (ed D. Malagoli) Vertebrate cytokines and their evolution, 87–150 (Elsevier, 2016).

[43] Zou J, Secombes CJ (2016). The Function of Fish Cytokines. Biology (Basel).

[44] Acosta-Rodriguez EV, Napolitani G, Lanzavecchia A, Sallusto F (2007). Interleukins 1beta and 6 but not transforming growth factor-beta are essential for the differentiation of interleukin 17-producing human T helper cells. Nat Immunol.

[45] Wang T, Secombes CJ (2013). The cytokine networks of adaptive immunity in fish. Fish Shellfish Immunol.

[46] Das, S. & Khader, S. *Yin and yang of interleukin-17 in host immunity to infection* [version 1; referees: 2 approved]. F1000Research **6** (F1000 Feculty Rev), 741 (2017). 10.12688/f1000research.10862.1 PMC5490359 28713557

[47] Buckley KM (2017). IL17 factors are early regulators in the gut epithelium during inflammatory response to *Vibrio* in the sea urchin larva. Elife.

[48] Wang T, Martin SA, Secombes CJ (2010). Two interleukin-17C-like genes exist in rainbow trout *Oncorhynchus mykiss* that are differentially expressed and modulated. Dev Comp Immunol.

[49] Lee J (2001). IL-17E, a novel proinflammatory ligand for the IL-17 receptor homolog IL-17Rh1. J Biol Chem.

[50] Li H (2000). Cloning and characterization of IL-17B and IL-17C, two new members of the IL-17 cytokine family. Proc Natl Acad Sci USA.

[51] Starnes T, Broxmeyer HE, Robertson MJ, Hromas R (2002). Cutting edge: IL-17D, a novel member of the IL-17 family, stimulates cytokine production and inhibits hemopoiesis. J Immunol.

[52] Kono T, Korenaga H, Sakai M (2011). Genomics of fish IL-17 ligand and receptors: a review. Fish Shellfish Immunol.

[53] Wang T (2015). Identification of the salmonid IL-17A/F1a/b, IL-17A/F2b, IL-17A/F3 and IL-17N genes and analysis of their expression following *in vitro* stimulation and infection. Immunogenetics.

[54] Korenaga H, Kono T, Sakai M (2010). Isolation of seven IL-17 family genes from the Japanese pufferfish Takifugu rubripes. Fish Shellfish Immunol.

[55] Du L (2015). Identification and functional characterization of grass carp IL-17A/F1: An evaluation of the immunoregulatory role of teleost IL-17A/F1. Dev Comp Immunol.

[56] Monte MM, Wang T, Holland JW, Zou J, Secombes CJ (2013). Cloning and characterization of rainbow trout interleukin-17A/F2 (IL-17A/F2) and IL-17 receptor A: expression during infection and bioactivity of recombinant IL-17A/F2. Infect Immun.

[57] Grabherr MG (2011). Full-length transcriptome assembly from RNA-Seq data without a reference genome. Nat Biotechnol.

[58] Gladyshev EA, Meselson M, Arkhipova IR (2008). Massive horizontal gene transfer in bdelloid rotifers. Science.

[59] Nguyen LT, Schmidt HA, von Haeseler A, Minh BQ (2015). IQ-TREE: a fast and effective stochastic algorithm for estimating maximum-likelihood phylogenies. Mol Biol Evol.

[60] Kalyaanamoorthy S, Minh BQ, Wong TKF, Von Haeseler A, Jermiin LS (2017). ModelFinder: Fast model selection for accurate phylogenetic estimates. Nat Methods.

[61] Minh BQ, Nguyen MA, von Haeseler A (2013). Ultrafast approximation for phylogenetic bootstrap. Mol Biol Evol.

[62] Kanehisa M, Furumichi M, Tanabe M, Sato Y, Morishima K (2017). KEGG: new perspectives on genomes, pathways, diseases and drugs. Nucleic Acids Res.

[63] Kanehisa M, Goto S (2000). KEGG: Kyoto Encyclopedia of Genes and Genomes. Nucleic Acids Res..

[64] Kanehisa M, Goto S, Sato Y, Furumichi M, Tanabe M (2012). KEGG for integration and interpretation of large-scale molecular data sets. Nucleic Acids Res.

[65] Ritchie ME (2015). Limma powers differential expression analyses for RNA-sequencing and microarray studies. Nucleic Acids Res..

[66] Young, M. D., Wakefield, M. J., Smyth, G. K. & Oshlack, A. Goseq: Gene Ontology Testing for RNA-seq Datasets. https://bioconductor.org/packages/devel/bioc/vignettes/goseq/inst/doc/goseq.pdf (2012).

