# Supplementary material for: Transcriptome-wide mapping of signaling pathways and early immune responses in lumpfish leukocytes upon in vitro bacterial exposure
Source: Sci Rep. 2018 Mar 27;8:5261. doi: 10.1038/s41598-018-23667-x (PMC5869700; doi:10.1038/s41598-018-23667-x)
Supplement: Supplementary file 1 — Supplemental information [file 41598_2018_23667_MOESM1_ESM.pdf]

Transcriptome-wide mapping of signaling pathways and early immune responses in lumpfish leukocytes upon *in vitro* bacterial exposure

Håvard Ø. Eggestøl[1], Harald S. Lunde[1], Anita Rønneseth[1], David Fredman[2], Kjell Petersen[2], Charitra K. Mishra[2], Tomasz Furmanek[2], Duncan J. Colquhoun[1,3], Heidrun I. Wergeland[1] and Gyri T. Haugland[1]*.

[1]Department of Biology, University of Bergen, Bergen, Norway
[2]Computational Biology Unit, Department of Informatics, University of Bergen, Bergen, Norway
[3]Norwegian Veterinary Institute, Oslo, Norway
* Gyri.Haugland@bio.uib.no

Supplementary materials:

Supplemental Table 1. Overview of the most significantly regulated genes at 6 and 24 hpe……………………………………...…2
Supplemental Table 2. Overview of the 50 most up-regulated genes at 24 hpe (sorted by logFC)…………………...……….4
Supplemental Table 3. Overview of the 50 most down-regulated genes at 24 hpe (sorted by logFC)…………..…………....6
Supplemental Table 4. Overview of the 50 most up-regulated genes at 6 hpe (sorted by logFC)…………………….………8
Supplemental Table 5. Overview of the 50 most down-regulated genes at 6 hpe (sorted by logFC)………….…………..10
Supplemental Table 6. Overview of verified lumpfish genes in the complement cascade………………………………..……12
Supplemental Table 7. Overview of the species included in the phylogenetic analyses……………………………….….……14
Supplemental Table 8: Accession numbers of genes in the phylogenetic analyses of TLRs ………………………….….……16
Supplementary Methods for Assembly and Annotation………………………………………………………….………………….25
Supplementary Figure 1……….26
Supplementary Methods for Bioinformatic scripts……………………………………………………………….……………….27
Supplementary Results of Trinity RSEM…………………………………………………………………………….………….51

Supplemental Table 1. Overview of the most significantly regulated genes at 6 and 24 hpe (sorted by adjpval) *

Most significantly regulated genes at 24 hpe						
gene_id	logFC_24h	adjpval_24h	Annotations	Full record name	Species	E-value
TR41627	6.90	9.11E-11	TLR5	Toll-like_receptor_5	*Homo sapiens*	6E-92
TR13890	6.73	1.16E-10	IL8	Interleukin-8-like	*Serola dumerili*	6E-21
TR87025	5.27	1.16E-10	Uncharacterized	PREDICTED: uncharacterized protein LOC108879818	*Lates calcarifer*	5E-73
TR41114	6.52	1.16E-10	C1QL3	Complement_C1q-like_protein_3	*Mus musculus*	2E-12
TR41155	4.19	1.16E-10	NFAT5	Nuclear_factor_of_activated_T-cells_5	*Mus musculus*	8E-23
TR53083	-5.90	1.16E-10	MRC1	Macrophage_mannose_receptor_1	*Mus musculus*	0
TR70021	-5.18	1.30E-10	SIAE	Sialate_O-acetylesterase	*Mus musculus*	1E-19
TR22616	4.01	1.74E-10	HS74L	Heat_shock_70_kDa_protein_4L	*Homo sapiens*	5E-170
TR81788	4.50	2.43E-10	DDX24	ATP-dependent_R._helicase_DDX24	*Pongo*	4E-41
TR52303	3.06	2.53E-10	IL12R-B2	Interleukin-12 receptor subunit beta-2-like	*Notothenia coriiceps*	3E-141
TR69283	5.17	2.56E-10	Hypothetical	hypothetical protein Z043_122910	*Scleropages formosus*	9E-05
TR79163	7.34	2.67E-10	TRFE	Serotransferrin	*Oryzias latipes*	0
TR8937	3.43	2.67E-10	CEP192	PREDICTED: centrosomal protein of 192 kDa	*Larimichtys crocea*	0E+00
TR71746	4.12	2.67E-10	NFAT5	Nuclear_factor_of_activated_T-cells_5	*Homo sapiens*	3E-58
TR70589	4.88	2.67E-10	PTGES	Prostaglandin_E_synthase	*Equus caballus*	4E-28
TR37961	4.42	2.67E-10	TAF4B	Transcription_initiation_factor_TFIID_subunit_4B	*Mus musculus*	6E-19
TR81776	8.77	2.83E-10	CC chemokine 20	C-C motif chemokine 20 precursor	*Anoplopoma fimbria*	8E-45
TR14360	8.27	2.83E-10	IL1B	Interleukin-1_beta	*Oncorhynchus mykiss*	9E-73
TR8934	3.21	2.83E-10	MAST3	Microtubule-associated_serine/threonine-protein_kinase_3	*Xenopus laevis*	4E-107
TR65236	3.68	2.83E-10	TIMP2	Metalloproteinase_inhibitor_2	*Cavia porcellus*	7E-109
Most significantly regulated genes at 6 hpe						
gene_id	logFC_6h	adjpval_6h	Annotations	Full record name	Specie	E-value
TR31519	4.31	8.49E-10	NR4A1	Nuclear_receptor_subfamily_4_group_A_member_1	*Xenopus laevis*	6E-52
TR13890	6.39	9.31E-10	IL8	Interleukin-8-like	*Serola dumerili*	6E-21
TR87025	5	1E-09	Uncharacterized	PREDICTED: uncharacterized protein LOC108879818	*Lates calcarifer*	5E-73
TR41627	4.28	2.90E-09	TLR5	Toll-like_receptor_5	*Homo sapiens*	6E-92
TR52303	2.66	2.90E-09	IL12R-B2	Interleukin-12 receptor subunit beta-2-like	*Notothenia coriiceps*	3E-141
TR69283	4.27	2.90E-09	Hypothetical protein	hypothetical protein Z043_122910	*Scleropages formosus*	9E-05
TR81776	6.17	2.90E-09	CC chemokine 20	C-C motif chemokine 20 precursor	*Anoplopoma fimbria*	8E-45
TR28159	3.15	2.90E-09	PTGIS	Prostacyclin_synthase	*Bos taurus*	3E-30
TR86119	4.40	3.17E-09	TNIP2	TNFAIP3-interacting_protein_2	*Mus musculus*	2E-31
TR19609	3.24	4.21E-09	IKBA	NF-kappa-B_inhibitor_alpha	*Gallus gallus*	6E-57
TR49945	5.26	7.93E-09	GL.	Glutamine_synthetase,_mitochondrial	*Squalus acanthias*	6E-22
TR87414	5.19	7.93E-09	GL.	Glutamine_synthetase	*Canis lupus*	2E-147
TR80474	2.48	7.93E-09	SIK2	Serine/threonine-protein_kinase_SIK2	*Mus musculus*	5E-143
TR59888	2.70	7.93E-09	unnamed protein	Unnamed protein product	*Tetraodon nigroviridis*	1.4E-01
TR129039	3.61	7.93E-09	HSP 90-beta-3	Putative heat shock protein HSP 90-beta-3	*Tupaia chinensis*	4E-05
TR1049	1.83	7.93E-09	DDX41	Probable_ATP-dependent_R._helicase_DDX41	*Mus musculus*	0

TR59631	2.61	7.93E-09	FOS	Proto-oncogene_c-Fos	*Takifugu rubripes*	4E-100
TR81788	3.26	7.93E-09	DDX24	ATP-dependent_R._helicase_DDX24	*Pongo*	4E-41
TR14360	6.69	7.93E-09	IL1B	Interleukin-1_beta	*Oncorhynchus mykiss*	9E-73
TR8934	2.65	7.93E-09	MAST3	Microtubule-associated_serine/threonine-protein_kinase_3	*Xenopus laevis*	4E-107

* Positive logFC values= upregulated transcripts, negative logFC values=down-regulated transcripts

Supplemental Table 2. Overview of the 50 most up-regulated genes at 24 hpe (sorted by logFC)

50 most upregulated genes at 24 hpe (sorted by logFC)						
gene_id	logFC_24h	adjpval_24h	Annotations	Full record name	Specie	E-value
TR26702|c0_g1	9.50	1.97E-06	N42L1	NEDD4-binding_protein_2-like_1	*Mus musculus*	3E-07
TR78231|c0_g1	9.07	1.21E-04	GDF15	Growth/differentiation_factor_15	*Mus musculus*	2E-24
TR81776|c4_g4	8.77	2.83E-10	CC chemokine 20	C-C motif chemokine 20 precursor	*Anoplopoma fimbria*	8E-45
TR74252|c0_g1	8.68	1.11E-07	CFAH	Complement_factor_H	*Homo sapiens*	2E-34
TR45367|c1_g1	8.35	1.85E-07	HPT	Haptoglobin	*Sus scrofa*	3E-40
TR14360|c3_g2	8.27	2.83E-10	IL1B	Interleukin-1_beta	*Oncorhynchus mykiss*	9E-73
TR30315|c0_g1	8.05	3.91E-07	CO8A	Complement_component_C8_alpha_chain	*Oryctolagus cuniculus*	1E-157
TR23708|c0_g1	8.03	1.17E-07	NMES1	Normal_mucosa_of_esophagus-specific_gene_1_protein	*Homo sapiens*	2E-23
TR3853|c0_g2	7.83	3.67E-06	BORG1	Cdc42_effector_protein_2	*Homo sapiens*	4E-06
TR27293|c0_g1	7.67	9.05E-07	CO8B	Complement_component_C8_beta_chain	*Paralichthys olivaceus*	0
TR80849|c0_g2	7.59	2.06E-08	SAA1	Serum_amyloid_A-1_protein	*Mus musculus*	3E-11
TR76870|c0_g2	7.53	7.24E-07	EPGN	Epigen	*Gallus gallus*	1E-09
TR67971|c1_g1	7.49	3.57E-06	LPP	Lipoma-preferred_partner_homolog	*Gallus gallus*	1E-28
TR82903|c2_g2	7.42	6.94E-06	PHOS	Phosducin	*Bos taurus*	1E-65
TR79163|c0_g5	7.34	2.67E-10	TRFE	Serotransferrin	*Orizias latipes*	0
TR80347|c0_g1	7.17	2.83E-10	HEMO	Hemopexin_{ECO:0000250|UniProtKB:P20058}	*Danio rerio*	9E-46
TR45367|c1_g2	7.14	4.55E-05	HPT	Haptoglobin	*Rattus norwegicus*	1E-19
TR11634|c0_g2	7.10	4.82E-06	GAB	Alpha-N-acetylgalactosaminidase	*Gallus gallus*	3E-35
TR87818|c0_g1	7.10	4.16E-10	IL6	Interleukin-6	*Paralichthys olivaceus*	7E-70
TR42629|c0_g1	7.07	5.14E-06	INHBB	Inhibin_beta_B_chain	*Gallus gallus*	1E-77
TR13042|c4_g6	7.04	8.57E-09	DPYD	Dihydropyrimidine_dehydrogenase_[.DP(+)]	*Danio rerio*	5E-38
TR14183|c0_g1	6.91	4.16E-10	RET7	Retinoid-binding_protein_7	*Mus musculus*	5E-54
TR41627|c0_g1	6.90	9.11E-11	TLR5	Toll-like_receptor_5	*Homo sapiens*	6E-92
TR13890|c0_g3	6.73	1.16E-10	IL8	interleukin-8-like	*Serola dumerili*	6E-21
TR70589|c2_g2	6.70	1.97E-04	PTGES	Prostaglandin_E_synthase	*Equus ferus*	1E-22
TR76899|c0_g1	6.63	6.22E-06	IL17F	Interleukin-17F	*Rattus norwegicus*	1E-11
TR28096|c4_g11	6.57	9.52E-06	GRM4	Metabotropic_glutamate_receptor_4	*Rattus norwegicus*	7E-63
TR41114|c0_g2	6.52	1.16E-10	C1QL3	Complement_C1q-like_protein_3	*Mus musculus*	2E-12
TR41089|c3_g7	6.52	4.31E-06	MORC2	MORC_family_CW-type_zinc_finger_protein_2	*Homo sapiens*	2E-27
TR32785|c0_g2	6.42	6.87E-07	A2GL	Leucine-rich_alpha-2-glycoprotein	*Homo sapiens*	7E-46
TR13469|c0_g1	6.33	1.47E-05	MA7D2	MAP7_domain-containing_protein_2	*Pongo sp.*	5E-08
TR47931|c1_g1	6.30	2.65E-07	MOT4	Monocarboxylate_transporter_4	*Gallus gallus*	2E-149
TR31477|c0_g1	6.28	7.76E-06	NDF4	Neurogenic_differentiation_factor_4	*Homo sapiens*	7E-68

TR74574|c0_g2	6.25	2.64E-05	RGS5	Regulator_of_G-protein_signaling_5	*Rattus norwegicus*	1E-26
TR8953|c5_g4	6.22	6.56E-08	CO5	Complement_C5	*Homo sapiens*	0
TR48860|c0_g1	6.12	3.43E-04	EMAL6	Echinoderm_microtubule-associated_protein-like_6	*Homo sapiens*	1E-98
TR45357|c0_g2	6.08	2.31E-09	TGM2	Protein-glutamine_gamma-glutamyltransferase_2	*Pagrus major*	4E-153
TR38005|c0_g2	6.07	2.99E-07	HME2A	Homeobox_protein_engrailed-2-A	*Xenopus laevis*	5E-61
TR64331|c3_g1	6	4.16E-10	DPYD	Dihydropyrimidine_dehydrogenase_[.DP(+)]	*Danio rerio*	2E-49
TR81910|c0_g1	5.89	7.57E-09	CHSP1	Calcium-regulated_heat_stable_protein_1	*Rattus norwegicus*	2E-19
TR45395|c0_g1	5.85	3.36E-05	SPIC	Transcription_factor_Spi-C	*Bos taurus*	1E-34
TR78110|c0_g1	5.83	9.67E-05	CFAB	Complement_factor_B	*Bos taurus*	3E-63
TR57748|c0_g1	5.81	1.28E-05	KC.3	Potassium_voltage-gated_channel_subfamily_A_member_3	*Mus musculus*	0
TR71513|c0_g2	5.80	7.24E-05	S6A17	Sodium-dependent_neutral_amino_acid_transporter_SLC6A17	*Mus musculus*	1E-65
TR42615|c0_g1	5.63	3.76E-05	TRI55	Tripartite_motif-containing_protein_55	*Rattus norwegicus*	2E-37
TR10806|c0_g1	5.58	2.38E-05	GRAM3	GRAM_domain-containing_protein_3	*Rattus norwegicus*	5E-10
TR145009|c0_g1	5.55	8.80E-05	SIA4B	CMP-N-acetylneuraminate-beta-galactosamide-alpha-2,3-sialyltransferase_2	*Homo sapiens*	3E-118
TR78132|c0_g1	5.51	8.84E-05	FABP7	Fatty_acid-binding_protein,_brain	*Mus musculus*	9E-08
TR23250|c0_g1	5.50	2.12E-05	FXYD6	FXYD_domain-containing_ion_transport_regulator_6	*Mus musculus*	6E-22

Supplemental Table 3. Overview of the 50 most down-regulated genes at 24 hpe (sorted by logFC)

50 most downregulated genes at 24 hpe (sorted by logFC)						
gene_id	logFC_24h	adjpval_24h	Annotations	Full record name	Specie	E-value
TR41171|c0_g1	-7.56	2.02E-06	LRC3B	Leucine-rich_repeat-containing_protein_3B	*Homo sapiens*	9E-65
TR70719|c4_g4	-7.46	4.49E-07	NSMA2	Sphingomyelin_phosphodiesterase_3	*Mus musculus*	3E-85
TR45504|c0_g2	-7.30	1.59E-07	S43A3	Solute_carrier_family_43_member_3	*Homo sapiens*	2E-72
TR51281|c0_g3	-7.26	1.44E-06	TGM2	Protein-glutamine_gamma-glutamyltransferase_2	*Gallus gallus*	8E-80
TR53083|c0_g1	-7.17	5.90E-06	MRC1	Macrophage_mannose_receptor_1	*Mus musculus*	2E-39
TR15942|c0_g2	-7.09	5.77E-07	DMBT1	Deleted_in_malignant_brain_tumors_1_protein	*Homo sapiens*	1E-73
TR80906|c0_g1	-7	1.21E-05	IL17F	Interleukin-17F	*Rattus norwegicus*	4E-11
TR31532|c0_g1	-6.74	1.13E-05	KCJ12	ATP-sensitive_inward_rectifier_potassium_channel_12	*Gallus gallus*	0
TR68049|c0_g1	-6.56	9.71E-05	CNKR2	Connector_enhancer_of_kinase_suppressor_of_ras_2	*Homo sapiens*	0
TR3259|c0_g1	-6.53	3.10E-06	TNR3	Tumor_necrosis_factor_receptor_superfamily_member_3	*Homo sapiens*	8E-09
TR53083|c0_g2	-6.45	2.87E-05	MRC1	Macrophage_mannose_receptor_1	*Mus musculus*	1E-39
TR65297|c3_g1	-6.44	1.44E-06	TMM88	Transmembrane_protein_88	*Mus musculus*	2E-12
TR69288|c0_g1	-6.36	9.32E-07	TNR11	Tumor_necrosis_factor_receptor_superfamily_member_11A	*Mus musculus*	9E-13
TR28894|c2_g1	-6.28	5.44E-06	COR2A	Coronin-2A	*Homo sapiens*	0
TR2247|c0_g1	-6.23	2.19E-06	RLBP1	Retinaldehyde-binding_protein_1	*Bos taurus*	2E-158
TR144820|c0_g2	-6.10	2.97E-05	PRDM1	PR_domain_zinc_finger_protein_1	*Mus musculus*	2E-26
TR6306|c0_g2	-6.03	1.49E-03	EVA1A	Protein_eva-1_homolog_A	*Danio rerio*	5E-37
TR87113|c1_g2	-6.01	9.36E-04	CEBPD	CCAAT/enhancer-binding_protein_delta	*Homo sapiens*	2E-56
TR53083|c1_g5	-5.90	1.16E-10	MRC1	Macrophage_mannose_receptor_1	*Mus musculus*	0
TR80012|c0_g1	-5.87	5.45E-08	S12A4	Solute_carrier_family_12_member_4	*Homo sapiens*	0
TR49079|c0_g3	-5.75	3.21E-06	MITF	Microphthalmia-associated_transcription_factor	*Homo sapiens*	1E-67
TR65368|c1_g21	-5.73	6.74E-05	ANKR1	Ankyrin_repeat_domain-containing_protein_1	*Gallus gallus*	4E-49
TR26688|c3_g1	-5.72	1.34E-02	ENOG	Gamma-enolase	*Rattus norwegicus*	2E-133
TR40542|c0_g1	-5.68	7.31E-07	HECA2	HEPACAM_family_member_2	*Homo sapiens*	1E-06
TR37184|c1_g3	-5.62	2.73E-04	FDXA1	Ferredoxin-fold_anticodon-binding_domain-containing_protein_1_homolog	*Mus musculus*	2E-27
TR8782|c0_g1	-5.56	3.30E-05	EPD2	Ependymin-2	*Carassius auratus*	3E-22
TR68285|c0_g1	-5.50	5.35E-05	DTBP1	Dysbindin	*Gallus gallus*	6E-31
TR880|c0_g2	-5.48	2.73E-05	MAP6	Microtubule-associated_protein_6_homolog	*Gallus gallus*	1E-31
TR71817|c0_g1	-5.44	2.54E-05	TGFA1	Transforming_growth_factor-beta_receptor-associated_protein_1	*Mus musculus*	1E-123
TR101|c0_g1	-5.41	1.04E-06	VAT1	Synaptic_vesicle_membrane_protein_VAT-1_homolog	*Danio rerio*	0
TR34008|c0_g1	-5.33	2.52E-07	SEM4G	Semaphorin-4G	*Homo sapiens*	0
TR44282|c0_g1	-5.32	3.20E-06	PHEX	Metalloendopeptidase_homolog_PEX	*Mus musculus*	0

TR43339|c0_g1	-5.31	1.36E-05	NFIL3	Nuclear_factor_interleukin-3-regulated_protein	*Rattus norwegicus*	2E-17
TR62246|c0_g1	-5.25	2.81E-05	JIP1	C-Jun-amino-terminal_kinase-interacting_protein_1	*Homo sapiens*	2E-141
TR14442|c0_g1	-5.21	6.43E-05	TLR13	Toll-like_receptor_13	*Mus musculus*	3E-66
TR54313|c0_g2	-5.19	5.12E-06	FABPL	Fatty_acid-binding_protein,_liver	*Ginglymostoma cirratum*	2E-14
TR5820|c0_g1	-5.14	1.30E-04	SREC	Scavenger_receptor_class_F_member_1	*Homo sapiens*	9E-37
TR85479|c0_g2	-5.14	7.80E-05	S29A1	Equilibrative_nucleoside_transporter_1	*Rattus norwegicus*	3E-42
TR78540|c0_g2	-5.11	9.48E-05	PERF	Perforin-1	*Rattus norwegicus*	8E-129
TR9527|c0_g1	-4.99	3.57E-06	NUAK1	NUAK_family_SNF1-like_kinase_1	*Homo sapiens*	0
TR65997|c0_g1	-4.98	1.54E-05	EFNB2	Ephrin-B2a	*Danio rerio*	5E-40
TR156627|c0_g3	-4.96	8.03E-03	CBP	CREB-binding_protein	*Rattus norwegicus*	1E-08
TR40644|c0_g2	-4.95	1.74E-04	RGS5	Regulator_of_G-protein_signaling_5	*Rattus norwegicus*	7E-55
TR38943|c0_g1	-4.93	3.66E-04	FGF4	Fibroblast_growth_factor_4	*Gallus gallus*	6E-09
TR6306|c0_g5	-4.90	4.04E-05	EVA1A	Protein_eva-1_homolog_A	*Danio rerio*	4E-37
TR25290|c0_g4	-4.90	1.61E-04	KCNH6	Potassium_voltage-gated_channel_subfamily_H_member_6	*Rattus norwegicus*	2E-83
TR12896|c0_g2	-4.85	1.08E-03	AMPN	Aminopeptidase_N_{ECO:0000312|EMBL:ACZ95799.1}	*Gallus gallus*	4E-141
TR1713|c0_g2	-4.84	2.41E-05	APOA4	Apolipoprotein_A-IV	*Papio anubis*	2E-28
TR56616|c0_g1	-4.72	2.88E-04	TGM2	Protein-glutamine_gamma-glutamyltransferase_2	*Homo sapiens*	3E-145

Supplemental Table 4. Overview of the 50 most up-regulated genes at 6 hpe (sorted by logFC)

50 most upregulated genes at 6 hpe (sorted by logFC)						
gene_id	logFC_6h	adjpval_6h	Annotations	Full record name	Specie	E-value
TR76899|c0_g1	9.37	7.35E-06	IL17F	Interleukin-17F	*Rattus norwegicus*	1E-11
TR26702|c0_g1	9.05	8.57E-06	N42L1	NEDD4-binding_protein_2-like_1	*Mus musculus*	3E-07
TR20674|c0_g2	7.73	4.87E-06	HYAS1	Hyaluronan_synthase_1	*Xenopus laevis*	0
TR45366|c0_g1	7.13	2.38E-05	ZN648	Zinc_finger_protein_648	*Homo sapiens*	7E-112
TR78231|c0_g1	6.87	1.18E-03	GDF15	Growth/differentiation_factor_15	*Mus musculus*	2E-24
TR32785|c0_g2	6.74	7.21E-05	A2GL	Leucine-rich_alpha-2-glycoprotein	*Homo sapiens*	7E-46
TR14360|c3_g2	6.69	7.93E-09	IL1B	Interleukin-1_beta	*Oncorhynchus mykiss*	9E-73
TR87818|c0_g1	6.51	1.62E-07	IL6	Interleukin-6	*Paralichthys olivaceus*	7E-70
TR13890|c0_g3	6.39	9.31E-10	IL8	interleukin-8-like	*Serola dumerili*	6E-21
TR81776|c4_g4	6.17	2.90E-09	CC chemokine 20	C-C motif chemokine 20 precursor	*Anoplopoma fimbria*	8E-45
TR76870|c0_g2	5.85	4.53E-07	EPGN	Epigen	*Gallus gallus*	1E-09
TR45395|c0_g1	5.60	4.71E-04	SPIC	Transcription_factor_Spi-C	*Bos taurus*	1E-34
TR19933|c0_g1	5.47	1.69E-08	GL.	Glutamine_synthetase	*Acomys cahirinus*	4E-109
TR67971|c1_g1	5.46	5.24E-05	LPP	Lipoma-preferred_partner_homolog	*Gallus gallus*	1E-28
TR74252|c0_g1	5.40	9.82E-06	CFAH	Complement_factor_H	*Homo sapiens*	2E-34
TR49945|c0_g1	5.26	7.93E-09	GL.	Glutamine_synthetase,_mitochondrial	*Squalus acanthias*	6E-22
TR87414|c0_g1	5.19	7.93E-09	GL.	Glutamine_synthetase	*Canis familiaris*	2E-147
TR44307|c0_g2	5.17	2.60E-04	VAX2B	Ventral_anterior_homeobox_2b	*Xenopus laevis*	5E-63
TR27410|c1_g1	5.15	8.04E-07	NOXO1	.DPH_oxidase_organizer_1	*Mus musculus*	4E-59
TR81910|c0_g1	5.13	1.67E-06	CHSP1	Calcium-regulated_heat_stable_protein_1	*Rattus norwegicus*	2E-19
TR66238|c0_g2	5.08	1.50E-04	CN159	UPF0317_protein_C14orf159_homolog,_mitochondrial	*Mus musculus*	2E-15
TR87025|c0_g2	5	1E-09	Uncharacterized protein	PREDICTED: uncharacterized protein LOC108879818	*Lates calcarifer*	5E-73
TR68048|c0_g1	5	2.42E-04	GRAM3	GRAM_domain-containing_protein_3	*Rattus norwegicus*	2E-10
TR14183|c0_g1	4.94	6.67E-08	RET7	Retinoid-binding_protein_7	*Mus musculus*	5E-54
TR11634|c0_g2	4.91	4E-06	.GAB	Alpha-N-acetylgalactosaminidase	*Gallus gallus*	3E-35
TR61339|c0_g1	4.85	6.62E-04	ISL2A	Insulin_gene_enhancer_protein_isl-2a	*Danio rerio*	0
TR80347|c0_g1	4.70	1.70E-07	HEMO	Hemopexin_{ECO:0000250|UniProtKB:P20058}	*Danio rerio*	9E-46
TR34004|c3_g1	4.50	7.93E-09	TNIP2	TNFAIP3-interacting_protein_2	*Mus musculus*	5E-20
TR78134|c0_g1	4.46	2.34E-03	SSR2	Somatostatin_receptor_type_2	*Sus scrofa*	3E-118
TR44039|c0_g1	4.41	4.89E-03	ETV6	Transcription_factor_ETV6	*Homo sapiens*	5E-20
TR86119|c0_g1	4.40	3.17E-09	TNIP2	TNFAIP3-interacting_protein_2	*Mus musculus*	2E-31
TR69814|c0_g2	4.38	2.80E-08	TNFA	Tumor_necrosis_factor	*Sparus auratus*	3E-114
TR45367|c1_g2	4.35	3.78E-03	HPT	Haptoglobin	*Rattus norwegicus*	1E-19
TR31519|c0_g1	4.31	8.49E-10	NR4A1	Nuclear_receptor_subfamily_4_group_A_member_1	*Xenopus laevis*	6E-52

TR45033|c0_g1	4.30	2.83E-03	SSR2	Somatostatin_receptor_type_2	*Homo sapiens*	1E-62
TR27348|c0_g2	4.30	2.48E-02	IKBA	NF-kappa-B_inhibitor_alpha	*Sus scrofa*	5E-06
TR41627|c0_g1	4.28	2.90E-09	TLR5	Toll-like_receptor_5	*Homo sapiens*	6E-92
TR69283|c1_g1	4.27	2.90E-09	Hypothetical protein	hypothetical protein Z043_122910	*Scleropages formosus*	9E-05
TR57748|c0_g1	4.24	4.95E-04	KC.3	Potassium_voltage-gated_channel_subfamily_A_member_3	*Mus musculus*	0
TR31477|c0_g1	4.20	6.99E-04	NDF4	Neurogenic_differentiation_factor_4	*Homo sapiens*	7E-68
TR45357|c0_g2	4.18	3.24E-07	TGM2	Protein-glutamine_gamma-glutamyltransferase_2	*Pagrus major*	4E-153
TR57132|c0_g1	4.17	9.26E-03	LORF2	LINE-1_retrotransposable_element_ORF2_protein	*Homo sapiens*	3E-20
TR80347|c0_g2	4.12	1.69E-03	HEMO	Hemopexin_{ECO:0000250|UniProtKB:P20058}	*Danio rerio*	3E-14
TR80849|c0_g2	4.11	1.47E-03	SAA1	Serum_amyloid_A-1_protein	*Mus musculus*	3E-11
TR23312|c0_g1	4.10	1.10E-08	ADTRP	Androgen-dependent_TFPI-regulating_protein	*Homo sapiens*	4E-06
TR69281|c3_g2	4.08	4.79E-03	MTSS1	Metastasis_suppressor_protein_1	*Homo sapiens*	2E-31
TR64314|c0_g1	4.05	5.05E-05	S12A2	Solute_carrier_family_12_member_2	*Mus musculus*	1E-06
TR61516|c0_g2	3.95	3.33E-03	BIRC6	Baculoviral_IAP_repeat-containing_protein_6	*Homo sapiens*	4E-125
TR45367|c1_g1	3.94	4.97E-04	HPT	Haptoglobin	*Sus scrofa*	3E-40
TR41618|c1_g3	3.94	1.11E-04	PDK2	[Pyruvate_dehydrogenase_(acetyl-transferring)]_kinase_isozyme_2,_mitochondrial	*Rattus norwegicus*	7E-09

Supplemental Table 5. Overview of the 50 most down-regulated genes at 6 hpe (sorted by logFC)

50 most downregulated genes at 6 hpe (sorted by logFC)						
gene_id	logFC_6h	adjpval_6h	Annotations	Full record name	Specie	E-value
TR145793|c0_g6	-6.30	2.63E-02	ZFX	Zinc_finger_X-chromosomal_protein	*Mus musculus*	9E-117
TR53083|c0_g2	-5.29	1.61E-03	MRC1	Macrophage_mannose_receptor_1	*Mus musculus*	1E-39
TR55536|c0_g2	-4.63	2.16E-02	CCR6	C-C_chemokine_receptor_type_6	*Homo sapiens*	2E-76
TR40542|c0_g1	-4.24	7.23E-04	HECA2	HEPACAM_family_member_2	*Homo sapiens*	1E-06
TR145793|c0_g1	-4.23	4.14E-02	ZFX	Zinc_finger_X-chromosomal_protein	*Mus musculus*	1E-116
TR85757|c0_g4	-3.68	6.06E-02	ZBT37	Zinc_finger_and_BTB_domain-containing_protein_37	*Homo sapiens*	2E-79
TR62246|c0_g1	-3.61	1.49E-03	JIP1	C-Jun-amino-terminal_kinase-interacting_protein_1	*Homo sapiens*	2E-141
TR150022|c0_g1	-3.56	4.70E-03	LPAR4	Lysophosphatidic_acid_receptor_4	*Mus musculus*	5E-17
TR53083|c0_g1	-3.52	1.60E-03	MRC1	Macrophage_mannose_receptor_1	*Mus musculus*	2E-39
TR71817|c0_g1	-3.48	2.46E-03	TGFA1	Transforming_growth_factor-beta_receptor-associated_protein_1	*Mus musculus*	1E-123
TR40529|c0_g3	-3.26	1.98E-02	JDP2	Jun_dimerization_protein_2	*Rattus norwegicus*	6E-57
TR57429|c0_g9	-3.24	9.04E-03	ABI3	ABI_gene_family_member_3	*Homo sapiens*	5E-10
TR16539|c0_g2	-3.17	5.35E-02	TSN8	Tetraspanin-8	*Bos taurus*	2E-07
TR36086|c0_g2	-3.08	3E-02	CXCR5	C-X-C_chemokine_receptor_type_5	*Rattus norwegicus*	1E-36
TR67966|c4_g1	-3.04	1.09E-01	UB2G2	Ubiquitin-conjugating_enzyme_E2_G2	*Pongo sp.*	8E-10
TR33782|c1_g4	-3.04	3.81E-06	MMP14	Matrix_metalloproteinase-14	*Mus musculus*	5E-138
TR67966|c4_g4	-3.02	6.91E-02	UB2G2	Ubiquitin-conjugating_enzyme_E2_G2	*Pongo sp.*	7E-10
TR633|c0_g3	-3.02	1.51E-01	ADAT2	tR.-specific_adenosine_deaminase_2	*Danio rerio*	1E-71
TR44789|c0_g1	-3	1.69E-01	KAD2	Adenylate_kinase_2,_mitochondrial_{ECO:0000255|HAMAP-Rule:MF_03168}	*Salmo salar*	2E-15
TR34008|c0_g1	-2.93	3.81E-06	SEM4G	Semaphorin-4G	*Homo sapiens*	0
TR69449|c4_g10	-2.92	1.96E-02	RTXE	Probable_R.-directed_D._polymerase_from_transposon_X-element	*Drosophila melongaster*	2E-13
TR70501|c4_g2	-2.91	7.79E-03	ABI2	Abl_interactor_2	*Mus musculus*	2E-101
TR65297|c3_g1	-2.88	5.13E-04	TMM88	Transmembrane_protein_88	*Mus musculus*	2E-12
TR25793|c0_g1	-2.85	2.12E-02	FHOD3	FH1/FH2_domain-containing_protein_3	*Mus musculus*	8E-22
TR2196|c0_g2	-2.83	3.60E-02	WDR18	WD_repeat-containing_protein_18	*Danio rerio*	1E-76
TR35910|c0_g2	-2.77	1.18E-01	ADA1D	Alpha-1D_adrenergic_receptor	*Sus scrofa*	5E-36
TR49070|c0_g1	-2.75	5.86E-05	SPTB2	Spectrin_beta_chain,_non-erythrocytic_1	*Mus musculus*	3E-13
TR84473|c0_g1	-2.74	1.25E-02	TLR2	Toll-like_receptor_2	*Cricetulus griseus*	3E-15
TR87042|c0_g6	-2.73	1.65E-01	ADCK1	Uncharacterized_aarF_domain-containing_protein_kinase_1	*Homo sapiens*	8E-09
TR27448|c0_g3	-2.72	3.35E-01	SC6A8	Sodium-_and_chloride-dependent_creatine_transporter_1	*Rattus norwegicus*	0
TR15942|c0_g2	-2.70	4.75E-06	DMBT1	Deleted_in_malignant_brain_tumors_1_protein	*Homo sapiens*	1E-73
TR25104|c0_g5	-2.68	1.95E-01	FND3A	Fibronectin_type-III_domain-containing_protein_3A	*Homo sapiens*	3E-13
TR85479|c0_g2	-2.66	4.34E-03	S29A1	Equilibrative_nucleoside_transporter_1	*Rattus norwegicus*	3E-42
TR84456|c0_g3	-2.64	1.85E-01	PSPC1	Paraspeckle_component_1	*Danio rerio*	2E-134

TR65271|c0_g1	-2.63	1.26E-02	VINC	Vinculin	*Xenopus laevis*	5E-15
TR60099|c0_g2	-2.63	1.16E-01	TRIM3	Tripartite_motif-containing_protein_3	*Mus musculus*	2E-94
TR24328|c0_g1	-2.61	2.38E-03	TM158	Transmembrane_protein_158	*Mus musculus*	7E-63
TR85510|c0_g3	-2.60	7.17E-03	F264	6-phosphofructo-2-kinase/fructose-2,6-bisphosphatase_4	*Mus musculus*	6E-40
TR74312|c1_g7	-2.58	3.17E-03	PI42A	Phosphatidylinositol_5-phosphate_4-kinase_type-2_alpha	*Gallus gallus*	6E-111
TR43358|c0_g1	-2.55	5.16E-03	I22R2	Interleukin-22_receptor_subunit_alpha-2	*Mus musculus*	2E-22
TR65255|c0_g11	-2.48	3.49E-03	LORF2	LINE-1_retrotransposable_element_ORF2_protein	*Mus musculus*	2E-46
TR25130|c0_g2	-2.46	1.92E-02	AQP8	Aquaporin-8	*Homo sapiens*	2E-17
TR22880|c0_g3	-2.46	1.95E-01	MED23	Mediator_of_R._polymerase_II_transcription_subunit_23	*Danio rerio*	0
TR41097|c0_g1	-2.45	2.94E-03	MAGI1	Membrane-associated_guanylate_kinase,_WW_and_PDZ_domain-containing_protein_1	*Rattus norwegicus*	6E-44
TR65980|c0_g1	-2.42	2.11E-04	FAT3	Protocadherin_Fat_3	*Rattus norwegicus*	0
TR8375|c0_g1	-2.41	9.46E-05	P2RX5	P2X_purinoceptor_5	*Rattus norwegicus*	2E-82
TR57480|c0_g1	-2.40	2.56E-05	SPTN5	Spectrin_beta_chain,_non-erythrocytic_5	*Homo sapiens*	4E-20
TR26681|c0_g5	-2.38	1.47E-01	SOS1	Son_of_sevenless_homolog_1	*Homo sapiens*	3E-158
TR28080|c0_g1	-2.38	2.15E-03	SVEP1	Sushi,_von_Willebrand_factor_type_A,_EGF_and_pentraxin_domain-containing_protein_1	*Homo sapiens*	0
TR40831|c1_g1	-2.36	1.58E-01	CEGT	Ceramide_glucosyltransferase	*Mus musculus*	3E-69
TR59866|c0_g1	-2.36	4.71E-08	P5CS	Delta-1-pyrroline-5-carboxylate_synthase	*Pongo sp.*	1E-165

Supplemental Table 6. Overview of verified lumpfish genes in the complement cascade

			Top BLAST hit			
Gene-ID	Name	KEGG orthology ID	Description	E-value	Species	Accession number
Complement components						
TR16469|c0_g4	C1R	K01330	complement component C1q receptor	0	*Monopterus albus*	XP_020479096
TR40321|c0_g2	C1R/1S	K01331	Ca2+-dep.complex C1R/C1S subunit	5.14E-152	*Perca flavescens*	ABU63968
TR74292|c0_g1	C1S	K01331	complement component 1s	2.38E-162	*Oplegnathus fasciatus*	AIZ96980
TR74819|c0_g1	C2	K01332	complement component 2	1.20E-143	*Oplegnathus fasciatus*	AIN76765
TR104686|c0_g1<br>TR63519|c0_g1	C3<br>C3	K03990<br>K03990	PRED: complement C3-like<br>PRED: complement C3-like	7E-54<br>8E-57	*Lates calcarifer*<br>*Lates calcarifer*	XP_018528015<br>XP_018528013
TR56223|c0_g1<br>TR1140|c0_g2<br>TR78116|c0_g1	C3<br>C3<br>C3	K03990<br>K03990<br>K03990	complement C3-like<br>complement C3-like<br>complement C3-like	2.16E-165<br>2E-144<br>0	*Acanthochromis polyacanthus*<br>*Oryzias latipes*<br>*Stegastes partitus*	XP_022077167<br>XP_020555289<br>XP_008298001
TR77762|c0_g1	C3	K03990	PRED: complement component C3	6E-46	*Paralichthys olivaceus*	XP_019939804
TR88215|c0_g1	C3	K03990	complement component C3	8E-47	*Acanthochromis polyacanthus*	XP_022064368
TR64828|c1_g1	C4	K03989	complement component 4	0	*Oplegnathus fasciatus*	AIN76766
TR8953|c5_g4	C5	K03994	PRED: complement C5	0	*Lates calcarifer*	XP_018549132
TR49109|c2_g3<br>TR67474|c0_g1<br>TR62911|c0_g1	C5AR1<br>C5AR1<br>C5AR1	K04010<br>K04010<br>K04010	C3a anaphylatoxin chemotactic receptor<br>C3a anaphylatoxin chemotactic receptor<br>C3a anaphylatoxin chemotactic receptor	3.36E-156<br>1.77E-131<br>0	*Larimichthys crocea*<br>*Seriola dumerili*<br>*Seriola dumerili*	XP_010733778<br>XP_022617678<br>XP_022616734
TR48847|c0_g1<br>TR48847|c0_g2<br>TR70869|c1_g1<br>TR70745|c3_g1	C5AR1<br>C5AR1<br>C5AR1<br>C5AR1	K04010<br>K04010<br>K04010<br>K04010	chemokine-like receptor 1<br>chemokine-like receptor 1<br>chemokine-like receptor 1<br>chemokine-like receptor 1	0<br>0<br>0<br>7.7E-170<br>0	*Notothenia coriiceps*<br>*Notothenia coriiceps*<br>*Acanthochromis polyacanthus*<br>*Acanthochromis polyacanthus*	XP_010785059<br>XP_010785059<br>XP_022065692<br>XP_022065692
TR121251|c0_g1<br>TR43399|c0_g1	C6<br>C6	K03995<br>K03995	complement component C6 isoform X1<br>complement component C6-like protein	2.08E-30<br>3.4E-155	*Labrus bergylta*<br>*Siniperca chuatsi*	XP_020491684<br>AKA66307
TR15677|c0_g1<br>TR69965|c1_g1	C6<br>C6	K03995<br>K03995	complement component C6<br>complement component C6	2.18E-44<br>1.16E-94	*Kryptolebias marmoratus*<br>*Notothenia coriiceps*	XP_017259971<br>XP_010771775
TR77069|c0_g1	C7	K03996	complement component C7	1.8E-100	*Oplegnathus fasciatus*	AFZ93893
TR40591|c0_g1	C7	K03996	PRED: complement component C7 X2	2.13E-31	*Larimichthys crocea*	XP_019120079
TR62647|c0_g1	C7	K03996	complement component C7-2	3.59E-67	*Miichthys miiuy*	AKM12676
TR37853|c0_g1<br>TR94503|c0_g1	C7<br>C7	K03996<br>K03996	PRED: complement component C7-like<br>PRED: complement component C7-like	5.71E-124<br>5.71E-124	*Notothenia coriiceps*<br>*Notothenia coriiceps*	XP_010792065<br>XP_010792065
TR122188|c0_g1<br>TR53398|c0_g1	C7<br>C7	K03996<br>K03996	complement component C7-like<br>complement component C7-like	9.5E-57<br>4.1E-153	*Monopterus albus*<br>*Monopterus albus*	XP_020479067<br>XP_020479067

TR126963|c0_g1	C7	K03996	complement component C7-like	1.17E-41	*Seriola dumerili*	XP_022600176
TR30315|c0_g1	C8A	K03997	complement component 8 alpha	0	*Siniperca chuatsi*	AKA66305
TR27293|c0_g1	C8B	K03998	complement component 8 beta	0	*Oplegnathus fasciatus*	AFZ93889
TR122188|c0_g1<br>TR53398|c0_g1<br>TR126963|c0_g1	C9<br>C9<br>C9	K04000<br>K04000<br>K04000	complement component 9<br>complement component 9<br>complement component 9	3.3E-06<br>1.01E-57<br>2.22E-60	*Oryzias latipes*<br>*Paralichthys olivaceus*	XP_004074560<br>BAA86878<br>BAA86878
TR78110|c0_g1	CFB	K01335	PRED: complement factor B-like	0	*Lates calcarifer*	XP_018547243
TR5891|c0_g1	CFD	K01334	PRED: complement factor D-like	1.54E-162	*Lates calcarifer*	XP_018541031
TR62909|c0_g1<br>TR74252|c0_g1	CFH<br>CFH	K04004<br>K04004	PRED: complement factor H-like<br>PRED: complement factor H-like	0<br>6e-122	*Hippocampus comes*<br>*Lates calcarifer*	XP_019740144<br>XP_01852636
TR84448|c0_g1	CFI	K01333	complement factor I	0	*Labrus bergylta*	XP_020514187
TR62930|c0_g1	CFP	K15412	properdin	0	*Acanthochromis polyacanthus*	XP_022073264
TR63188|c0_g1	CLU	K17252	clusterin	1.08E-168	*Monopterus albus*	XP_020459633
TR13054|c1_g2<br>TR64830|c2_g2	CR3<br>CR3	K06461<br>K06461	Integrin alpha-M (ITAM)<br>Integrin alpha-M (ITAM)	0<br>0	*Dicentrarchus labrax*<br>*Dicentrarchus labrax*	CBN81367<br>CBN81367
TR100|c0_g1<br>TR15999|c3_g3	ITGB2<br>ITGB2	K06464<br>K06464	integrin beta-2-like<br>integrin beta-2-like	6.70<br>6.70	*Hippocampus comes*<br>*Hippocampus comes*	XP_019731231<br>XP_019731231
TR13103|c3_g7	ITGB2	K06464	integrin beta-2-like	0	*Monopterus albus*	XP_020462274
TR1125|c1_g2	MASP1	K03992	Mannan-binding lectin serine protease 1	0	*Larimichthys crocea*	KKF27967

Supplemental Table 7. Overview of the species included in the phylogenetic analyses

Species	Authorities	Common name	Teleost order (Nelson et al 2016)	Teleost order (Betancur-R et al.2013)	Family[1]
Danio rerio	Hamilton, 1822	zebrafish	*Cypriniformes*	*Cypriniformes*	*Cyprinidae*
Takifugu rubripes	Temminck & Schlegel, 1850	fugu	*Tetraodontiformes*	*Tetraodontiformes*	*Tetraodontidae*
Gasterosteus aculeatus	Linnaeus, 1758	stickleback	*Gasterosteiformes*	*Perciformes*	*Gasterosteidae*
Oryzias latipes	Temminck & Schlegel, 1850	medaka	*Beloniformes*	*Beloniformes*	*Adrianichthyidae*
Gadus morhua	Linnaeus, 1758	cod	*Gadiformes*	*Gadiformes*	*Gadidae*
Cyclopterus lumpus	Linnaeus, 1758	lumpfish	*Scorpaniformes*	*Perciformes*	*Cyclopteridae*
Miichthys miiuy	Basilewsky, 1855	croaker	*Perciformes*	*Percomorpharia* *	*Sciaenidae*
Ictalurus punctatus	Rafinesque, 1818	catfish	*Siluriformes*	*Siluriformes*	*Ictaluridae*
Plecoglossus altivelis altivelis	Temminck & Schlegel, 1846	ayu sweetfish	*Osmeriformes*	*Osmeriformes*	*Plecoglossidae*
Oncorhynchus mykiss	Walbaum, 1792	rainbow trout	*Salmoniformes*	*Salmoniformes*	*Salmonidae*
Coregonus maraena	Bloch, 1779	mareana whitefish	*Salmoniformes*	*Salmoniformes*	*Salmonidae*
Sparus aurata	Linnaeus, 1758	gilthead seabream	*Perciformes*	*Spariformes*	*Sparidae*
Scophthalmus maximus	Linnaeus, 1758	turbot	*Pleuronectiformes*	*Pleuronectiformes*	*Scophtalmidae*
Paralichthys olivaceus	Temminck & Schlegel, 1846	bastard halibut	*Pleuronectiformes*	*Pleuronectiformes*	*Paralichthyidae*
Oplegnathus fasciatus	Temminck & Schlegel, 1844	barred knifejaw	*Perciformes*	*Percomorpharia* *	*Oplegnathidae*
Cirrhinus mrigala	Hamilton, 1822	mrigal carp	*Cypriniformes*	*Cypriniformes*	*Cyprinidae*
Carassius auratus	Linnaeus, 1758	goldfish	*Cypriniformes*	*Cypriniformes*	*Cyprinidae*
Ctenopharyngodon idella	Valenciennes, 1844	grass carp	*Cypriniformes*	*Cypriniformes*	*Cyprinidae*
Cyprinus carpio	*Linnaeus, 1758*	common carp	*Cypriniformes*	*Cypriniformes*	*Cyprinidae*
Megalobrama amblycephala	Yih, 1955	wuchang bream	*Cypriniformes*	*Cypriniformes*	*Cyprinidae*
Salmo salar	Linnaeus, 1758	Atlantic salmon	*Salmoniformes*	*Salmoniformes*	*Salmonidae*
Larimichthys crocea	Richardson, 1846	large yellow croaker	*Perciformes*	*Percomorpharia* *	*Sciaenidae*
Epinephelus coioides	Hamilton, 1822	orange-spotted grouper	*Perciformes*	*Perciformes*	*Serranidae*
Carassius gibelio	Bloch, 1782	prussian carp	*Cypriniformes*	*Cypriniformes*	*Cyprinidae*
Gobiocypris rarus	Ye & Fu, 1983	N.A.	*Cypriniformes*	*Cypriniformes*	*Cyprinidae*
Squaliobarbus curriculus	b	barbel chub	*Cypriniformes*	*Cypriniformes*	*Cyprinidae*
Cynoglossus semilaevis	Günther, 1873	tounge sole	*Pleuronectiformes*	*Pleuronectiformes*	*Cynoglossidae*
Fundulus heteroclitus	Linnaeus, 1766	mummichug	*Cyprinodontiformes*	*Cyprinodontiformes*	*Fundulidae*
Trematomus bernacchii	Boulenger, 1902	emerald rockcod	*Perciformes*	*Perciformes*	*Nototheniidae*
Chionodraco hamatus	Lönnberg, 1905	crocodile icefishes	*Perciformes*	*Perciformes*	*Channichthyidae*

Nothobranchius furzeri	Jubb, 1971	turquoise killifish	*Cyprinodontiformes*	Not examined	*Notobranchiidae*
Nothobranchius rachovii	Ahl, 1926	bluefin notho	*Cyprinodontiformes*	Not examined	*Notobranchiidae*
Nothobranchius kadleci	Richard, 2010	N.A.	*Cyprinodontiformes*	Not examined	*Notobranchiidae*
Labeo rohita	Hamilton, 1822	roho labeo	*Cypriniformes*	*Cypriniformes*	*Cyprinidae*
Tachysurus fulvidraco	Richardson, 1846	yellow catfish	*Siluriformes*	*Siluriformes*	*Bagridae*
Tetraodon nigroviridis	Marion de Procé, 1822	spotted green pufferfish	*Tetraodontiformes*	*Tetraodontiformes*	*Tetraodontidae*
Gymnocypris przewalskii	Kessler, 1876	N.A.	*Cypriniformes*	*Cypriniformes*	*Cyprinidae*
Scleropages formosus	Müller & Schlegel, 1840	asian arowana	*Osteoglossiformes*	*Osteoglossiformes*	*Osteoglossidae*
Siniperca chuatsi	Basilewsky, 1855	mandarin fish	*Perciformes*	*Percomorpharia* *	*Perchichtyidae*
Seriola lalandi	Valenciennes,1833	yellowtail amberjack	*Perciformes*	*Carangiformes*	*Carangidae*
Lates calcarifer	Bloh, 1790	barramundi	*Perciformes*	*Carangimorpharia**	*Latidae*
Catla catla	Hamilton, 1822	catla	*Cypriniformes*	*Cypriniformes*	*Cyprinidae*
Clarias batrachus	Linnaeus, 1758	philippine catfish	*Siluriformes*	*Siluriformes*	*Clariidae*
Oreochromis niloticus	Linnaeus, 1758	nile tilapia	*Perciformes*	*Cichliformes*	*Cichlidae*
Labrus bergylta	Ascanius, 1767	ballan wrasse	*Perciformes*	*Labriformes*	*Labridae*
Stegastes partitus	Poey, 1868	bicolor damselfish	*Perciformes*	*Ovalentariae**	*Pomacentridae*
Maylandia zebra	Boulenger, 1899	zebra mbuna	*Perciformes*	*Cichliformes*	*Cichlidae*
Callorhinchus milii	Bory de Saint-Vincet, 1823	ghost shark	*Chimaeriformes*	Not examined	*Callorhichidae*

* incertae sedis. N.A. = non applicable

References:

Nelson, J. S., Grande, T. C. & Wilson, M. V. H. *Fishes of the world, 5th Edition*. (2016).
Betancur, R. R. *et al.* The tree of life and a new classification of bony fishes. *PLoS Curr* doi:10.1371/current.tol.53ba26640df0ccaee75bb165c8c26288 (2013)

Supplemental Table 8: Accession numbers of genes in the phylogenetic analyses of TLRs

Abbrevation	Species	Acc. No.
**TLR1**		
TLR1	*Coregonus maraena*	CEF90214.1
TLR1	*Ctenopharyngodon idella*	ACT68332.1
TLR1	*Cyprinus carpio*	BAU98379.1
TLR1	*Epinephelus coioides*	AEB32452.1
TLR1	*Gymnocypris przewalskii*	ANQ46688.1
TLR1	*Homo sapiens*	Q15399
TLR1	*Larimichthys crocea*	AHB51065.1
TLR1	*Megalobrama amblycephala*	APT35500.1
TLR1	*Miichthys miiuy*	AKJ66261.1
TLR1	*Mus musculus*	Q9EPQ1
TLR1	*Oncorhynchus mykiss*	ACV92063.1
TLR1	*Oryzias latipes*	XP_011478513.1
TLR1	*Paralichthys olivaceus*	AFW04264.1
TLR1	*Tachysurus fulvidraco*	ANA09008.1
TLR1	*Takifugu rubripes*	AAW69368.1
TLR1	*Tetraodon nigroviridis*	ABO15772.1
**TLR2**		
TLR2	*Carassius gibelio*	AGR53440.1
TLR2	*Chionodraco hamatus*	ACT64127.1
TLR2	*Cirrhinus mrigala*	AHI59129.1
TLR2	*Ctenopharyngodon idella*	ACT68333.1
TLR2	*Cyprinus carpio*	BAU98381.1
TLR2	*Epinephelus coioides*	AEB32453.1
TLR2	*Homo sapiens*	O60603
TLR2	*Ictalurus punctatus*	ABD17347.1
TLR2	*Labeo rohita*	ADQ74644.1
TLR2	*Larimichthys crocea*	AJP16420.1
TLR2	*Latescalcarifer*	XP_18558738.1
TLR2	*Latimeria chalumnae*	ENSLACT00000017309

TLR2	*Lepisosteus oculatus*	ENSLOCT00000021874
TLR2	*Megalobrama amblycephala*	APT35501.1
TLR2	*Mus musculus*	Q9QUN7
TLR2	*Oncorhynchus mykiss*	CCK73195.1
TLR2	*Oplegnathus fasciatus*	AFZ81806.1
TLR2	*Oryzias latipes*	XP_004079640.2
TLR2	*Paralichthys olivaceus*	BAD01044.1
TLR2	*Scophthalmus maximus*	AMQ35498.1
TLR2	*Tachysurus fulvidraco*	ANA09009.1
TLR2	*Trematomus bernacchii*	ACT64128.1
TLR2.1	*Larimichthys crocea*	KKF28982.1
TLR2.2	*Larimichthys crocea*	KKF15865.1
TLR2a	*Cyprinus carpio*	ACP20793.2
TLR2b-P	*Oncorhynchus mykiss*	NP_001117891.1
TLR2c	*Cyprinus carpio*	BAU98380.1
TLR2-P	*Danio rerio*	NP_00997977.1
**TLR3**		
TLR3	*Carassius auratus*	ABC86865.1
TLR3	*Carassius gibelio*	AGR53439.1
TLR3	*Ctenopharyngodon idella*	ABI64155.1
TLR3	*Cyprinus carpio*	ABL11473.1
TLR3	*Danio rerio*	AAT37633.1
TLR3	*Gadus morhua*	ENSGMOP00000000792
TLR3	*Epinephelus coioides*	ADZ76423.1
TLR3	*Gobiocypris rarus*	ABL11471.1
TLR3	*Homo sapiens*	O15455
TLR3	*Ictalurus punctatus*	AEI59664.1
TLR3	*Megalobrama amblycephala*	ABI83673.1
TLR3	*Miichthys miiuy*	ALJ55565.1
TLR3	*Mus musculus*	Q99MB1
TLR3	*Oncorhynchus mykiss*	AAX68425.1
TLR3	*Oryzias latipes*	XP_011475331.1
TLR3	*Paralichthys olivaceus*	BAM11216.1

TLR3	*Salmo salar*	AKE14222.1
TLR3	*Scophthalmus maximus*	AHW76803.1
TLR3	*Squaliobarbus curriculus*	ALO75529.1
TLR3	*Takifugu rubripes*	AAW69373.1
TLR3.2	*Cyprinus carpio*	AHE74142.1
TLR3.2	*Larimichthys crocea*	ADR01099.1
TLR3b	*Paralichthys olivaceus*	BAD01047.1
TLR3-P	*Ictalurus punctatus*	NP_001186997.1
TLR3-P	*Larimichthys crocea*	NP_001290242.1
**TLR4**		
TLR4	*Cyprinus carpio*	BAU98382.1
TLR4	*Homo sapiens*	O00206
TLR4	*Labeo rohita*	AOM81178.1
TLR4	*Mus musculus*	Q9QUK6
TLR4.1	*Ctenopharyngodon idella*	AEQ64877.1
TLR4.2	*Ctenopharyngodon idella*	AEQ64878.1
TLR4.3	*Ctenopharyngodon idella*	AEQ64879.1
TLR4.4	*Ctenopharyngodon idella*	AEQ64880.1
TLR4a	*Megalobrama amblycephala*	ALB39038.1
TLR4a-P	*Danio rerio*	NP_001315534.1
TLR4b	*Danio rerio*	AAH68358.1
TLR4b	*Megalobrama amblycephala*	AMH41158.1
TLR4ba	*Cyprinus carpio*	AHH85806.1
TLR4ba	*Danio rerio*	NP_001124523.1
TLR4bb	*Cyprinus carpio*	AHH85807.1
TLR4bb-P	*Danio rerio*	NP_00997978.2
TLR4c	*Megalobrama amblycephala*	AKP20514.1
**TLR5**		
TLR5	*Homo sapiens*	O60602
TLR5	*Mus musculus*	Q9JLF7
TLR5a	*Ctenopharyngodon idella*	AIO11757.1
TLR5b	*Ctenopharyngodon idella*	AIO11758.1
TLR5M	*Carassius auratus*	AQX43081.1

TLR5M	*Cirrhinus mrigala*	AHI59128.1
TLR5M	*Coregonus maraena*	CEF90216.1
TLR5M	*Cyprinus carpio*	BAU98383.1
TLR5M	*Ictalurus punctatus*	AEI59669.1
TLR5M	*Megalobrama amblycephala*	APT35502.1
TLR5M	*Miichthys miiuy*	ALJ55566.1
TLR5M	*Oplegnathus fasciatus*	AQT26515.1
TLR5M	*Oryzias latipes*	XP_011490072.1
TLR5M	*Paralichthys olivaceus*	BAJ16367.1
TLR5M	*Plecoglossus altivelis altivelis*	BAI68384.1
TLR5M	*Scophthalmus maximus*	AMQ35502.1
TLR5M-P	*Danio rerio*	NP_001124067.2
TLR5M-P	*Oncorhynchus mykiss*	NP_001118216.1
TLR5S	*Miichthys miiuy*	ALJ55567.1
TLR5S	*Paralichthys olivaceus*	AEN71826.1
TLR5S	*Scophthalmus maximus*	ANS71058.1
TLR5S	*Sparus aurata*	CCP37739.1
TLR5S	*Takifugu rubripes*	AAW69378.1
TLR5S-P	*Ictalurus punctatus*	NP_001187158.1
TLR5S-P	*Oncorhynchus mykiss*	NP_001117680.1
**TLR6**		
TLR6	*Homo sapiens*	Q9Y2C9
TLR6	*Mus musculus*	Q9EPW9
**TLR7**		
TLR7	*Cyprinus carpio*	BAJ19518.1
TLR7	*Homo sapiens*	Q9NYK1
TLR7	*Gadus morhua*	ENSGMOP00000001755
TLR7	*Ictalurus punctatus*	AEI59670.1
TLR7	*Larimichthys crocea*	AGO28200.1
TLR7	*Megalobrama amblycephala*	APT35503.1
TLR7	*Miichthys miiuy*	ALJ55568.1
TLR7	*Mus musculus*	P58681
TLR7	*Oncorhynchus mykiss*	ACV41797.1

TLR7	*Oryzias latipes*	XP_0011488154.1
TLR7	*Salmo salar*	CCX35457.1
TLR7	*Scophthalmus maximus*	AMQ35499.1
**TLR8**		
TLR8	*Homo sapiens*	Q9NR97
TLR8	*Mus musculus*	P58682
TLR8	*Takifugu rubripes*	AAW69376.1
TLR8	*Gadus morhua*	ENSGMOP00000001741
TLR8	*Larimichthys crocea*	AGO28201.1
TLR8	*Miichthys miiuy*	ALJ55569.1
TLR8	*Paralichthys olivaceus*	AOS00680.1
TLR8	*Scophthalmus maximus*	AQU15238.1
TLR8a	*Cyprinus carpio*	BAU98387.1
TLR8a	*Megalobrama amblycephala*	APT35504.1
TLR8a1	*Oncorhynchus mykiss*	ACV41799.1
TLR8a1	*Coregonus maraena*	CEF90219.1
TLR8a1	*Salmo salar*	NP_001155165.1
TLR8a2	*Oncorhynchus mykiss*	ACV41798.1
TLR8a2	*Salmo salar*	CCX35458.1
TLR8b	*Cyprinus carpio*	BAU98386.1
TLR8b2	*Coregonus maraena*	CEF90223.1
**TLR9**		
TLR9	*Chionodraco hamatus*	ACT64129.1
TLR9	*Coregonus maraena*	CEF90220.1
TLR9	*Ctenopharyngodon idella*	ADB96920.1
TLR9	*Cynoglossus semilaevis*	ACL68661.1
TLR9	*Fundulus heteroclitus*	JAR68992.1
TLR9	*Gadus morhua*	ENSGMOP00000012030
TLR9	*Homo sapiens*	Q9EQU3
TLR9	*Megalobrama amblycephala*	APT35506.1
TLR9	*Miichthys miiuy*	ALJ55570.1
TLR9	*Mus musculus*	Q9NR96
TLR9	*Paralichthys olivaceus*	BAE80691.1

TLR9	*Scophthalmus maximus*	AQU15239.1
TLR9	*Takifugu rubripes*	AAW69377.1
TLR9	*Trematomus bernacchii*	ACT64130.1
TLR9a	*Epinephelus coioides*	ACV04893.1
TLR9a	*Larimichthys crocea*	ACF60624.1
TLR9a	*Sparus aurata*	AAW81697.1
TLR9b	*Epinephelus coioides*	ACV04894.1
TLR9b	*Sparus aurata*	AAW81696.1
TLR9P	*Larimichthys crocea*	ACF60625.1
TLR9-P	*Danio rerio*	NP_001124066.1
TLR9-P	*Oncorhynchus mykiss*	NP_001123463.1
TLR9-P	*Salmo salar*	NP_001117125.1
**TLR10, TLR11, TLR12**		
TLR10	*Homo sapiens*	Q9BXR5
TLR11	*Mus musculus*	Q6R5P0
TLR12	*Mus musculus*	Q6QNU9
**TLR13**		
TLR13	*Mus musculus*	Q6R5N8
TLR13	*Miichthys miiuy*	ALJ55571.1
TLR13	*Salmo salar*	NP_001133860.1
TLR13	*Oryzias latipes*	XP_004078275.1
TLR13a	*Larimichthys crocea*	KKF19122.1
TLR13b	*Larimichthys crocea*	KKF22613.1
TLR13-P	*Larimichthys crocea*	NP_001290325.1
TLR13-P	*Oreochromis niloticus*	NP_001298246.1
**TLR14**		
TLR14	*Miichthys miiuy*	ALJ55572.1
TLR14	*Paralichthys olivaceus*	BAJ78226.1
**TLR18**		
TLR18	*Danio rerio*	AAI63840.1
TLR18	*Ictalurus punctatus*	AEI59674.1
TLR18	*Ctenopharyngodon idella*	AIB55030.1
TLR18	*Megalobrama amblycephala*	APT35507.1

TLR18	*Gadus morhua*	ENSGMOP00000004019
TLR18	*Salmo salar*	CDK60413.1
TLR18a	*Cyprinus carpio*	BAU98389.1
TLR18b	*Cyprinus carpio*	BAU98388.1
**TLR19**		
TLR19	*Megalobrama amblycephala*	APT35508.1
TLR19	*Cyprinus carpio*	BAU98390.1
**TLR20**		
TLR20	*Cyprinus carpio*	AHH85805.1
TLR20	*Ctenopharyngodon idella*	AHN49762.1
TLR20	*Megalobrama amblycephala*	APT35509.1
TLR20.2	*Danio rerio*	NP_001170914.2
TLR20a	*Ictalurus punctatus*	NP_001187159.1
**TLR21**		
TLR21	*Epinephelus coioides*	ADM34974.2
TLR21	*Gadus morhua*	AFK76484.1
TLR21	*Paralichthys olivaceus*	AFW04263.1
TLR21	*Ctenopharyngodon idella*	AGM21642.1
TLR21	*Clarias batrachus*	AGM39445.1
TLR21	*Oplegnathus fasciatus*	AIT52504.1
TLR21	*Miichthys miiuy*	ALJ55573.1
TLR21	*Scophthalmus maximus*	AMQ35500.1
TLR21	*Larimichthys crocea*	AOZ21302.1
TLR21	*Cyprinus carpio*	BAU98391.1
TLR21	*Danio rerio*	CAQ13807.1
TLR21	*Ictalurus punctatus*	NP_001186994.1
TLR21/13	*Seriola lalandi*	ALI16363.1
TLR21-P	*Takifugu rubripes*	NP_001027751.1
TLR22	*Carassius auratus*	AQX43082.1
TLR22	*Catla catla*	AGW43269.2
TLR22	*Ctenopharyngodon idella*	ADX97523.2
TLR22	*Epinephelus coioides*	AGA84053.1
TLR22	*Labeo rohita*	AHV90682.1

TLR22	*Lates calcarifer*	AOV82293.1
TLR22	*Miichthys miiuy*	ALJ55574.1
TLR22	*Scophthalmus maximus*	AIC75881.1
TLR22	*Seriola lalandi*	AKN10669.1
TLR22	*Siniperca chuatsi*	AFC95889.1
TLR22	*Sparus aurata*	CDK37745.1
TLR22	*Takifugu rubripes*	AAW69372.1
**TLR22**		
TLR22a	*Cyprinus carpio*	BAU98393.1
TLR22a	*Danio rerio*	NP_001122147.2
TLR22a	*Salmo salar*	CAJ80696.1
TLR22a2	*Salmo salar*	CAR62394.1
TLR22a-P	*Oncorhynchus mykiss*	NP_001117884.1
TLR22b	*Cyprinus carpio*	BAU98395.1
TLR22b	*Danio rerio*	AAI63527.1
TLR22b	*Gadus morhua*	AFK76486.1
TLR22c	*Cyprinus carpio*	BAU98394.1
TLR22d	*Gadus morhua*	AFK76488.1
TLR22g	*Gadus morhua*	AFK76491.1
TLR22i	*Gadus morhua*	AFK76493.1
TLR22l	*Gadus morhua*	AFK76496.1
TLR22-P	*Scleropages formosus*	KPP60030.1
TLR23	*Miichthys miiuy*	ALJ55575.1
TLR23a	*Gadus morhua*	AFK76497.1
TLR23b	*Gadus morhua*	AFK76498.1
**TLR23-TLR26**		
TLR	*Nothobranchius furzeri*	SBP54052.1
TLR	*Nothobranchius kadleci*	SBP83130.1
TLR	*Nothobranchius rachovii*	SBR74107.1
TLR25	*Ictalurus punctatus*	AEI59680.1
TLR25a	*Cyprinus carpio*	BAU98397.1
TLR25b	*Cyprinus carpio*	BAU98396.1
TLR1_25	*Oryzias latipes*	XP_004083162.1

TLR26	*Ictalurus punctatus*	AEI59681.1
**TLR27**		
TLR27	*Callorhinchus milii*	XP_007893881.1
TLR27	*Latimeria chalumnae*	ENSLACP00000017183
TLR27	*Lepisosteus oculatus*	ENSLOCP00000021836
**TLR28**		
TLR2.2	*Epinephelus coioides*	AIS23533.1
TLR2.2	*Maylandia zebra*	XP_14266344.1
TLR2.2	*Oreochromis niloticus*	XP_019215654.1
TLR2.2	*Oryzias latipes*	XP_004078522.1
TLR2.2	*Stegastes partitus*	XP_008295222.1
TLR2.2	*Labrus bergylta*	XP_020495322.1
TLR2.2	*Lates calcarifer*	XP_018558738.
TLR28	*Miichthys miiuy*	AKN63433.1

Supplementary Methods for Assembly and Annotation:

We have used Trinity v2.0.6 (https://github.com/trinityrnaseq/trinityrnaseq/wiki) installed on our Linux server (CentOS release 6.9). Transcripts were assembled using all the raw reads (n=13) with additional trinity parameters, defined for library type (RF), trimmomatic quality trimming and reads normalization, resulted assembly fasta file. Assembly was subjected for removal of vector contamination using blastn. Thereafter, reference preparation, abundance estimation, generation of expression value matrices, generic contaminants removal from matrices and detection of differentially expressed genes were performed. To add functional annotations, as recommended by Trinity, we used Trinotate v2.0.2 https://trinotate.github.io/ and TransDecoder v2.0.1 (https://github.com/TransDecoder/TransDecoder/wiki). The steps performed during assembly and annotations are available in Supplementary Figure 1 given below. All the commands used for Trinity, contaminants removal, Trinotate and TransDecoder are available in Supplementary Methods for Bioinformatic scripts.

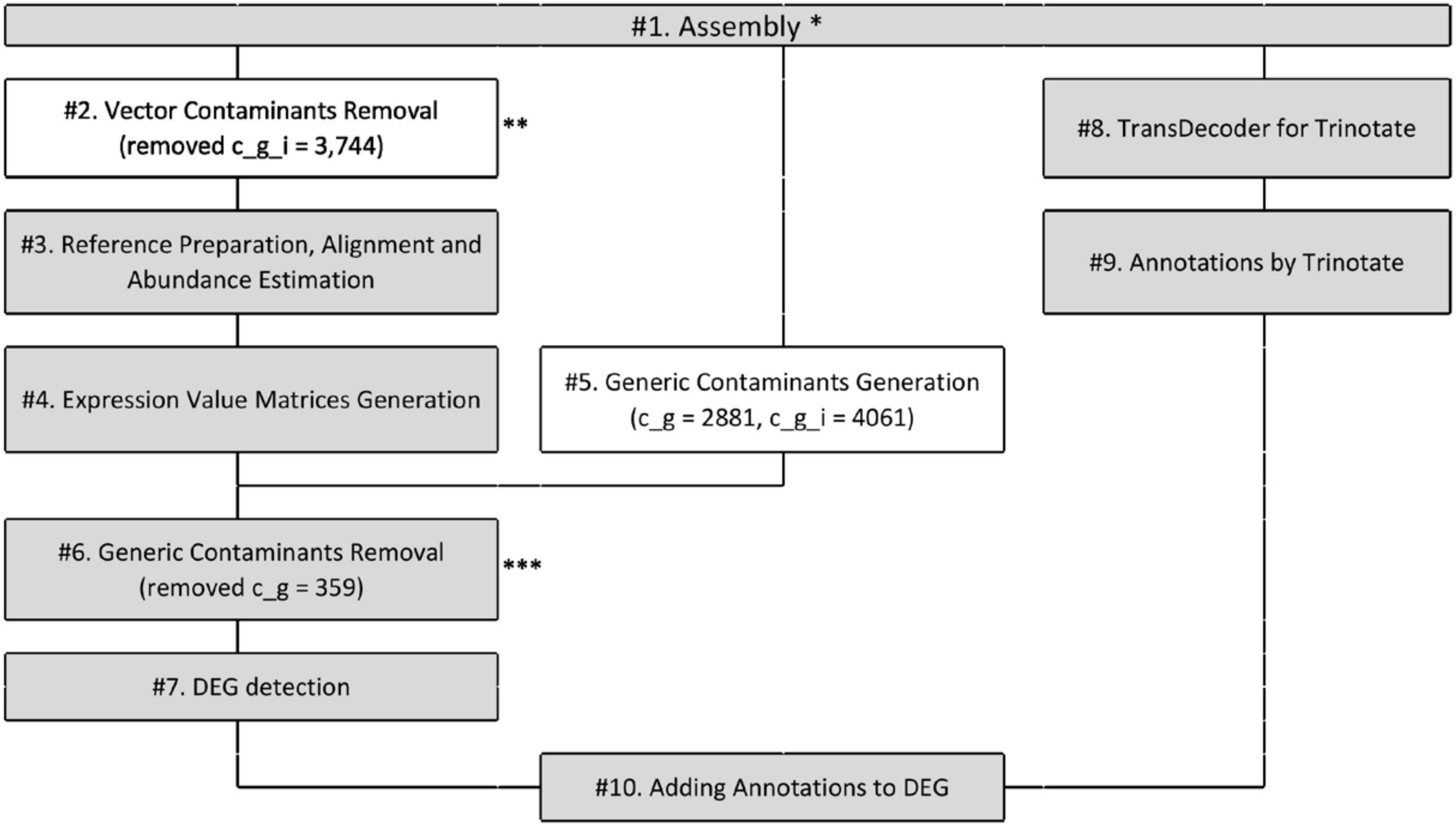

*, Raw reads .fastq.gz were trimmed during assembly with additional trinity parameters defined for trimmomatic quality trimming and resulted trimmed .fastq.gz.P.qtrim.gz files. These files were submitted to array express and available as .fastq.gz files

**, Assembly after vector contaminants removal submitted to Array Express and available as Cyclopterus_lumpus_assembly_post_trinity_Cleaned.fasta

***, Expression value matrices after removing generic contaminants submitted to Array Express as differential_expression_table.txt

c_g_i, contig_gene_isoform generated by trinity

Supplementary Figure 1. Steps performed during de novo assembly and annotations.

Supplementary Methods for Bioinformatic scripts

1. Assembly
##############
$ /trinityrnaseq-2.0.6/Trinity --seqType fq --left all_left --right all_right --output trinity_out_dir --SS_lib_type RF --trimmomatic --normalize_reads --max_memory 200G --CPU 60

2. Vector contaminants removal
###############################

1) Vector genomes seq in fasta were downloaded form NCBI:

gi|9630635|ref|NC_001915.1| Infectious pancreatic necrosis virus segment A, complete sequence

gi|9630638|ref|NC_001916.1| Infectious pancreatic necrosis virus segment B, complete sequence

gi|336122587|ref|NC_015633.1| Vibrio anguillarum 775 chromosome I, complete sequence

gi|336125405|ref|NC_015637.1| Vibrio anguillarum 775 chromosome II, complete sequence

2) All_Vector.fsa file was generated using txt editor combining vector genomes

$ /ncbi-blast-2.2.31+/bin/makeblastdb -in Vector_Genome_fa/All_Vector.fsa -parse_seqids -dbtype nucl -out nt

$ /ncbi-blast-2.2.31+/bin/blastn -task megablast -db nt -perc_identity 90 -max_target_seqs 1 -outfmt "7 qacc qstart qend sacc sstart send sstrand" -query Trinity.fasta -out result_.out -num_threads 4

3) good_ids.txt was generated by filtering vector ids from trinity.fasta ids using linux commands and assembly was cleaned using following commands:

$ perl -ne 'if(/^>(\S+)/){$c=$i{$1}}$c?print:chomp;$i{$_}=1 if @ARGV' good_ids.txt Trinity.fasta > Trinity_Cleaned.fasta

Note: After removing vector contamination, assembly was renamed as Trinity_Cleaned.fasta

Trinity Assembly_Stat

$ /trinityrnaseq-2.0.6/util/TrinityStats.pl Trinity_Cleaned.fasta

3. Reference Preparation, Alignment and Abundance Estimation (n=13)
##

Ref Preparation:

$ /trinityrnaseq-2.0.6/util/align_and_estimate_abundance.pl --transcripts Trinity_Cleaned.fasta --est_method RSEM --aln_method bowtie --trinity_mode --prep_reference --SS_lib_type RF --output_prefix --debug --thread_count 20

Abundance Estimation: individual run for each sample using bowtie and RSEM (below is example for first sample only)

$ /trinityrnaseq-2.0.6/util/align_and_estimate_abundance.pl --transcripts Trinity_Cleaned.fasta --seqType fq --left 1-Haugland_ATCACG_L008_R1_001.fastq.gz.PwU.qtrim.fq --right 1-Haugland_ATCACG_L008_R2_001.fastq.gz.PwU.qtrim.fq --est_method RSEM --aln_method bowtie --trinity_mode --SS_lib_type RF --output_dir Sample_1_out --output_prefix Sample_1_ --debug --thread_count 5

Detailed Assessment of Read Content of the Assembly

$ cd Sample_1_out

$ /trinityrnaseq-2.0.6/util/bowtie_PE_separate_then_join.pl --seqType fq --left 1-Haugland_ATCACG_L008_R1_001.fastq.gz.PwU.qtrim.fq --right 1-Haugland_ATCACG_L008_R2_001.fastq.gz.PwU.qtrim.fq --target Trinity_Cleaned.fasta --aligner bowtie --SS_lib_type RF --retain_intermediate_files -- -p 4 --all --best --strata -m 300 > bowtie_PE_separate_then_join.out

$ /trinityrnaseq-2.0.6/util/SAM_nameSorted_to_uniq_count_stats.pl bowtie_out/bowtie_out.nameSorted.bam > SAM_nameSorted_to_uniq_count_stats.out

4. Expression Value Matrices Generation
######################################

$ /trinityrnaseq-2.0.6/util/abundance_estimates_to_matrix.pl --est_method RSEM --cross_sample_fpkm_norm TMM --out_prefix Trinity_genes Sample_1_.genes.results Sample_2_.genes.results Sample_3_.genes.results Sample_4_.genes.results Sample_5_.genes.results Sample_6_.genes.results Sample_7_.genes.results Sample_8_.genes.results Sample_9_.genes.results Sample_10_.genes.results Sample_11_.genes.results Sample_12_.genes.results Sample_13_.genes.results > genes_abundance_estimates_to_matrix.pl.out

5. Generic Contaminants Generation
###################################

generating and removing generic contaminants contigs by blasting against NT for clearly non-eukaryote sequences
1) the contigs of the assembly was split into 20 blast jobs, example commandline

```
$ blastn -query lumpfish_unclean00.fa -db nt -num_threads 4 -outfmt '6 qseqid sseqid evalue staxids sskingdoms' 2> lumpfish_unclean00.fa_blast_results.log | gzip -c > lumpfish_unclean00.fa_blast_results.txt.gz
```

2) the blast results were parsed to compute an Alien Index [**Gladyshev et al, 2008**], a corrected log ratio between the E-value for the best Eukaryote and best non-Eukaryote blast hits of each contig. We used an Alien Index >= 45, corresponding to an E-value >= 20 orders of magnitude difference between the best non-metazoan hit to the best metazoan hit, to identify 4061 non-Eukaryote contigs most likely being contaminant. These were removed from the transcript set and not used in subsequent analysis.

```
$ for FN in `ls lumpfish_*blast_results.txt.gz`; do alienIndex -c 4 -e 2 -m Eukaryota -i <( zcat $FN) > ai_output.${FN}.txt; done
```

```
$ cat ai_output* | perl -lane 'print if $F[3] > 45' | cut -f1 > trId_isBacterial.txt
```

6. Generic Contaminants Removal
################################
Note: generic contaminants were removed from expression matrices before DEG detection. This was done as part of the R-script provided below using geneId_isContaminant.txt. Ids in trId_isBacterial.txt file were in c_g_i (contig_gene_isoform) format and only isoform ids (_i) were removed to generate geneId_isContaminant.txt file. geneId_isContaminant.txt file contained only gene Ids (c_g).

7. DEG detection (p-value 0.1)
############################

The differential expression analysis was performed in R using the limma-voom method in the limma package [Ritchie et al 2015] based on the RSEM read count estimates per gene (Trinity_genes.counts.matrix). see R-script provided.

8. TransDecoder for Trinotate
############################

```
$ TransDecoder.LongOrfs -t /export/kjempetujafs/service/projects/2015-06_Haugland_transcriptome/WD_Charitra/TransDecoder_out_dir/Trinity_Cleaned_perl.fasta
```

```
$ ncbi-blast-2.2.31+/bin/blastp -query Trinity_Cleaned_perl.fasta.transdecoder_dir/longest_orfs.pep -db uniprot_sprot.trinotate.pep -max_target_seqs 1 -outfmt 6 -evalue 1e-5 -num_threads 30 > blastp.outfmt6
```

```
$ hmmer-3.1b2-linux-intel-x86_64/binaries/hmmscan --cpu 30 --domtblout pfam.domtblout Pfam-A.hmm longest_orfs.pep & tail -f nohup.out
```

```
$ TransDecoder-2.0.1/TransDecoder.Predict -t Trinity_Cleaned.fasta --retain_pfam_hits pfam.domtblout --retain_blastp_hits blastp.outfmt6
```

```
### 9. Annotations by Trinotate
##########################
## Trinotate: https://trinotate.github.io

# Capturing BLAST Homologies

$ ncbi-blast-2.2.31+/blastx -query Trinity_Cleaned_perl.fasta -db uniprot_uniref90.trinotate.pep -num_threads 8 -max_target_seqs 1 -outfmt 6 > uniref90.blastx.outfmt6

$ ncbi-blast-2.2.31+/blastp -query Trinity_Cleaned_perl.fasta.transdecoder.pep -db uniprot_uniref90.trinotate.pep -num_threads 8 -max_target_seqs 1 -outfmt 6 > uniref90.blastp.outfmt6

# Running HMMER

$ hmmer-3.1b2-linux-intel-x86_64/binaries/hmmscan --cpu 8 --domtblout TrinotatePFAM.out Pfam-A.hmm Trinity_Cleaned.fasta.transdecoder.pep > pfam.log

# Running signalP to predict signal peptides

$ signalp-4.1/signalp -f short -n signalp.out Trinity_Cleaned.fasta.transdecoder.pep

# Running tmHMM to predict transmembrane regions

$ tmhmm-2.0c/bin/tmhmm --short < Trinity_Cleaned.fasta.transdecoder.pep > tmhmm.out

# Running RNAMMER

$ perl /Trinotate-2.0.2/util/rnammer_support/RnammerTranscriptome.pl --transcriptome Trinity_Cleaned.fasta --path_to_rnammer rnammer-1.2.src/rnammer

# Loading Above Results into a Trinotate SQLite Database

$ Trinotate /Trinotate-2.0.2/Trinotate.sqlite init --gene_trans_map Trinity.fasta.gene_trans_map --transcript_fasta Trinity_Cleaned.fasta --transdecoder_pep Trinity_Cleaned.fasta.transdecoder.pep

$ Trinotate /Trinotate-2.0.2/Trinotate.sqlite LOAD_swissprot_blastp blastp.outfmt6

$ Trinotate /Trinotate-2.0.2/Trinotate.sqlite LOAD_swissprot_blastx blastx.outfmt6
```

$ Trinotate /Trinotate-2.0.2/Trinotate.sqlite LOAD_pfam TrinotatePFAM.out

$ Trinotate /Trinotate-2.0.2/Trinotate.sqlite LOAD_tmhmm tmhmm.out

$ Trinotate /Trinotate-2.0.2/Trinotate.sqlite LOAD_signalp signalp.out

$ Trinotate /Trinotate-2.0.2/Trinotate.sqlite report > trinotate_annotation_report.xls

10. Adding Annotations to DEG
##############################
Annotations in .xls file were added to DEG table using simple text/excel editor.

R-script
##########

```
```{r config}

library(limma)

library(edgeR)

library(dplyr)

library(ggplot2)


#library(DESeq2)


#setwd("")

```
```

Load RSEM read count estimates per gene

```
```{r loadData, echo="FALSE"}

x = read.delim("../Trinity_genes.counts.matrix")


names(x) = c('gene_id',

        'ctrl_6h_r1','ctrl_6h_r2','ctrl_6h_r3',

        'treat_6h_r1','treat_6h_r2','treat_6h_r3',
```
```

'ctrl_24h_r1','ctrl_24h_r2','ctrl_24h_r3',

'treat_24h_r1','treat_24h_r2','treat_24h_r3',

'ipnv_24h_r1')

row.names(x)=x$gene_id

```

2016-04 Remove contaminants (detected by BLAST analysis)

```{r rmContaminants}
isContaminant=read.table("..geneId_isContaminant.txt")
isContaminant = as.character(isContaminant$V1)
x = x[!x$gene_id %in% isContaminant,]
```

```{r, echo=FALSE}
x=x[,-1] #strip gene_id column
```

Remove genes that are not appreciably expressed (min 10 reads)
```

```{r dropLowAbundant}

keep=rowSums(x>10) >= 3 #at least 10 reads in at least 3 samples

table(keep) #keep 34280 genes/221k

x = x[keep,]

```

Set up experimental design matrix

```{r designMatrix}

treatment <- factor(c('ctrl_6h','ctrl_6h','ctrl_6h','treat_6h','treat_6h','treat_6h',

                'ctrl_24h','ctrl_24h','ctrl_24h','treat_24h','treat_24h','treat_24h',

                'inpv'), levels=c('ctrl_6h','treat_6h','ctrl_24h','treat_24h','inpv')) #ctrl_mo experimental design

sample_pool <- factor(c(1,2,3,1,2,3,1,2,3,1,2,3,3), levels=c(1,2,3))


design <- model.matrix(~0 + treatment + sample_pool)

data.frame(Sample=colnames(x),treatment,sample_pool)


rownames(design) <- colnames(x)

```

limma-voom analysis

```{r limma_scale_counts}

y <- DGEList(counts=x,group=treatment)

y <- calcNormFactors(y)

v <- voom(y,design,plot=TRUE)

plotMDS(v,top=50,labels=treatment,

        col=ifelse(treatment=="treat_6h","blue","red"),gene.selection="common")

fit <- lmFit(v,design)

fit <- eBayes(fit)


```

Examine the different contrasts (treatment vs control at each time-point)

```{r limmaDEResults}

cont.matrix <- makeContrasts(TvsC_6h=treatmenttreat_6h-treatmentctrl_6h,

                             TvsC_24h=treatmenttreat_24h-treatmentctrl_24h,

                             INPVvsC=treatmentinpv-treatmentctrl_24h,

#                              TvsT=treatmenttreat_24h-treatmenttreat_6h,
```

```
#                          CvsC=treatmentctrl_24h-Intercept,
                         levels=design)
#cont.matrix
#row.names(cont.matrix) = c("(Intercept)","sample_pool2","sample_pool3",
#                        "treatmenttreat_6h","treatmentctrl_24h","treatmenttreat_24h",
#                        "treatmentinpv") #fix for (Intercept) vs Intercept

fit2 <- contrasts.fit(fit, contrast=cont.matrix)
fit2 <- eBayes(fit2)

#fit2 <- contrasts.fit(fit, contrast=c(-1,0,0,1,0,0,0)) #treat vs ctrl 6h
#fit2 <- eBayes(fit2)
top_TvsC_6h = topTable(fit2, coef="TvsC_6h", number = Inf, adjust="BH")
sum(top_TvsC_6h$adj.P.Val<0.05)
sum(top_TvsC_6h$adj.P.Val<0001)
#head(top_TvsC_6h)
#x['TR31519|c0_g1',]
#x['TR35037|c0_g2',]

top_TvsC_24h = topTable(fit2, coef="TvsC_24h", number = Inf, adjust="BH")
```

```
sum(top_TvsC_24h$adj.P.Val<0.05)

sum(top_TvsC_24h$adj.P.Val<0001)


#fit2 <- contrasts.fit(fit, contrast=c(0,0,0,0,-1,0,1)) #INPV vs ctrl 24h

#fit2 <- eBayes(fit2)

top_VcsC = topTable(fit2, coef="INPVvsC", number= Inf, adjust="BH")


#fit2 <- contrasts.fit(fit, contrast=c(0,0,0,-1,0,1,0)) #treat 24h vs treat_6h

#fit2 <- eBayes(fit2)

#topTable(fit2, coef="TvsT", adjust="BH")


#fit2 <- contrasts.fit(fit, contrast=c(-1,0,0,0,1,0,0)) #ctrl 24h vs ctrl_6h

#fit2 <- eBayes(fit2)

#topTable(fit2, coef="CvsC", adjust="BH")

###
```

Merge 6h and 24h results for QC and plots

merge the DE contrast tables for export

~~~
```{r mergeTimepoints}

top_6_24 = merge(top_TvsC_6h, top_TvsC_24h,by="row.names",all.x=TRUE)

names(top_6_24) = c('geneID',

                  'logFC_6h',

                  'avgExpr_6h',

                  't_6h',

                  'pval_6h',

                  'adjpval_6h',

                  'b_6h',

                  'logFC_24h',

                  'avgExpr_24h',

                  't_24h',

                  'pval_24h',

                  'adjpval_24h',

                  'b_24h')
```
~~~

QC: Examine the concordance of the two timepoints

~~~
```{r rankPlot}
~~~

```
rank_6h = top_TvsC_6h %>% mutate(rank = rank(adj.P.Val)) %>% select(rank)

rank_24h = top_TvsC_24h %>% mutate(rank = rank(adj.P.Val)) %>% select(rank)

rank_6_24 = merge(rank_6h, rank_24h, by="row.names",all.x=TRUE)


cor(rank_6_24$rank.x,rank_6_24$rank.y, method="spearman") #spearman corr 0.90


ggplot(rank_6_24, aes(x=rank.x,y=rank.y)) + geom_point(aes(alpha=0.1)) +

  labs(title="Concordance of gene DE P-value rank at 6 and 24 hrs",

       x="P-value rank 6 hrs",

       y="P-value rank 24 hrs") +

       theme(legend.position = "none")


#top 100

ggplot(rank_6_24, aes(x=rank.x,y=rank.y)) + geom_point(aes(alpha=0.1)) +

  labs(title="Concordance of gene DE P-value rank at 6 and 24 hrs",

       x="P-value rank 6 hrs",

       y="P-value rank 24 hrs") +

       theme(legend.position = "none") +

       xlim(0, 100) +

       ylim(0,100)
```

```

Conclusion: The genes that are differentially regulated at 6 hrs are also diff regulated at 24 hrs

Now, diagnostic exploration of of p-values - se DESeq manual for background

```{r pvaldist}
plot_pval_diag = function(padj){
  orderInPlot = order(padj)
  showInPlot = (padj[orderInPlot] <= 0.01)
  alpha = 0.01
  plot(seq(along=which(showInPlot)), padj[orderInPlot][showInPlot], pch=".", xlab = expression(rank(p[i])), ylab=expression(p[i]))
  abline(a=0, b=alpha/length(padj), col="red3", lwd=2)
}
plot_pval_diag(top_6_24$adjpval_6h)
plot_pval_diag(top_6_24$adjpval_24h)

plot_pval_cutoff_comp = function(padj){
  padj.log <- -log10(padj)
```
```

```
  orderInPlot = order(padj.log)

  plot(padj.log[orderInPlot], type="l")

  sigline <- c(.05, .01, 5, 1,05, 01)

  sigline <- -log10(sigline)

  sigcolors <- c("red", "blue", "green", "yellow","pink","purple")

  sapply(1:length(sigline), function(x){abline(h=sigline[padj.log], col=sigcolors[padj.log])})

}

plot_pval_cutoff_comp(top_6_24$adjpval_6h)

plot_pval_cutoff_comp(top_6_24$adjpval_24h)


plot_pval_histograms = function(pval){

  hist(top_6_24$adjpval_6h, breaks=5000, ylim = c(0,100))

  hist(top_6_24$adjpval_6h, breaks=2000, xlim = c(0,0.1))

  hist(top_6_24$adjpval_6h, breaks=1000000, xlim = c(0,0.01))

  hist(top_6_24$adjpval_6h, breaks=1000000, xlim = c(0,02))

  hist(top_6_24$adjpval_6h, breaks=1000000, xlim = c(0,005))

  hist(top_6_24$adjpval_6h, breaks=1000000, xlim = c(0,0005))

}


#  ggplot(top_6_24, aes(adjpval_2h)) + geom_histogram(bins=5000) + ylim(0,100)
```

ggplot(top_6_24, aes(adjpval_6h)) + geom_histogram(bins=2000) + xlim(0,0.1)

ggplot(top_6_24, aes(adjpval_6h)) + geom_histogram(bins=2000) + xlim(0,0.01)

ggplot(top_6_24, aes(adjpval_6h)) + geom_histogram(bins=2000) + xlim(0,02)

ggplot(top_6_24, aes(adjpval_6h)) + geom_histogram(bins=2000) + xlim(0,002)

ggplot(top_6_24, aes(adjpval_6h)) + geom_histogram(bins=2000) + xlim(0,0002)

plot_pval_histograms(top_6_24$adjpval_6h)

plot_pval_histograms(top_6_24$adjpval_24h)

```

Volcano plot

```{r volcanoPlot, eval=FALSE, echo=FALSE}

res <- top_6_24 %>% dplyr::select(geneID, logFC_6h,pval_6h, adjpval_6h)

names(res) = c("geneID","log2FoldChange", "pvalue", "padj")

# Make a basic volcano plot

with(res, plot(log2FoldChange, -log10(pvalue), pch=20, main="Volcano plot", xlim=c(-2.5,2)))

```
Add colored points: red if padj<0.05, orange of log2FC>1, green if both)

with(subset(res, padj<001), points(log2FoldChange, -log10(pvalue), pch=20, col="red"))

with(subset(res, abs(log2FoldChange)>1), points(log2FoldChange, -log10(pvalue), pch=20, col="orange"))

with(subset(res, padj<001 & abs(log2FoldChange)>1), points(log2FoldChange, -log10(pvalue), pch=20, col="green"))

Label points with the textxy function from the calibrate plot

library(calibrate)

with(subset(res, padj<001 & abs(log2FoldChange)>1), textxy(log2FoldChange, -log10(pvalue), labs=geneID, cex=.8))

#volcano plot

#scattering the M values (log2 ratio) on the x axis against the p value (-log10 the p value).

ggplot(top_6_24, aes(x=logFC_6h, y = -log10(adjpval_6h))) + geom_point(aes(alpha=0.1))

detach("package:calibrate", unload=TRUE) #calibrate select conflicts with dplyr
```

Export DE Tables

```{r exportDETables}
top_6_24_out = top_6_24 %>% select(geneID,logFC_6h,avgExpr_6h,adjpval_6h,logFC_24h,avgExpr_24h,adjpval_24h) %>% arrange(adjpval_24h)

write.table(top_6_24_out, file="differential_expression_table.txt", quote=FALSE, sep = "\t")
```

```
#down 6h

downreg_6 = top_6_24 %>% filter(adjpval_6h < 1e-5 & logFC_6h < 0) %>%select(geneID,logFC_6h,avgExpr_6h,adjpval_6h) %>%
arrange(adjpval_6h)

write.table(downreg_6, file="differential_expression_down_6h.txt",

        quote=FALSE, sep = "\t", row.names = FALSE)


upreg_6 = top_6_24 %>% filter(adjpval_6h < 1e-5 & logFC_6h > 0) %>%select(geneID,logFC_6h,avgExpr_6h,adjpval_6h) %>% arrange(adjpval_6h)

write.table(upreg_6, file="differential_expression_up_6h.txt",

        quote=FALSE, sep = "\t", row.names = FALSE)


downreg_24 = top_6_24 %>% filter(adjpval_24h < 1e-5 & logFC_24h < 0) %>%select(geneID,logFC_24h,avgExpr_24h,adjpval_24h) %>%
arrange(adjpval_24h)

write.table(downreg_24, file="differential_expression_down_24h.txt",

        quote=FALSE, sep = "\t", row.names = FALSE)


upreg_24 = top_6_24 %>% filter(adjpval_24h < 1e-5 & logFC_24h > 0) %>%select(geneID,logFC_24h,avgExpr_24h,adjpval_24h) %>%
arrange(adjpval_24h)

write.table(upreg_24, file="differential_expression_up_24h.txt",

        quote=FALSE, sep = "\t", row.names = FALSE)


# write labels file for Trinity GO analysis (label\tgeneid)
```

```
# because row.names (geneIds) can not be repeated, we do 6h and 24h separately
labels_6h = rbind(
data.frame('label' = rep("down_6",nrow(downreg_6)), 'geneID' = downreg_6$geneID),
data.frame('label' = rep("up_6",nrow(upreg_6)), 'geneID' = upreg_6$geneID))
write.table(labels_6h, file="differential_expression_labels_for_go_6h.txt",
        quote=FALSE, sep = "\t", row.names = FALSE, col.names = FALSE)

labels_24h = rbind(
data.frame('label' = rep("down_24",nrow(downreg_24)), 'geneID' = downreg_24$geneID),
data.frame('label' = rep("up_24",nrow(upreg_24)), 'geneID' = upreg_24$geneID)
)
write.table(labels_24h, file="differential_expression_labels_for_go_24h.txt",
        quote=FALSE, sep = "\t", row.names = FALSE, col.names = FALSE)

```

Running the Go overrep code through trinity

```
```{r gooverrep, engine="bash", eval=FALSE}
```
```

/trinity/Analysis/DifferentialExpression/run_GOseq.pl --genes_single_factor=differential_expression_labels_6h.txt --GO_assignments=go_annotations.txt --lengths=gene_length.txt > go_overrep_6h.txt

/trinity/Analysis/DifferentialExpression/run_GOseq.pl --genes_single_factor=differential_expression_labels_24h.txt --GO_assignments=go_annotations.txt --lengths=gene_length.txt > go_overrep_24h.txt

```

DESeq2 plots for QA

```{r DESeq2Plots}
countData = round(x)
colData = data.frame(sample_pool,treatment)
row.names(colData) = colnames(countData)
library(DESeq2)
dds <- DESeqDataSetFromMatrix(countData = countData,
                              colData = colData,
                              design = ~ sample_pool + treatment)
dds <- DESeq(dds)

rld <- rlog(dds)

````
vsd <- varianceStabilizingTransformation(dds)

rlogMat <- assay(rld)

vstMat <- assay(vsd)

```


```{r gene_heatmap_1-30}

library("RColorBrewer")

library("gplots")

select <- order(rowMeans(counts(dds,normalized=TRUE)),decreasing=TRUE)[1:30]

hmcol <- colorRampPalette(brewer.pal(9, "GnBu"))(100)


heatmap.2(assay(vsd)[select,], col = hmcol,

        Rowv = FALSE, Colv = FALSE, scale="none",

        dendrogram="none", trace="none", margin=c(10, 6))


```


```{r gene_heatmap_1-100}

library("RColorBrewer")

library("gplots")
````

````
select <- order(rowMeans(counts(dds,normalized=TRUE)),decreasing=TRUE)[1:100]
hmcol <- colorRampPalette(brewer.pal(9, "GnBu"))(100)

heatmap.2(assay(vsd)[select,], col = hmcol,
    Rowv = FALSE, Colv = FALSE, scale="none",
    dendrogram="none", trace="none", margin=c(10, 6))

```

```{r gene_heatmap_genefilter_rowVars_1-30}
library("RColorBrewer")
library("gplots")
select <- order(genefilter::rowVars(counts(dds,normalized=TRUE)),decreasing=TRUE)[1:30]
hmcol <- colorRampPalette(brewer.pal(9, "GnBu"))(100)

heatmap.2(assay(vsd)[select,], col = hmcol,
    Rowv = FALSE, Colv = FALSE, scale="none",
    dendrogram="none", trace="none", margin=c(10, 6))

```
````
````

```
```{r gene_heatmap_genefilter_rowVars_1-100}

library("RColorBrewer")

library("gplots")

select <- order(genefilter::rowVars(counts(dds,normalized=TRUE)),decreasing=TRUE)[1:100]

hmcol <- colorRampPalette(brewer.pal(9, "GnBu"))(100)


heatmap.2(assay(vsd)[select,], col = hmcol,

        Rowv = FALSE, Colv = FALSE, scale="none",

        dendrogram="none", trace="none", margin=c(10, 6))


```

```{r sample_heatmap}

library(ggplot2)


distsRL <- dist(t(assay(rld)))

mat <- as.matrix(distsRL)

rownames(mat) <- colnames(mat) <- with(colData(dds),

                            paste(sample_pool, treatment, sep=" : "))

hc <- hclust(distsRL)
```
```

```
heatmap.2(mat, Rowv=as.dendrogram(hc),

          symm=TRUE, trace="none",

          col = rev(hmcol), margin=c(13, 13))


#plotPCA(rld, intgroup=c("treatment", "sample_pool"))


data <- plotPCA(rld, intgroup=c("treatment", "sample_pool"), returnData=TRUE)

percentVar <- round(100 * attr(data, "percentVar"))

ggplot(data, aes(PC1, PC2, color=sample_pool, shape=treatment)) +

  geom_point(size=3) +

  xlab(paste0("PC1: ",percentVar[1],"% variance")) +

  ylab(paste0("PC2: ",percentVar[2],"% variance"))
```

# Supplementary Results of Trinity RSEM

**Table 1.** Read count matrix from RSEM output

| | Sample | | proper_pairs | total aligned | improper_pairs | left_only | right_only | Genes expressed ≥ 2 FPKM | Genes expressed ≥ 1 FPKM | Genes expressed Total |
|---|---|---|---|---|---|---|---|---|---|---|
| Control-1 | **1** | count | 24,189,368 | 29,980,237 | 3,976,458 | 1,137,983 | 676,428 | 39,506 | 66,391 | 221,659 |
| | | pct | 80.68 | -- | 13.26 | 3.8 | 2.26 | -- | -- | -- |
| | **2** | count | 27,178,664 | 32,937,998 | 4,168,232 | 956,390 | 634,712 | 36,077 | 65,219 | 221,659 |
| | | pct | 82.51 | -- | 12.65 | 2.9 | 1.93 | -- | -- | -- |
| | **3** | count | 28,659,252 | 37,114,067 | 6,626,260 | 1,097,874 | 730,681 | 36,250 | 66,468 | 221,659 |
| | | pct | 77.22 | -- | 17.85 | 2.96 | 1.97 | -- | -- | -- |
| Treated-1 | **4** | count | 24,899,860 | 31,065,905 | 4,472,704 | 1,028,504 | 664,837 | 36,935 | 65,105 | 221,659 |
| | | pct | 80.15 | -- | 14.4 | 3.31 | 2.14 | -- | -- | -- |
| | **5** | count | 21,999,490 | 27,276,150 | 3,793,870 | 886,000 | 596,790 | 37,203 | 63,440 | 221,659 |
| | | pct | 80.65 | -- | 13.91 | 3.25 | 2.19 | -- | -- | -- |
| | **6** | count | 24,627,776 | 30,329,773 | 4,052,902 | 985,884 | 663,211 | 37,970 | 66,678 | 221,659 |
| | | pct | 81.2 | -- | 13.36 | 3.25 | 2.19 | -- | -- | -- |
| Control-2 | **7** | count | 26,683,856 | 33,281,629 | 4,862,120 | 1,062,160 | 673,493 | 36,291 | 62,730 | 221,659 |
| | | pct | 80.18 | -- | 14.61 | 3.19 | 2.02 | -- | -- | -- |
| | **8** | count | 23,200,556 | 29,769,028 | 4,832,936 | 1,025,958 | 709,578 | 38,483 | 64,273 | 221,659 |
| | | pct | 77.94 | -- | 16.23 | 3.45 | 2.38 | -- | -- | -- |
| | **9** | count | 26,758,676 | 32,816,055 | 4,297,730 | 1,060,466 | 699,183 | 36,797 | 63,158 | 221,659 |
| | | pct | 81.54 | -- | 13.1 | 3.23 | 2.13 | -- | -- | -- |
| Treated-2 | **10** | count | 25,713,468 | 31,888,148 | 4,358,192 | 1,117,602 | 698,886 | 35,393 | 60,377 | 221,659 |
| | | pct | 80.64 | -- | 13.67 | 3.5 | 2.19 | -- | -- | -- |
| | **11** | count | 23,517,108 | 29,484,868 | 4,255,492 | 1,038,233 | 674,035 | 37,826 | 64,233 | 221,659 |
| | | pct | 79.76 | -- | 14.43 | 3.52 | 2.29 | -- | -- | -- |
| | **12** | count | 22,513,278 | 27,487,774 | 3,394,564 | 949,623 | 630,309 | 39,037 | 67,158 | 221,659 |
| | | pct | 81.9 | -- | 12.35 | 3.45 | 2.29 | -- | -- | -- |
| Treated-3 | **13** | count | 20,204,506 | 26,674,188 | 4,519,114 | 1,212,334 | 738,234 | 39,580 | 65,727 | 221,659 |
| | | pct | 75.75 | -- | 16.94 | 4.54 | 2.77 | -- | -- | -- |

Note: Vector contaminated seq were removed before RSEM output.

```
################################
## Counts of transcripts, etc.
################################
Total trinity 'genes':	221659
Total trinity transcripts:	346430
Percent GC: 46.65

########################################
Stats based on ALL transcript contigs:
########################################

	Contig N10: 6782
	Contig N20: 4988
	Contig N30: 3947
	Contig N40: 3144
	Contig N50: 2502

	Median contig length: 585
	Average contig: 1250.14
	Total assembled bases: 433087424


#####################################################
## Stats based on ONLY LONGEST ISOFORM per 'GENE':
#####################################################

	Contig N10: 5514
	Contig N20: 3647
	Contig N30: 2446
	Contig N40: 1608
	Contig N50: 1071

	Median contig length: 401
	Average contig: 735.04
	Total assembled bases: 162928446
```

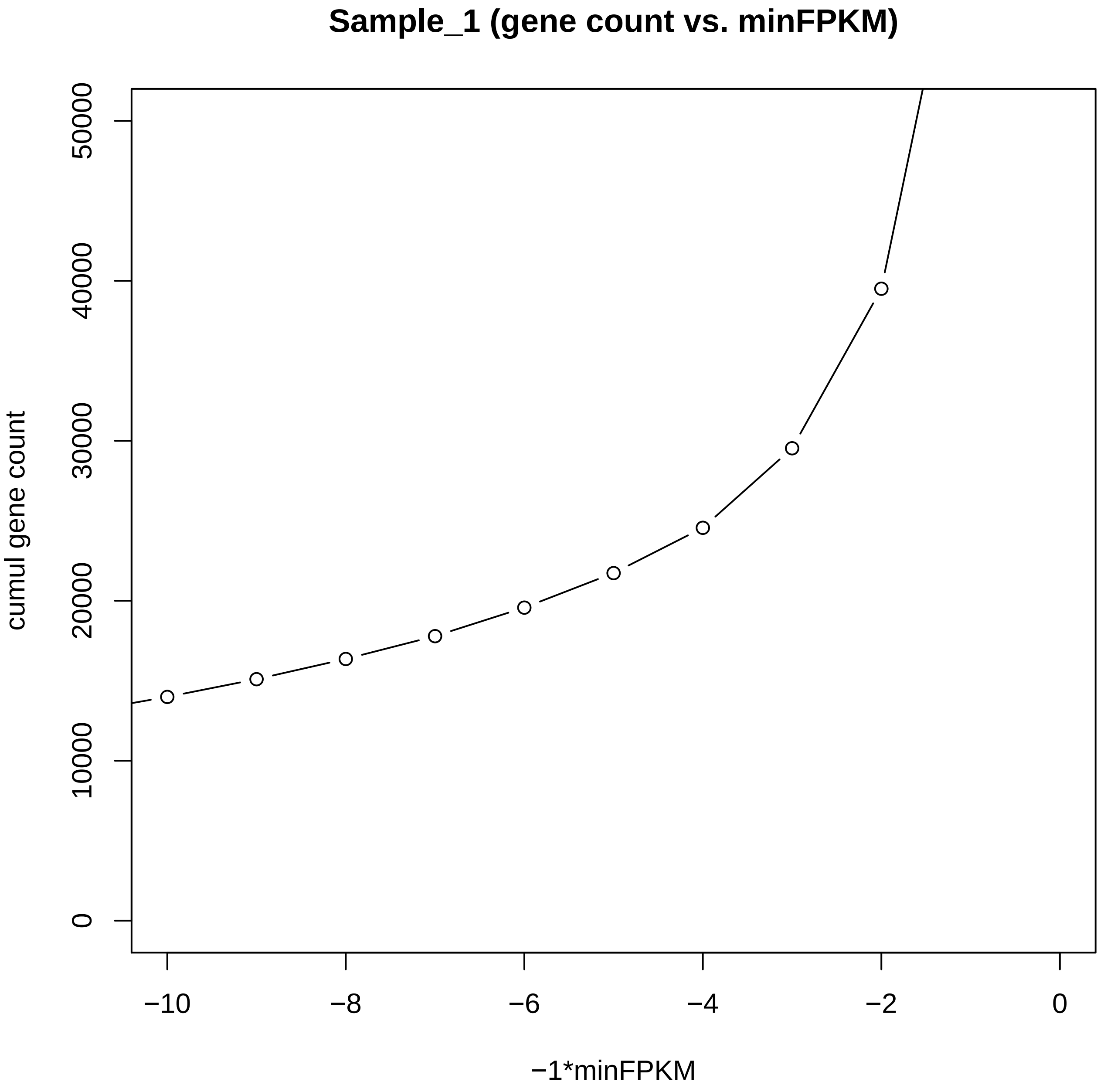
Sample_1 (gene count vs. minFPKM)
cumul gene count
0
10000
20000
30000
40000
50000
−10
−8
−6
−4
−2
0
−1*minFPKM

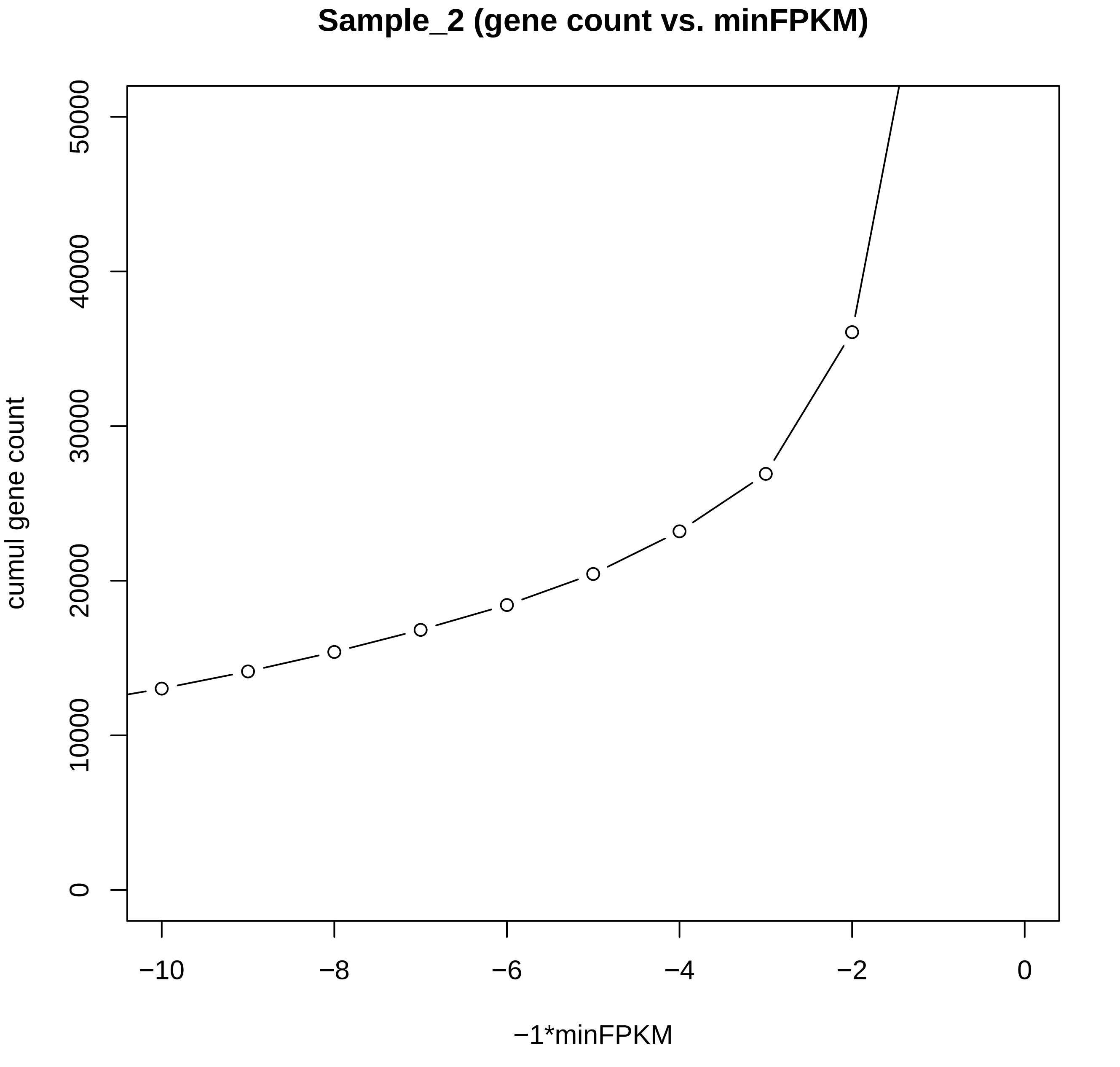
Sample_2 (gene count vs. minFPKM)
cumul gene count
0
10000
20000
30000
40000
50000
−10
−8
−6
−4
−2
0
−1*minFPKM

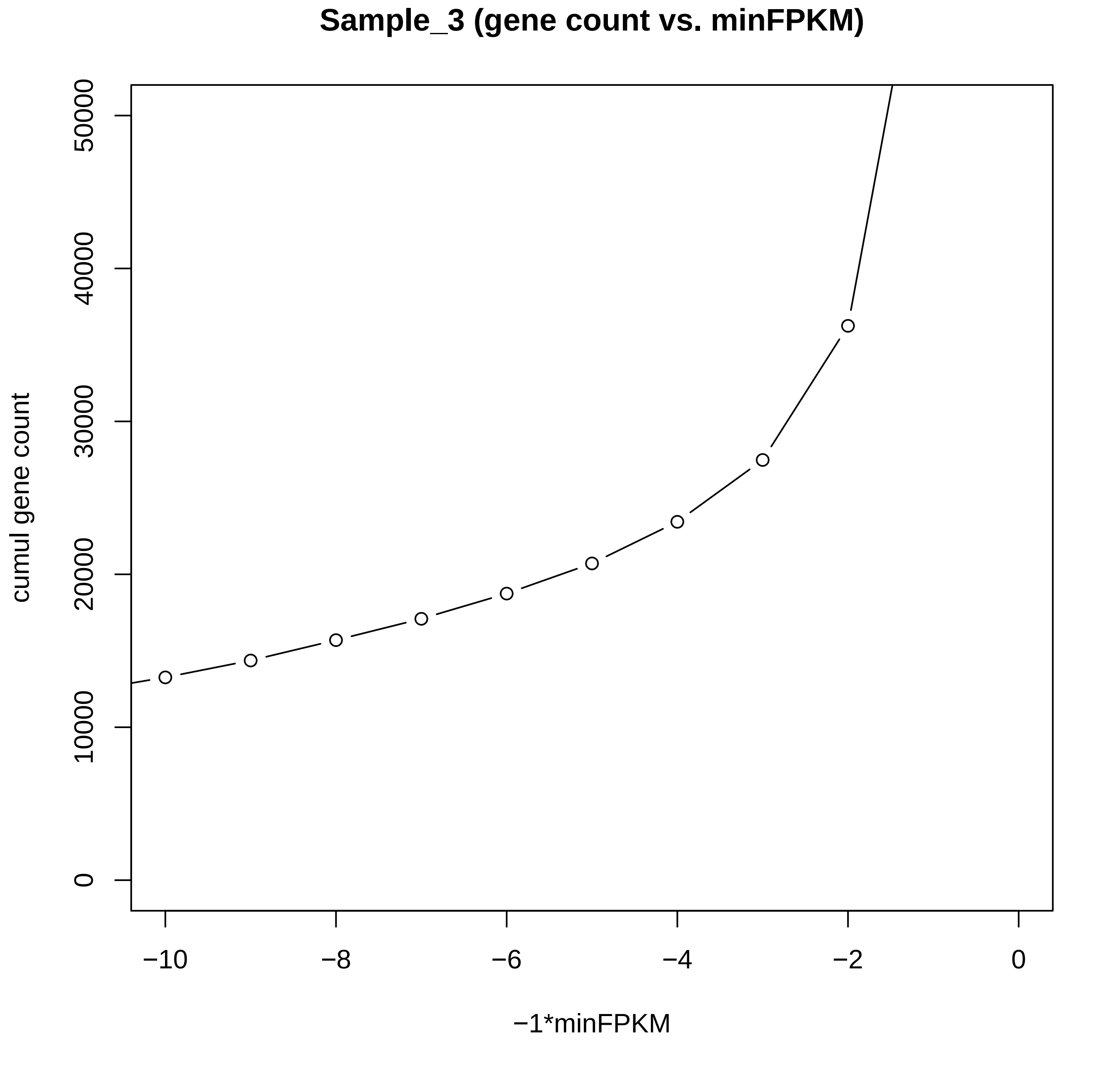
Sample_3 (gene count vs. minFPKM)
cumul gene count
0
10000
20000
30000
40000
50000
−10
−8
−6
−4
−2
0
−1*minFPKM

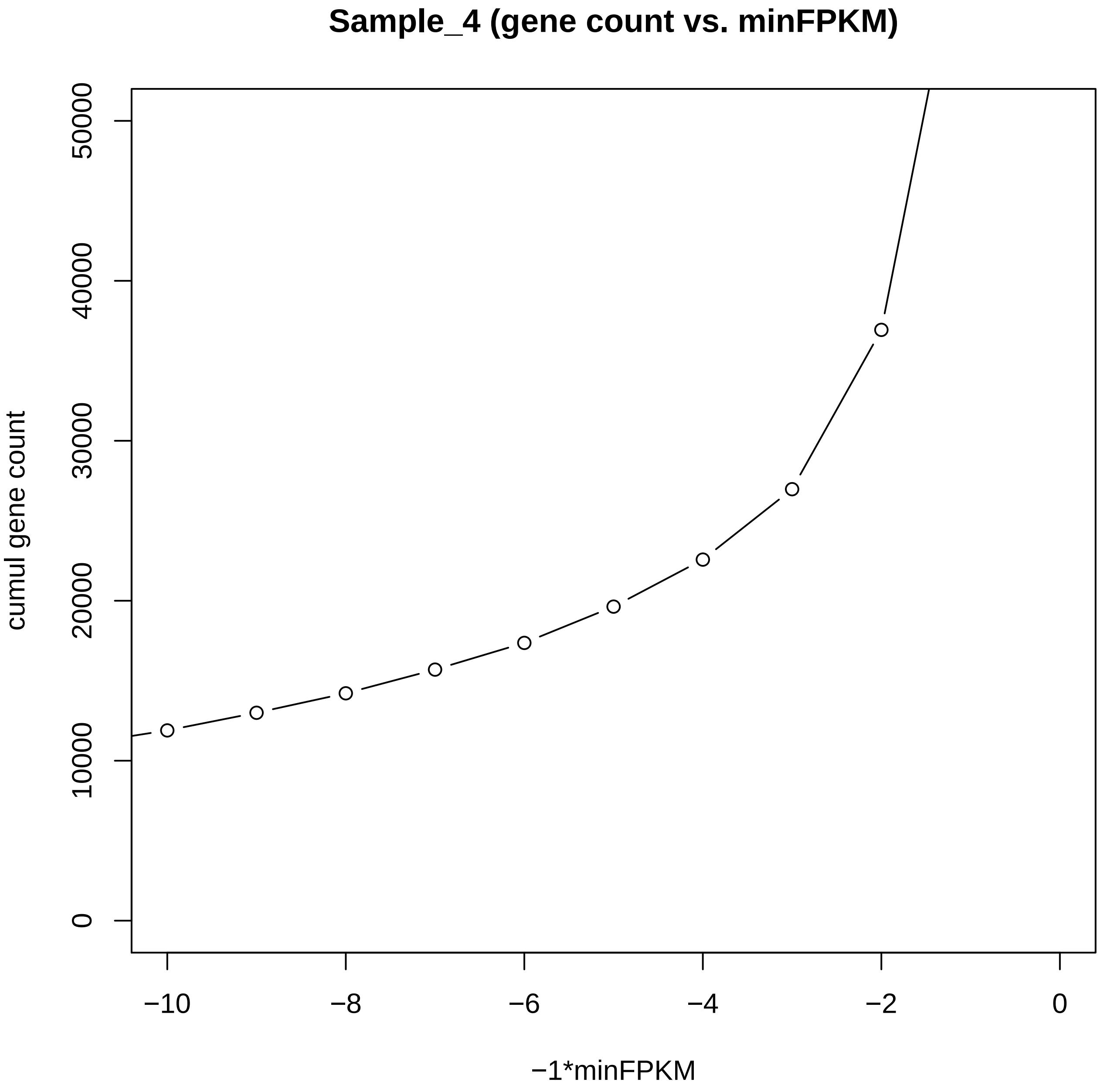

Sample_4 (gene count vs. minFPKM)
cumul gene count
0
10000
20000
30000
40000
50000
−10
−8
−6
−4
−2
0
−1*minFPKM

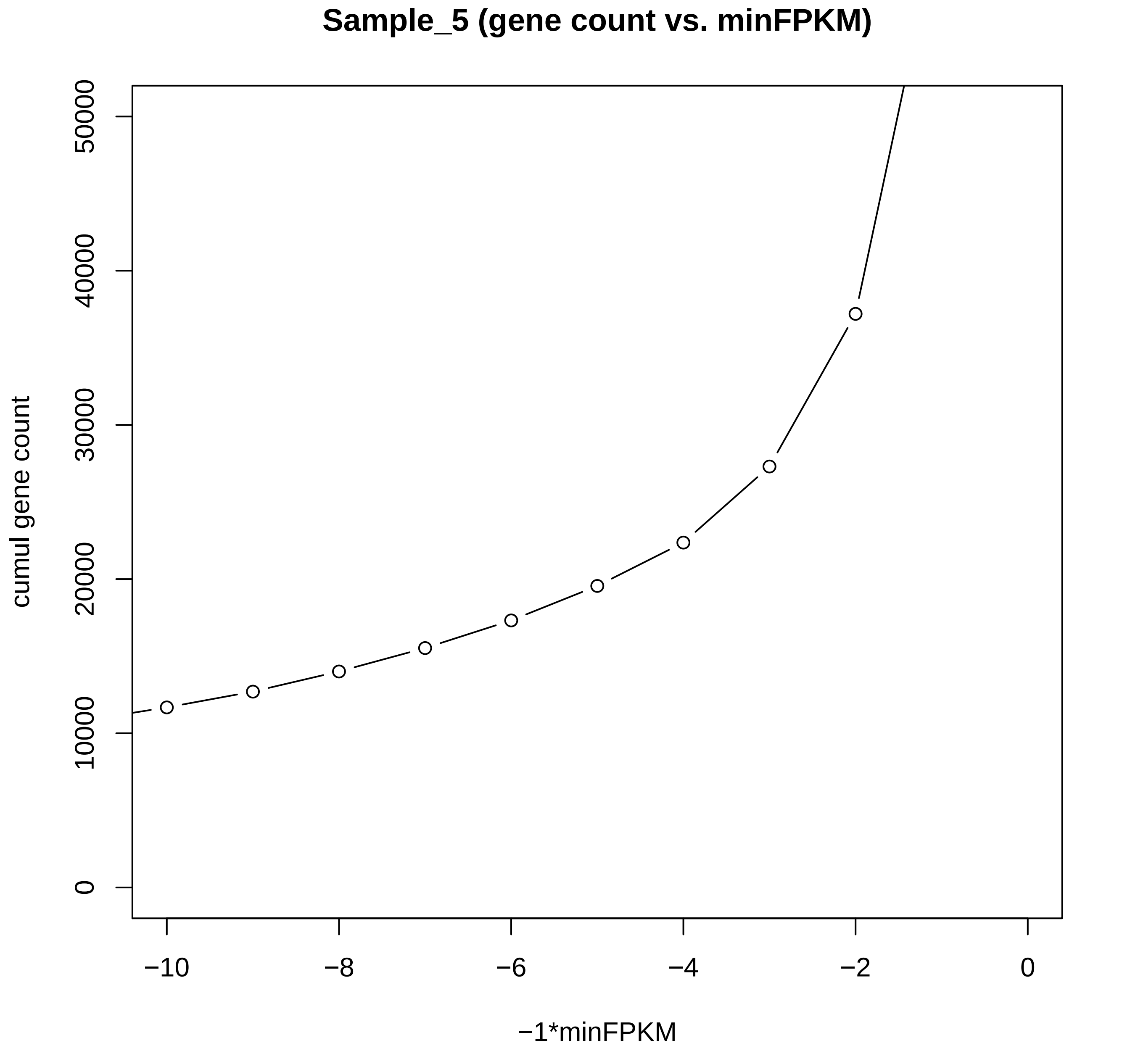

Sample_5 (gene count vs. minFPKM)
cumul gene count
0
10000
20000
30000
40000
50000
−10
−8
−6
−4
−2
0
−1*minFPKM

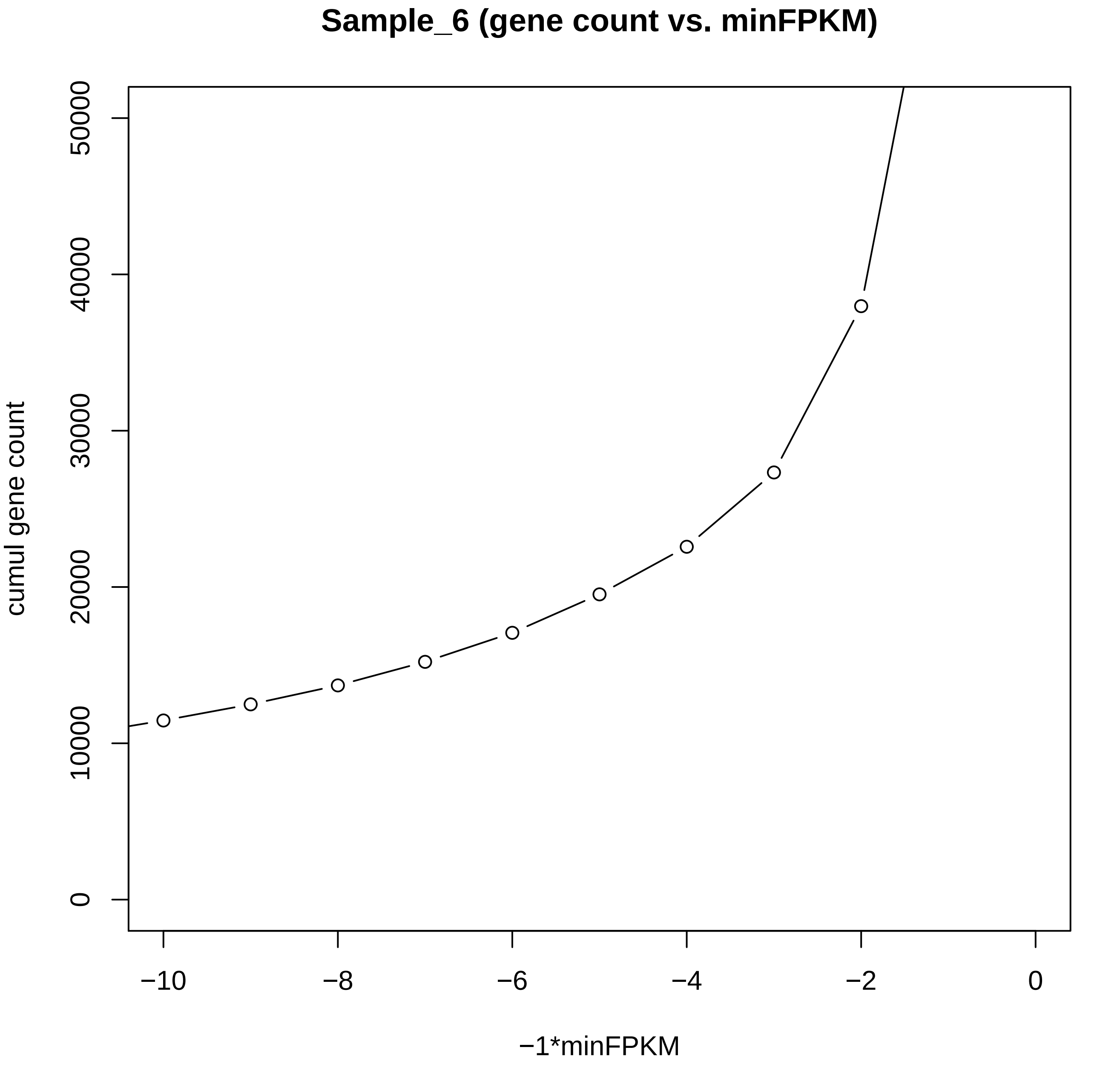
Sample_6 (gene count vs. minFPKM)
cumul gene count
0
10000
20000
30000
40000
50000
−10
−8
−6
−4
−2
0
−1*minFPKM

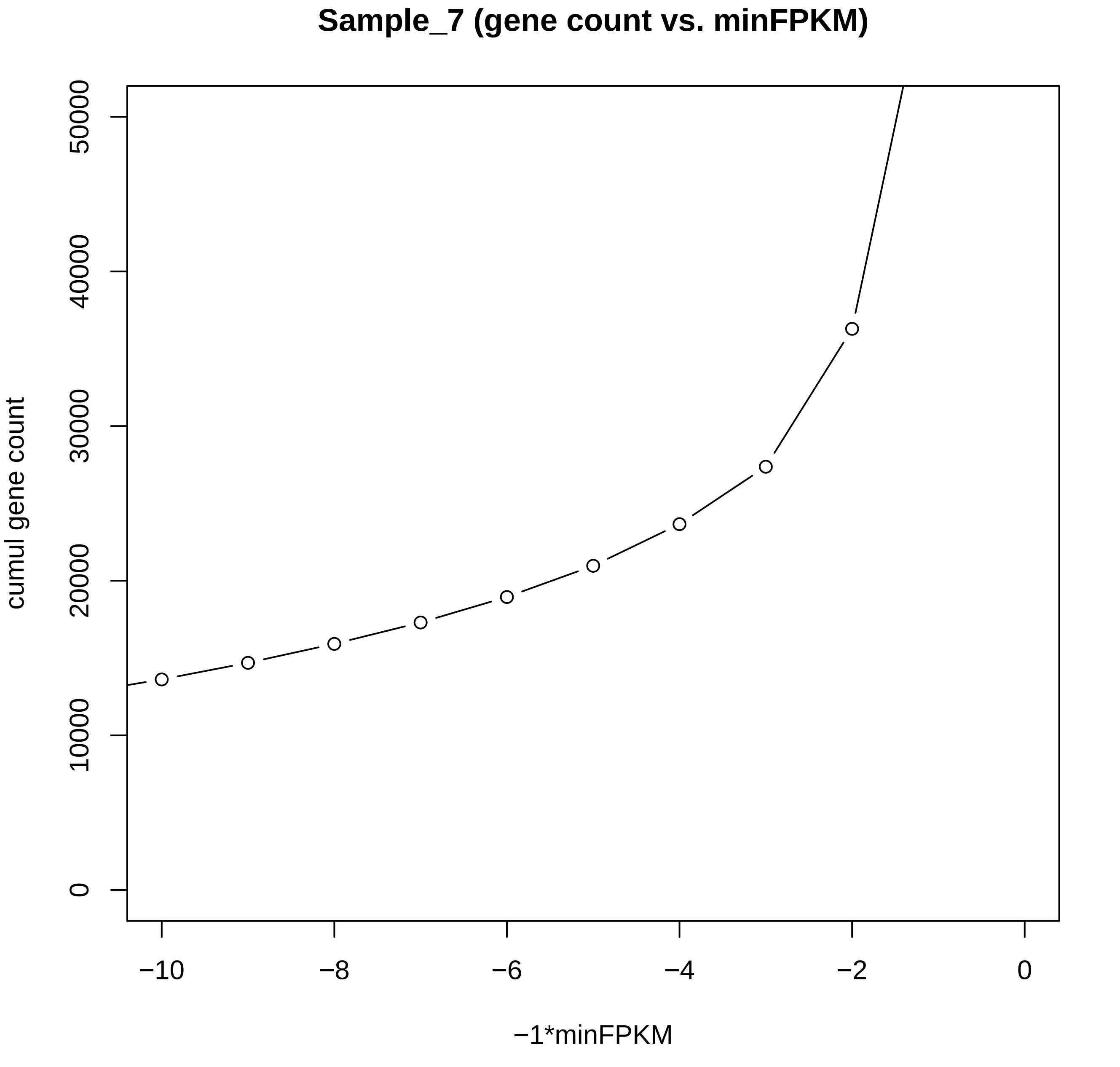

Sample_7 (gene count vs. minFPKM)
cumul gene count
0
10000
20000
30000
40000
50000
−10
−8
−6
−4
−2
0
−1*minFPKM

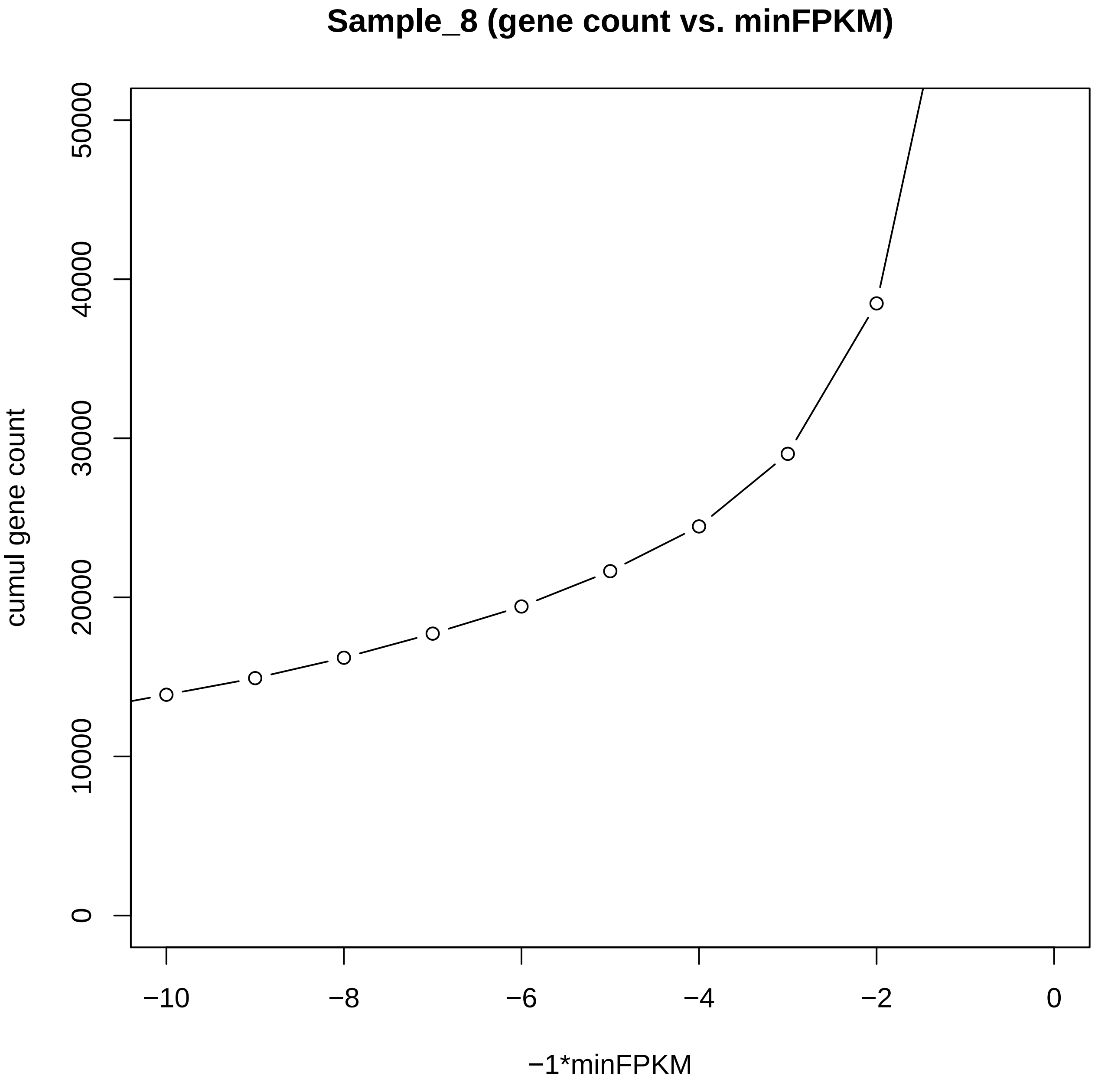

Sample_8 (gene count vs. minFPKM)
cumul gene count
0
10000
20000
30000
40000
50000
−10
−8
−6
−4
−2
0
−1*minFPKM

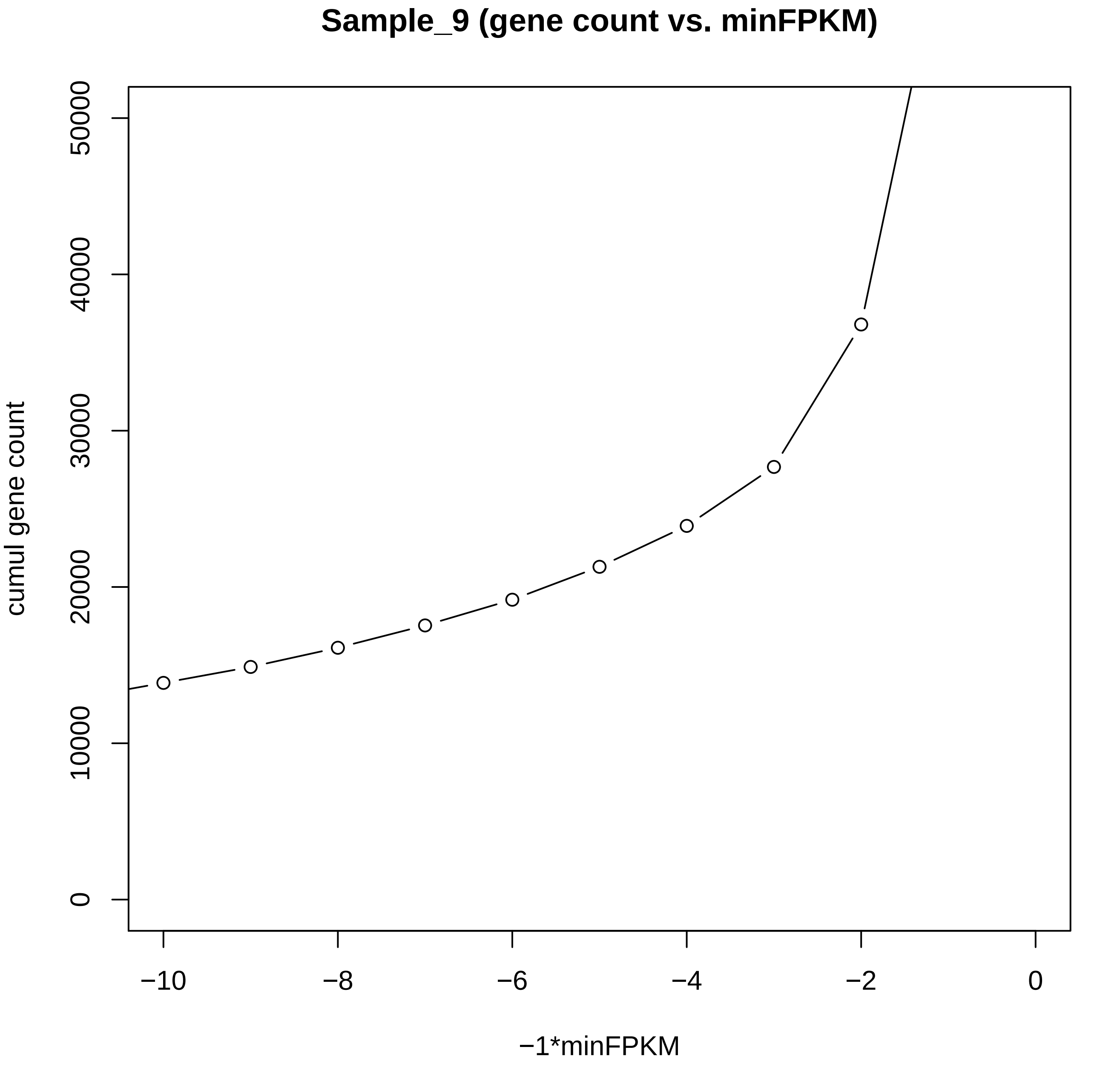

Sample_9 (gene count vs. minFPKM)
cumul gene count
0
10000
20000
30000
40000
50000
−10
−8
−6
−4
−2
0
−1*minFPKM

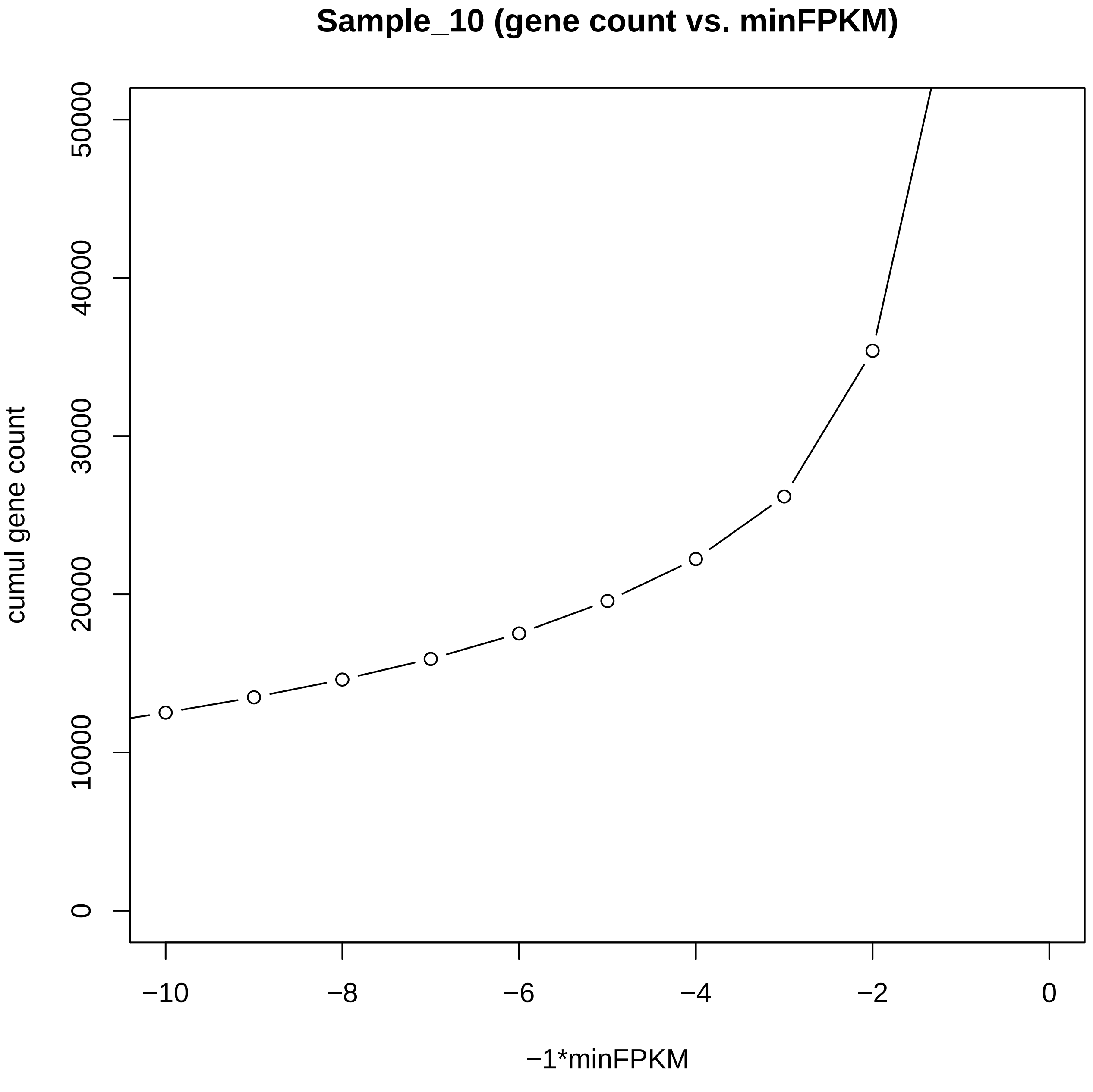
Sample_10 (gene count vs. minFPKM)
cumul gene count
−1*minFPKM
0
10000
20000
30000
40000
50000
−10
−8
−6
−4
−2
0

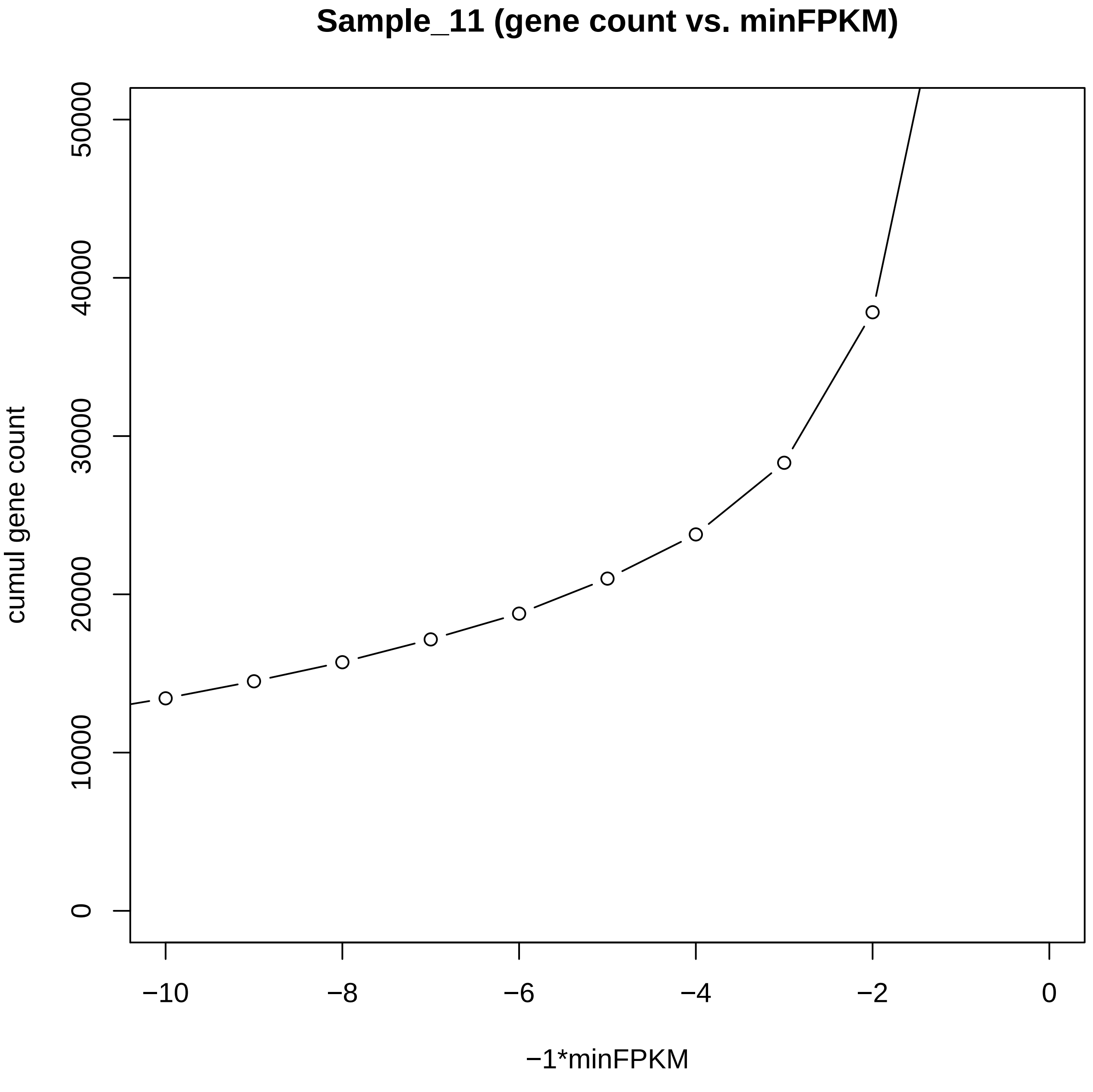

Sample_11 (gene count vs. minFPKM)
cumul gene count
0
10000
20000
30000
40000
50000
−10
−8
−6
−4
−2
0
−1*minFPKM

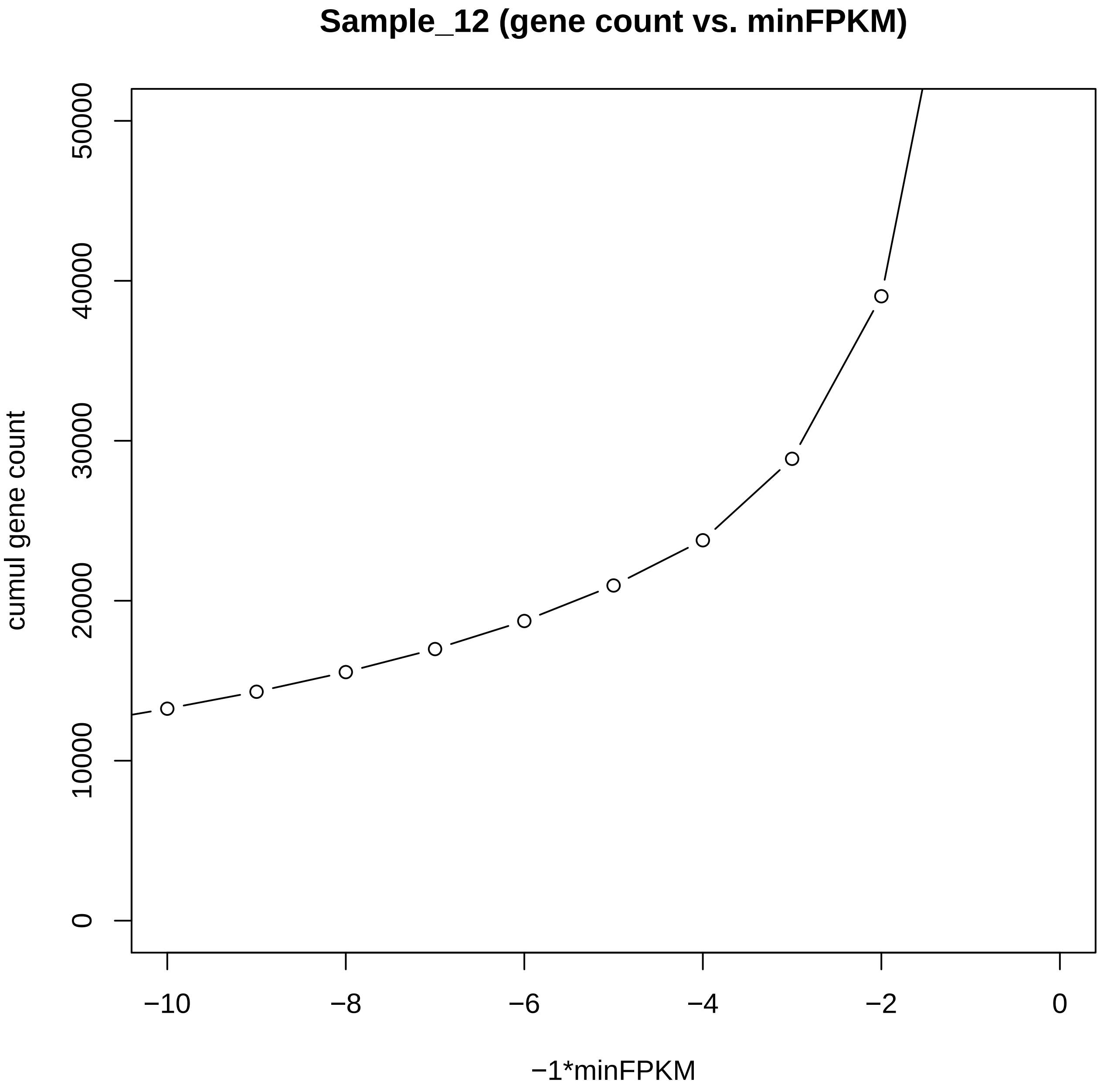

Sample_12 (gene count vs. minFPKM)
cumul gene count
0
10000
20000
30000
40000
50000
−10
−8
−6
−4
−2
0
−1*minFPKM

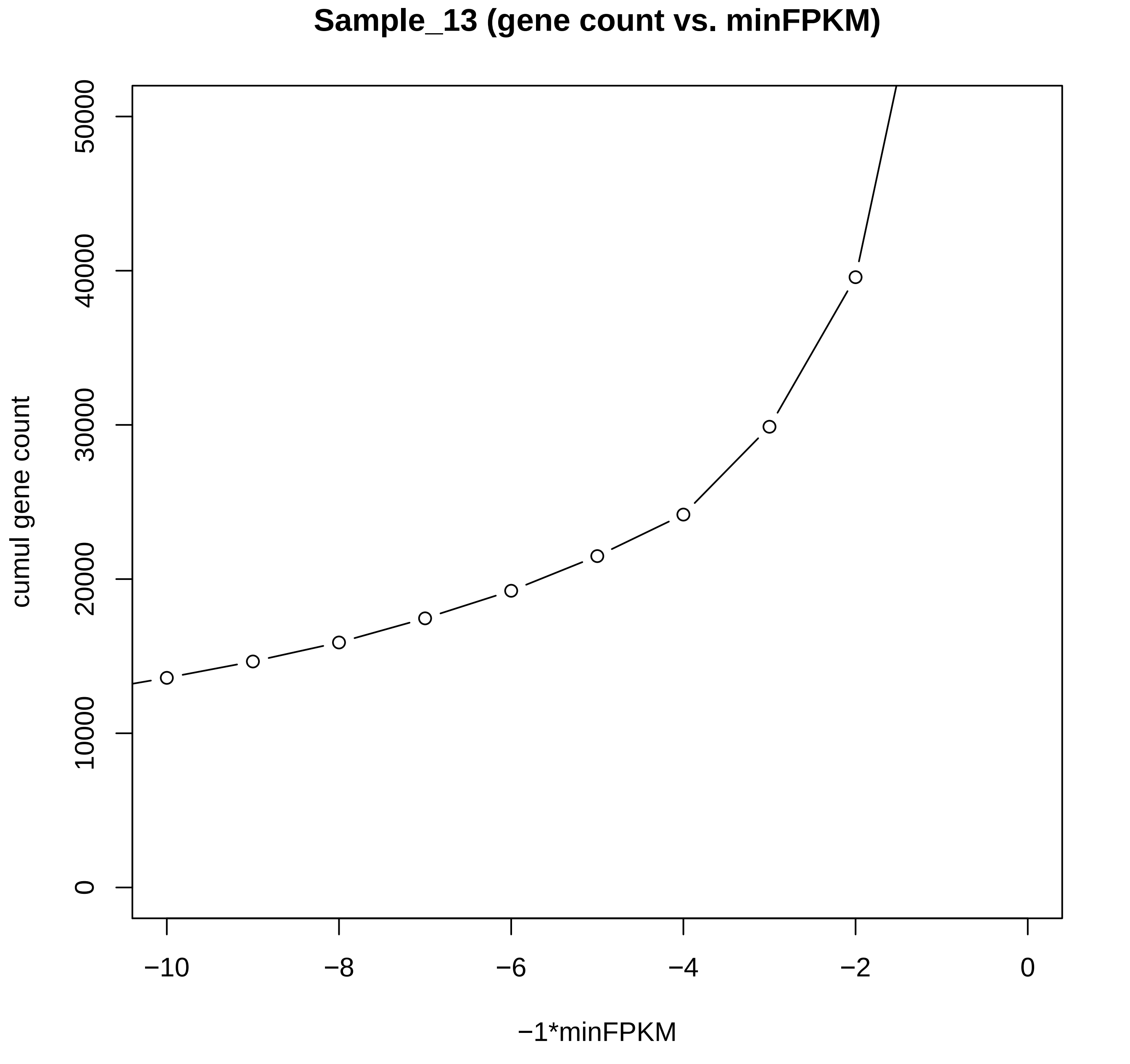

Sample_13 (gene count vs. minFPKM)
cumul gene count
0
10000
20000
30000
40000
50000
−10
−8
−6
−4
−2
0
−1*minFPKM
